# Molecular characterization of *TaSTOP1* homoeologues and their response to aluminium and proton (H^+^) toxicity in bread wheat (*Triticum aestivum* L.)

**DOI:** 10.1186/1471-2229-13-134

**Published:** 2013-09-13

**Authors:** Ana Luísa Garcia-Oliveira, César Benito, Pilar Prieto, Regina de Andrade Menezes, Claudina Rodrigues-Pousada, Henrique Guedes-Pinto, Paula Martins-Lopes

**Affiliations:** 1 Centro de Genómica e Biotecnologia, Instituto de Biotecnologia e Bioengenharia (CGB/IBB), Universidade de Trás-os-Montes e Alto Douro (UTAD), P.O. Box 1013, 5001-801 Vila Real, Portugal; 2 Departamento de Genética, Facultad de Biología, Universidad Complutense de Madrid (UCM), Madrid 28040, Spain; 3 Departamento de Mejora Genética Vegetal, Instituto de Agricultura Sostenible (IAS, CSIC), Alameda del Obispo s/n., P.O. Box 4084, Córdoba 14080, Spain; 4 Instituto de Tecnologia Química e Biológica (ITQB), Universidade Nova de Lisboa, Oeiras, Portugal

**Keywords:** Aluminium, TaSTOP1, *Triticum aestivum L*, *In situ* hybridization, Transcription factor, Transactivation, Homoeologue, pH

## Abstract

**Background:**

Aluminium (Al) toxicity is considered to be one of the major constraints affecting crop productivity on acid soils. Being a trait governed by multiple genes, the identification and characterization of novel transcription factors (TFs) regulating the expression of entire response networks is a very promising approach. Therefore, the aim of the present study was to clone, localize, and characterize the *TaSTOP1* gene, which belongs to the zinc finger family (Cys2His2 type) transcription factor, at molecular level in bread wheat.

**Results:**

*TaSTOP1* loci were cloned and localized on the long arm of homoeologous group 3 chromosomes [3AL (*TaSTOP1-A*), 3BL (*TaSTOP1-B*) and 3DL (*TaSTOP1-D*)] in bread wheat. TaSTOP1 showed four potential zinc finger domains and the homoeologue TaSTOP1-A exhibited transactivation activity in yeast. Expression profiling of *TaSTOP1* transcripts identified the predominance of homoeologue *TaSTOP1-A* followed by *TaSTOP1-D* over *TaSTOP1-B* in root and only predominance of *TaSTOP1-A* in shoot tissues of two diverse bread wheat genotypes. Al and proton (H^+^) stress appeared to slightly modulate the transcript of *TaSTOP1* homoeologues expression in both genotypes of bread wheat.

**Conclusions:**

Physical localization of *TaSTOP1* results indicated the presence of a single copy of *TaSTOP1* on homoeologous group 3 chromosomes in bread wheat. The three homoeologues of *TaSTOP1* have similar genomic structures, but showed biased transcript expression and different response to Al and proton (H^+^) toxicity. These results indicate that *TaSTOP1* homoeologues may differentially contribute under Al or proton (H^+^) toxicity in bread wheat. Moreover, it seems that *TaSTOP1-A* transactivation potential is constitutive and may not depend on the presence/absence of Al at least in yeast. Finally, the localization of *TaSTOP1* on long arm of homoeologous group 3 chromosomes and the previously reported major loci associated with Al resistance at chromosome 3BL, through QTL and genome wide association mapping studies suggests that *TaSTOP1* could be a potential candidate gene for genomic assisted breeding for Al tolerance in bread wheat.

## Background

Aluminium (Al) toxicity is one of the major concerns for crop productivity in acidic soil, accounting for more than 50% of the global arable land [1]. Al is one of the highly abundant elements in the earth crust and under low pH conditions (acidic soils), it is solubilised in the soil in a toxic ionic form that inhibits root elongation. The primary response of plants to Al toxicity is the rapid inhibition of root growth, particularly in the root apex by blocking the process of cell division along with cell elongation, and subsequently inefficient absorption of nutrients and water from soil, resulting in the reduction of plant growth and overall productivity [2].

Plant species differ in the level of Al resistance because they evolved different mechanisms to overcome the selective pressure of Al toxicity imposed under acid soils [3]. These mechanisms can be broadly divided into two categories: Al resistance and Al tolerance. The Al resistance mechanism is often based on the Al exclusion from the root apex or the external detoxification of Al in the rhizosphere through the release of organic acid anions (oxalate, citrate and malate) from the roots limiting Al uptake [4]. On the other hand, the tolerance mechanism may involve the entrance of Al through the roots and its relocation (in the vacuole) or internal detoxification through Al chelating with organic acids (citrate and oxalate) [5]. These mechanisms have been validated at the molecular level, particularly with the functional characterization of the major genes such as *ALMT* (Aluminium-Activated Malate Transporter) and *MATE* (Multidrug and Toxic compound Exudation) in bread wheat (*Triticum aestivum* L.) [6] and sorghum (*Sorghum bicolor* L. Moench) [7], respectively. In addition, genes encoding transporters have been identified through mutational analysis, especially ABC transporters type (*ALS1* and *ALS3*) in *Arabidopsis*[8,9] and bacterial-type ABC transporters (*STAR1* and *STAR2*) in rice (*Oryza sativa* L.) [10]. Recently, a plasma membrane localized transporter *Nrat1* (Nramp aluminum transporter 1) was also found to be associated with Al tolerance particularly to trivalent form of Al in rice [11]. These genes are essential for Al resistance, and seem to be a part of the pathway involved in the secondary level of protection through uptake and redistribution of Al to less sensitive tissues in plants, although their actual function in Al tolerance mechanism in plant is still unclear.

There is strong evidence supporting the important role of regulatory genes (transcriptional factors) in plant tolerance to abiotic stresses [12-15]. Recently, the zinc finger transcription factors, *STOP1* (sensitive to proton rhizotoxicity) and *ART1* (Al resistance transcription factor) have been identified in Al sensitive mutants of *Arabidopsis* and rice, respectively [16,17]. Transcriptome analysis under Al stress revealed that 101 and 31 genes were down-regulated in the Arabidopsis *stop1*[18] and rice *art1* mutants [17], respectively. Interestingly, the major genes related to Al tolerance, particularly *ALMT1* and *MATE1* were regulated by these transcription factors [18,19]. Recently, the contribution of ART1 locus to the variation for Al tolerance in rice has also been identified in QTL analysis [20]. The limited impact of single functional genes in plant stress tolerance has been associated with the polygenic nature of such traits. Thus, the identification and characterization of key regulatory genes that act as master regulators controlling entire response networks would be the most promising and sustainable approach to modify complex traits in plants as they coordinate the expression of many target genes [21].

Wheat is one of the most important natural allopolyploid species, as it is not only directly or indirectly contributing in the food supply for nearly half of the worlds’ population but also can serve as a model plant for other economically important polyploid crop species. It is considered as one of the sensitive crop to Al stress among cereals. Bread wheat, with hexaploid nature comprised from three genomes (AABBDD) are organized in seven homoeologous groups, each homoeologous group has individual gene in triplicate form (from each of A, B and D genomes). Consequently, it is of great interest to reveal how the expressions of homoeologues genes are regulated in hexaploid wheat because theoretically, all the three homoeologues of a gene are assumed to be uniformly expressed. In the previous decade, several studies have been conducted in order to identify the molecular markers (random DNA markers) linked to Al tolerance through QTL mapping and genome-wide association analyses [22-26]. So far only two candidate genes ALMT1 and MATE1 for Al tolerance in wheat have been identified and also mapped on chromosome 4DL using Chinese Spring deletion lines and 4BL through QTL mapping, respectively [22,27,28]. Recently, the three homoeologues of TaMATE1 have been cloned [29], although, no information is available on the expression of respective homoeologues of these candidate genes for Al tolerance in hexaploid wheat. Therefore, in order to improve Al tolerance in bread wheat, identification of three homoeologues of the candidate gene is a promising strategy that could be utilized to develop functional markers for genomic assisted breeding programme in wheat. Herein, we report on the identification, physical localization, and molecular characterization of a novel transcription factor *TaSTOP1* homoeologues genes in bread wheat for Al and proton (H^+^) tolerance.

## Results

###  Cloning and structure of *TaSTOP1*

In order to clone the *STOP1* in wheat, primers from wheat EST showing highest similarity with *Arabidopsis thaliana STOP1* were used to amplify the *TaSTOP1* in bread wheat genotype Barbela 7/72/92. Further, 5’ and 3’ UTR ends of *TaSTOP1* were amplified using RACE as described in material and methods. *TaSTOP1* was amplified in six different bread wheat genotypes and its multiple alignments suggested the mixed amplification from distinct wheat genomes (A, B, and D). Moreover, the comparison of the *TaSTOP1* cDNA sequence with genomic sequence revealed that *TaSTOP1* gene does not contain introns.

**Figure 1:**
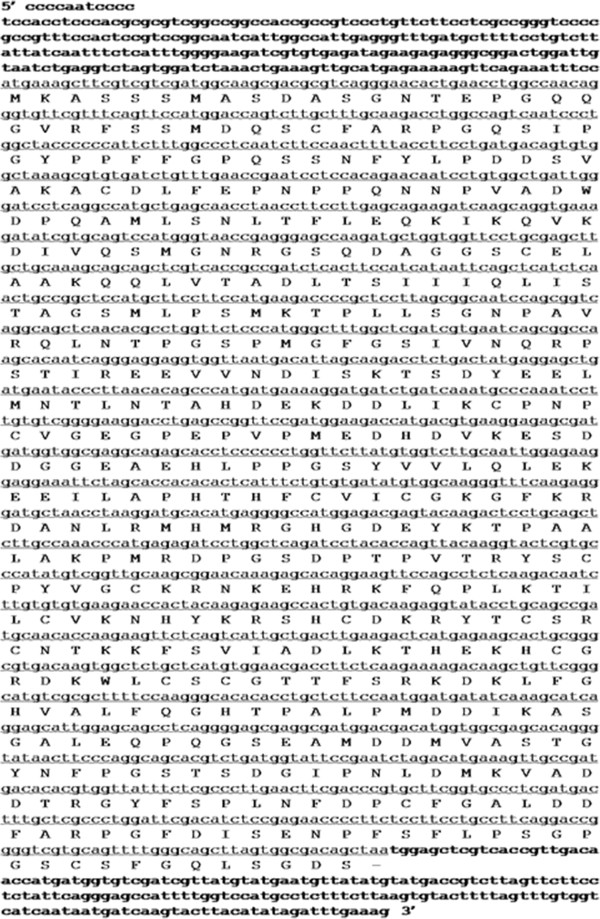
**Nucleotide sequence of *TaSTOP1-D *from Barbela 7/72/92 and its deduced protein (amino acid sequences).** The nucleotide sequence was presented over the deduced amino acid sequence. Note: translation stop codon was noted by-.

The coding region of *TaSTOP1* (*TaSTOP1-A*) from genome A has 1533 bp length and is differentiated from genomes B (*TaSTOP1-B*) and D (*TaSTOP1-D*) by having a 6-bp deletion (1276–1281 positions). *TaSTOP1-B* and *TaSTOP1*-D are differentiated by several SNPs in the respective open reading frame (ORF) [Additional file 1]. *TaSTOP1-D* in bread wheat genotype Barbela 7/72/92 has a full-length cDNA of 1970 bp, containing a coding region of 1539 bp that encodes a polypeptide of 512 amino acids (Figure 1). *TaSTOP1-D* has a molecular weight of 55.8 kD, with a 5.64 isoelectric point (pI). InterProScan function domain analysis suggests that TaSTOP1 belongs to the Cys2His2 zinc finger family protein. Subcellular prediction analysis indicated that TaSTOP1 protein is localized in the nucleus.

### Phylogenetic studies for STOP1 gene among plant species

**Figure 2:**
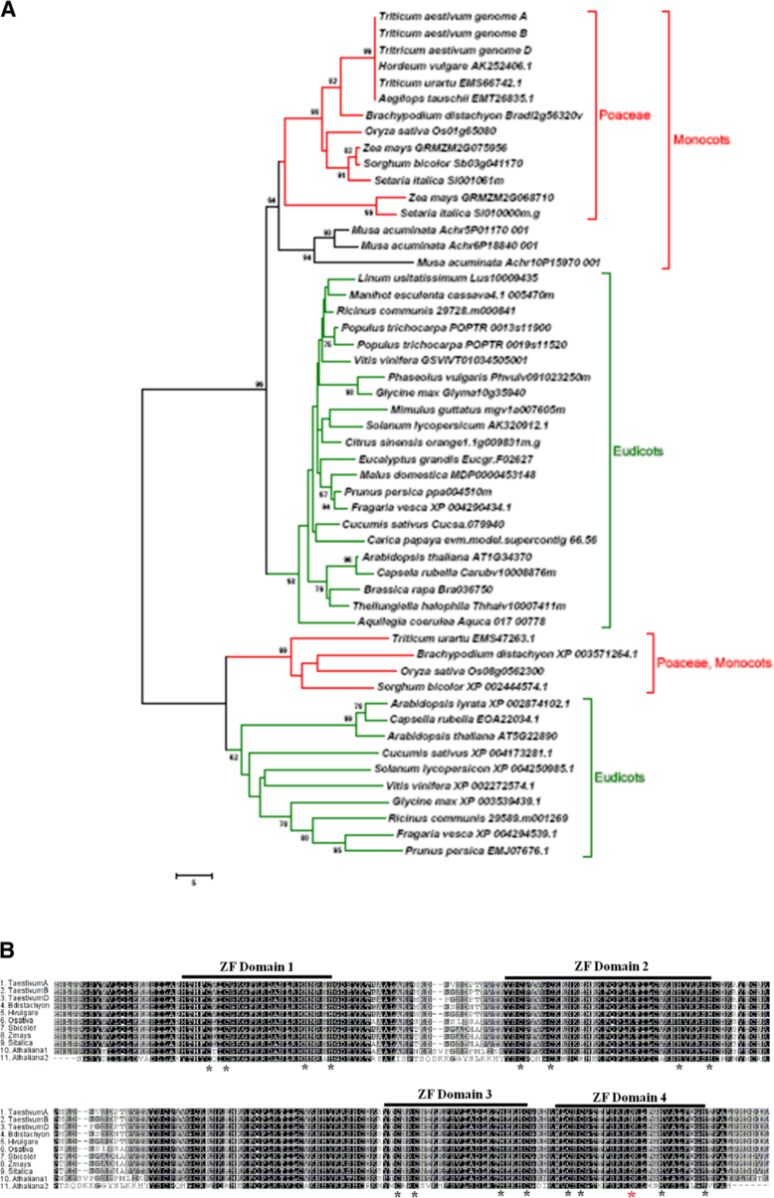
**Phylogenetic tree and multiple alignments of potential Zinc Fingers domain of STOP-like proteins.** Phylogenetic tree based on amino acid sequences showing the relationship of TaSTOP1 with other plant STOP1 type proteins **(A)**. The deduced amino acid sequences were aligned with CLUSTALW. Comparison of ZFs domain of TaSTOP1 (TaSTOP1-A, TaSTOP1-B, TaSTOP1-D) with its homologous from Brachypodium (*B.distachyon*), Barley (*H.vulgare*), rice (*O.sativa*), sorghum (*S.bicolor*), maize (*Z.mays*) foxmillet (*S.italica*), and Arabidopsis (*A.thaliana*1 &*A.thaliana*2) **(B)**. Horizontal bars indicate ZFs domain and asterisks indicate conserved motif of Cys2His2 or Cys2His2-Cys.

Deduced amino acid sequence of TaSTOP1 consists of 510 (TaSTOP1-A) and 512 (TaSTOP1-B and TaSTOP1*-*D) amino acids in bread wheat and the phylogenetic relationship with other STOP-like proteins from 32 different plant species was studied based on their full length sequences. Phylogenetic analysis of STOP like proteins clearly formed two distinct clusters which implied that STOP2 (AT5G22890) was completely distinct from STOP1 (AT1G34370) in Arabidopsis. Furthermore, STOP1 and STOP2 protein from monocotyledons can be clearly distinguished from eudicots (Figure 2A). TaSTOP1 homoeologues from bread wheat showed high similarity with STOP1 from Barley (*Hordeum vulgare*) and *Brachypodium distachium* with identities of 92 and 87%, respectively. Noticeably, the phylogenetic relationship of TaSTOP1 homoeologoues particularly TaSTOP1-A and TaSTOP1-D from bread wheat (AABBDD) displayed the maximum similarity with STOP1 like proteins from *Triticum urartu* (AA) and *Aegilops tauschii* (DD), respectively (Figure 2A).

Sequence alignment of STOP1 protein from bread wheat with its homologous from cereals [*Brachipodium,* barley, rice, sorghum, maize (*Zea mays*) and foxmillet (*Setaria italic*)] and *Arabidopsis*, shows that *TaSTOP1* encodes a putative Cys2His2 zinc finger protein containing four potential zinc finger domains. Three zinc finger domains (ZF1, ZF2, and ZF4) are predicted as the Cys2His2 type, whereas ZF3 is predicted as the Cys2His-Cys or the Cys2His2 type (Figure 2B). Furthermore, sequence alignment revealed that STOP-like proteins share highly conserved regions in the ZF domains. In addition, the STOP1 protein also showed greater sequence conservation in the C-terminus than in the N-terminus among family members. The 6-bp deletion in TaSTOP1-A resulted in a loss of two amino acids [Pro (P) and Gln (Q)] at position 426 without changing the reading frame [Additional file 2]. We also observed in Barbela 7/72/92, two SNPs at position 221 (A replaced by G) and 1186 (G replaced by A) which substituted an Asn (N) by a Ser (S) and Asp (D) by Asn (N) in *TaSTOP1-B* than in both *TaSTOP1-A* and *TaSTOP1-D* homoeologues, respectively [Additional file 1]. Multiple alignment of STOP1 protein revealed that the SNP at position 1186 is located in the zinc finger domain (ZF4) of TaSTOP1 homoeologues (Figure 2B).

### *TaSTOP1* localization on distinct wheat genomes

**Figure 3:**
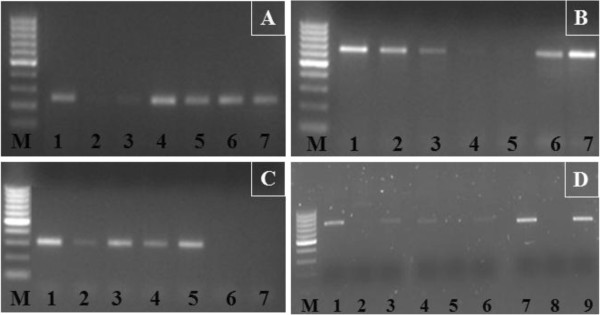
***TaSTOP1 *mapping on homoeologous chromosomes and their arms in bread wheat.***TaSTOP1* mapping on wheat chromosomes from genome A **(A)**, B **(B)** and D **(C)** using nullitetrasomic lines. M: Molecular-weight marker (100bp ladder), 1 to 7: Chinese Spring as control, N3AT3B, N3AT3D, N3BT3A, N3BT3D, N3DT3A, N3DT3B. Arms mapping of *TaSTOP1-A*, *TaSTOP1-B* and *TaSTOP1-D* with ditelosomic lines of homoeologous group 3 **(D)**. 1, 4 and 7: Chinese Spring as a control; 2: Dt3AS; 3: Dt3AL; 5: Dt3BS; 6: Dt3BL; 8: Dt3DS and 9: Dt3DL.

For localization of *TaSTOP1* on homoeologous chromosomes, locus specific primer pairs were designed [Additional file 3] and *TaSTOP1* homoeologues were amplified in a series of nullitetrasomic lines of Chinese Spring wheat along with Chinese Spring as a positive control. On the basis of the presence or absence of PCR products visualized in agarose gels, we observed that *TaSTOP1* genes are located on homoeologous chromosomes 3A, 3B and 3D, and named *TaSTOP1-A, TaSTOP1-B* and *TaSTOP1-D*, respectively (Figure 3A-C). Furthermore, ditelosomic lines of Chinese Spring for homoeologous group 3 chromosomes were also used to assign the *TaSTOP1* on chromosomal arms and confirmed that *TaSTOP1-A*, *TaSTOP1-B* and *TaSTOP1-D* genes are located on the long arm of homoeologous chromosomes 3A, 3B and 3D, respectively (Figure 3D).

**Figure 4:**
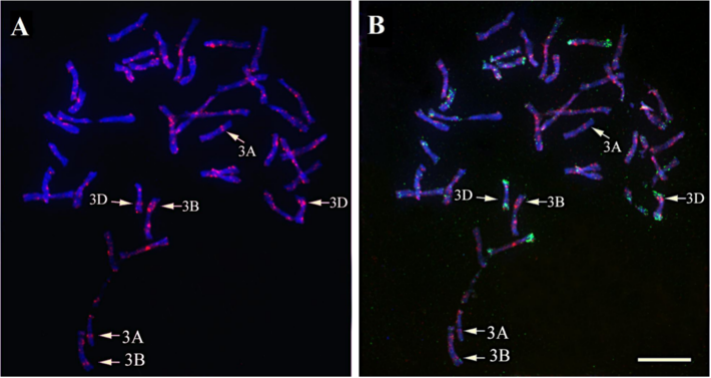
**Physical localization of *TaSTOP1 *on homoeologous chromosome in the root-tip spreads of bread wheat genotype Barbela 7/72/92 using TYR-FISH technique. (A)** Detection of positive hybridization signals of *TaSTOP1* on both chromatids of the long arms of three chromosomes **(B)** and identification of chromosomes after re-probing with GAA- (red) and pAs1 (green). Note: The GAA-satellite sequence identifies A and B genome chromosomes whereas the pAs1 identifies chromosomes from the D genome. Arrow indicates the position of TaSTOP1 on respective chromosome (Scale bar = 10 microm).

In order to further validate these results, physical chromosomal localization of *TaSTOP1* was performed using Tyramide Signal Amplification FISH (Tyr-FISH) technique in the root-tip metaphase chromosome spreads of bread wheat genotype Barbela 7/72/92. For this purpose, a probe was developed by PCR amplification of *TaSTOP1* from genomic DNA of Barbela 7/72/92. FISH with the *TaSTOP1* specific probe on metaphase chromosomes depicted hybridization signals of *TaSTOP1* on both chromatids of the long arms of three chromosomes (Figure 4A) and simultaneous re-probing of chromosome preparations with GAA- (red) and pAs1 (green) identified homoeologous group 3 with positive signals on chromosomes 3AL, 3BL and 3DL (Figure 4B). Some background was also observed but it was distinguished from true hybridization signals because they usually appear as dots without any pattern and not in both chromatids of the same chromosome. Only those signals having the same size and appearance on the same position of both chromatids of one specific chromosome were analyzed as true-positive hybridization signals.

### TaSTOP1 transactivation activity

**Figure 5:**
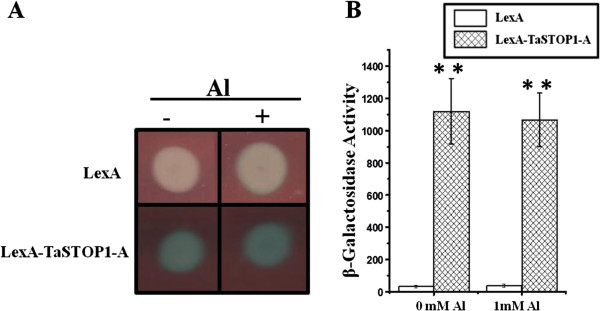
**LexA-TaSTOP1-A exhibits transactivation potential in yeast.** EGY48 cells co-expressing the lexA-TaSTOP1-A and a report cassette bearing the *lacZ* gene driven by a promoter containing lexA binding sites were grown in selective medium in the presence or absence of Al. Qualitative overlay-assay performed on solid medium **(A)** and quantitative measurements of β-galactosidase activity normalized to the A_600_ of cells **(B)**. Values are means (± SD) of six independent transformants. Asterisks denote significant differences as identified by paired *t*-tests (**: P < 0.001).

The nuclear localization of TaSTOP1 protein was predicted by WOLF-PSORT programme [30]. *TaSTOP1* is a putative transcription factor of the Cys_2_His_2_-type Zinc fingers family. The three homoeologues of *TaSTOP1* showed similar genomic structure in bread wheat [Additional file 2]. Therefore, we only proceed to evaluate the transactivation potential of *TaSTOP1* located on genome A (*TaSTOP1-A*). To this aim, we performed a modified yeast one-hybrid assay [31] to evaluate the ability of the lexA-TaSTOP1-A fusion protein to activate the lexA-driven expression of the *lacZ* gene in a heterologous yeast system, the results of which are illustrated in Figure 5. It is shown that lexA-TaSTOP1-A has the potential to transactivate *lacZ* expression either in the presence or absence of Al, further corroborating its potential role as a transcription factor.

### Expression profile of three *TaSTOP1* homoeologues in bread wheat

**Figure 6:**
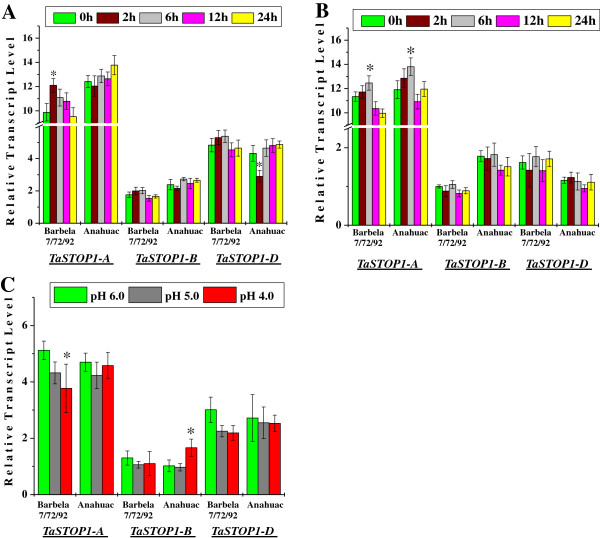
**Relative transcript level (fold change) of *TaSTOP1 *homoeologues genes under Al stress in the roots (A) as well as shoots (B) and under proton (H**^**+**^**) stress in roots (C) of two bread wheat genotypes Barbela 7/72/92 and Anahuac.** Among three homoeologues, the lowest Δ Ct value of a *TaSTOP1* homoeologue [*TaSTOP1-B* in shoots of Barbela 7/72/92 (0 h; as control) and in roots of Anahuac (pH 6.0; as control) for Al and pH stress assay, respectively] was used as calibrator. Values are means (± SD) of three independent replicates. Asterisks indicate significant difference between the control and treatment sample in the respective genome of a genotype (Student´s *t* test, * P < 0.01).

To understand the homoeologue specific expression of *TaSTOP1* in bread wheat, we measured the temporal expression of *TaSTOP1-A, TaSTOP1-B* and *TaSTOP1-D* by Real-time qPCR in the root and shoot tissues of two bread wheat genotypes [Barbela 7/72/92 (Al resistant) and Anahuac (Al sensitive)] grown under Al stress (74 μM Al). The 18S *RNA* expression was used as an internal control. We noticed a biased transcription of homoeologues of *TaSTOP1* gene in the root and shoot tissue of these diverse bread wheat genotypes (Figure 6). The transcript levels of homoeologue *TaSTOP1-A* under control as well as Al treatment were significantly higher than those of *TaSTOP1-B* and *TaSTOP1-D* in root and shoot tissues of both genotypes (depending upon genotypes, fold differences in the transcript abundance were about 5–6 and 6–11 for homoeologue *TaSTOP1-A* versus *TaSTOP1-B* in root and shoot, respectively) whereas expression of homoeologue *TaSTOP1-D* was only two-fold higher than *TaSTOP1-B* in the root tissues of both genotypes (Figure 6A and B). A slight induction (within the two hours of Al exposure) followed by return to basal levels of the homoeologue *TaSTOP1-A* transcripts was observed in the root tissues of Barbela 7/72/92 (Al resistant) whereas in the Anahuac (Al sensitive) under Al stress it was observed a quite stable transcript expression (Figure 6A). The expression of *TaSTOP1-B* was also almost unaltered in the roots of both genotypes under Al stress. Similarly, *TaSTOP1-D* was also constitutively expressed under Al stress in the roots of both genotypes except a rapid (2 h) and significant repression in the Al sensitive genotype Anahuac (Figure 6A). Nevertheless, a quite stable transcript expression of *TaSTOP1* homoeologues was observed in the shoot of both genotypes except homoeologue *TaSTOP1-A* (Figure 6B). Interestingly, and similarly to roots, the expression of *TaSTOP1-A* in the shoots of both genotypes was also slightly induced under Al stress, however, its up-regulation was noticed only after 6 h and return to its basal level (Figure 6B). It is worth noting that the transcript levels of *TaSTOP1-B* of Al tolerant genotype Barbela 7/72/92 are lower in the shoot tissues, compared to the sensitive genotype Anahuac (Figure 6B).

Moreover, to evaluate the transcript expression of *TaSTOP1* homoeologues under protons (H^+^) stress, we also performed the relative quantification of homoeologue specific expression of *TaSTOP1* transcript in root tissues of both genotypes under different levels of pH (6.0, 5.0 and 4.0). A slight gradual decrease in the transcript levels of *TaSTOP1-A* homoeologue was observed in the roots of Barbela 7/72/92 under protons (H^+^) stress, but a quite stable expression was observed in genotype Anahuac (Figure 6C). Furthermore, considerable up-regulation of the homoeologue *TaSTOP1-B* and slight repression of the homoeologue *TaSTOP1-D* transcript level was also observed under low pH (pH 4.0) in the roots of genotype Anahuac and Barbela 7/72/92, respectively (Figure 6C).

## Discussion

So far only *ALMT1* and *MATE* genes have been described as responsible for most of the genotypic variation for Al tolerance and are considered as major genes for Al resistance in bread wheat [32]. These genes have been identified in wheat [6,27] and also in the model plant Arabidopsis [19,33] and major cereals such as barley [34], maize [35], sorghum [7] and rye [36,37]. The mechanisms underlying Al-tolerance in plant species have yet to be fully elucidated, as it seems that Al targets multiple cellular sites such as cell walls, plasma membranes, and cellular processes, like signal transduction pathways and homeostasis mechanisms [2,5,38,39]. Recent evidence suggests that regulatory genes (transcription factors) play a key role in Al detoxification at different cellular levels [16,17].

In the present study, a novel transcription factor gene named TaSTOP1 was cloned from an Al tolerant genotype Barbela 7/72/92 (Figure 1) which was derived from Portuguese bread wheat landrace Barbela through single seed descent method [40]. TaSTOP1 belongs to a member of Cys2His2 zinc finger family proteins that contains four potential zinc finger domains and has highly conserved regions in the zinc finger (ZF) domains (Figure 2B). Like other C2H2-type zinc finger proteins it contains more than one zinc finger motif with highly conserved amino acid sequence for DNA binding [16,41]. Phylogenetic analysis clearly differentiated the STOP1 like proteins from monocots in a group (Figure 2A) and also suggested that genome A and B are more distant from genome D [Additional file 2], as genome D was incorporated recently in comparison to the A and B genomes in bread wheat [42]. In addition, our phylogenetic analysis results also support previous findings at molecular level that the A and D genomes in bread wheat were derived from *T. urartu* and *A. tauschii*, respectively [Additional file 2] [42].

In past, the nullitetrasomic and ditelosomic lines of Chinese Spring wheat have been extensively used in classical and molecular genetic studies for the identification of loci associated with numerous traits including Al tolerance [25,43]. Thus, gene mapping has practical implications in plant genetics and breeding, and the determination of its physical localization is even more precise as it enables us to confirm the exact position of a gene on a chromosome. PCR based mapping of *TaSTOP1* using nullitetrasomic and ditellosomic lines of Chinese Spring wheat revealed that *TaSTOP1* is located on the long arm of wheat homoeologous group 3 chromosomes (Figure 3).

*In situ* hybridization is not only the most direct method for physical localization of genes in chromosomes, but also it is a valuable tool for the identification of copy numbers of a gene in species having a highly complex genome such as the bread wheat. Due to its hexaploid nature, most genes can be found in triplicate with one copy on each genome [44-46]. Tyramide signal amplification FISH (Tyr-FISH) technique has been successfully used for the identification of low copy number DNA sequences in wheat [47]. However, for the successful localization of a gene using the FISH technique, the minimum length of the probe size seems to be crucial. Recently, *RD50* gene has been localized in bread wheat with a probe size of 2 kb [47]. Physical localization of *TaSTOP1* by Tyr-FISH technique not only confirmed the results obtained from PCR based mapping, but also indicated that *TaSTOP1* could be a single copy gene localized on the long arm of homoeologous group 3 chromosomes (Figure 4). In bread wheat, physical localization of single copy gene *Glu*-*1* and *RD50* with FISH technique has been demonstrated on long and short arms of the homoeologous group 1 chromosomes, respectively [47,48]. In the present work, we were able for the first time to localize a gene with a probe size smaller than 2.0 kb demonstrating that the FISH technique can be used to simultaneously anchor homoeologous chromosomes with 1.5 kb probes even in bread wheat.

Subcellular prediction of TaSTOP1 protein in the nucleus is in agreement with data previously reported [18]. Zinc finger motifs are thought to recognize and bind to target DNA sequences, but they are not required for transcriptional activity [49,50]. Our results clearly exhibited the transactivation potential of *TaSTOP1-A* at least in yeast (Figure 5). In present investigation, we observed highly similar genomic structure of *TaSTOP1* genes [Additional files 1 and 2], therefore, it seems that *TaSTOP1* transactivation function is constitutive and may not depend on the presence/absence of Al. The SNPs observed among *TaSTOP1* homoeologues showed minor changes in respective amino acids of putative proteins which could alter the secondary structures by influencing the folding of these proteins (Additional file 1). Therefore, the three homoeologues of the same locus in bread wheat share high sequence similarity that could illustrate the flexibility of a polyploidy species in which due to the mutation in one homoeologue may be compensated for by the homoeologue [29].

Allopolyploidy plays an important role in plant evolution that arises with the merging of two or more genomes into single nucleus which may contribute either equally or disproportionately. However, recent molecular findings confirmed the asymmetric genomic expression pattern in natural and synthetic allopolyploid plant species, but the predominant transcript expression of one genome over the other genome(s) vary from gene to gene [51]. In hexaploid wheat, a uniform level of expression for all the three homoeologues has been reported for approximately 20% of the unigene loci [52], whereas 20-29% genes did not express at least one homoeoallele [52,53]. Thus, the relative contribution of the three homoeologues of *TaSTOP1* gene at transcript expression level was determined under Al and proton (H^+^) stresses in two bread wheat genotypes showing contrasting behaviour for Al toxicity. Transcript expression profiling of *TaSTOP1* homoeologues in root and shoot tissues identified the predominance of *TaSTOP1-A* homoeologue followed by *TaSTOP1-D* over *TaSTOP1-B* in root and only predominance of *TaSTOP1-A* in shoot tissues of both genotypes under control and stress (Al and pH) conditions (Figure 6). Similarly, among the three homoeologues, the higher expression of one homoeologue of a MAD box transcription factor and Spa gene have also been observed in bread wheat [54,55]. Although, the presence of *cis* elements within the 5' untranslated region of a gene is unusual, it is not aberrant or abnormal [56]. Surprisingly, sequence analysis of 5' UTR of *TaSTOP1* genes differentiated the *TaSTOP1-A* homoeologue from *TaSTOP1-B* and *TaSTOP1-D* due to the presence of a pyrimidine-rich stretch and absence of light responsive element [Additional file 4]. In tomato, the deletion of the 5' UTR containing pyrimidine-rich stretch from the HMG2 promoter reduced the level of HMG2 gene expression by a factor of 10 [56]. Therefore, among the three homogeologues in bread wheat, the up- or down-regulation of the expression of specific homoeologue could be the result from either the dominancy of ancestral diploid donor parent or early polyploidization-cis-regulatory variation.

Interestingly, the time-dependent Al-responsive expression of TaSTOP1 homoeologues observed in the root tissues of two bread wheat showing contrasting phenotypes for Al toxicity suggests a putative role for TaSTOP1 in Al resistance (Figure 6A and B). Al responsive *STOP1* expression has also been reported in *Arabidopsis*[16], alfalfa [57] and common bean [58]. Similarly to Arabidopsis, the transcript expression of homoeologues of *TaSTOP1* in the roots of diverse bread wheat genotypes was also modulated in response to proton stress [16]. It is noticeable that genotype Barbela 7/72/92 is highly resistant to Al toxicity compared with Anahuac, but in the absence of Al under low pH we did not observe significant differences between these genotypes for root growth [Additional file 5]. It is worth noticing that the three homoeologues of *TaSTOP1* have similar genomic structures, but showed a different transcript expression in response to Al and proton (H^+^) stress. These results may reveal that the homoeologues of TaSTOP1 are differentially contributing to Al or proton (H^+^) tolerance in bread wheat. Further work is in progress in order to definitely establish if these genes play a significant role in aluminium tolerance.

## Conclusions

Bread wheat has wide genotypic variation for Al resistance [23,40] and the role of several chromosomes such as chromosome arms 2DL, 3DL, 4BL, 4DL, 6AL, 7AS and chromosome 7D, in Al tolerance has been revealed in classical genetic studies through chromosome manipulation in wheat [43]. Furthermore, several QTL associated with Al tolerance in bread wheat have also been reported by many researchers [22,24-26]. Contrarily to Arabidopsis and rice, due to the paucity of *a priori* candidate genes in wheat only two major QTL located on chromosome 4DL and 4BL have yet been elucidated at molecular level, showing the co-segregation with candidate genes *TaALMT1* homoeologue (4DL) and *TaMATE1* homoeologue (4BL), respectively [22,27]. The classical studies using chromosomal manipulation as well as recent QTL mapping and genome-wide association analysis have also detected loci associated with Al resistance on homoeologous group 3 chromosomes (3A, 3B and 3D) in bread wheat [23-25,43]. Recently, the role of a zinc finger transcription factor ART1 identified through mutational analysis in rice has also been shown in natural variation of Al tolerance in rice, earlier which was suggested that it was not involved in Al tolerance [20,32].

In the present investigation, we cloned and characterized the novel candidate genes *TaSTOP1* in bread wheat. The homoeologues of *TaSTOP1* exhibited similar genomic structures, but showed biased transcript expression and different response to Al and proton (H^+^) toxicity. Furthermore, *TaSTOP1-B* homoeologue from Al tolerant genotypes Barbela 7/72/92 and Viloso Mole not only showed highest similarity but also contain the same SNP located in ZF4 domain than Al sensitive genotypes Anahuac, Chinese Spring and Saloio [Additional file 2] [40]. Finally, in order to correlate with Al tolerance, it would be very interesting either to functionally characterize or further verify the role of TaSTOP1 because gene(s) underlying the QTL on homoeologous group 3 chromosomes particularly 3BL has not been so far identified in this important cereal.

## Methods

### Plant material and growth condition

The seedlings of selected bread wheat genotypes Barbela 7/72/92, Anahuac, Chinese Spring, Ruivo, Viloso Mole and Saloio classified in relation to Al tolerance [40] were grown in hydroponic solution (0.4 mM CaCl_2_, 0.65 mM KNO_3_, 0.25 mM MgCl_2_.6H_2_O, 0.1 mM (NH_4_)_2_SO_4_ and 0.04 mM NH_4_NO_3_) and kept in controlled growth chamber under 14 h/26°C day and a 10 h/22°C night regime, with a light intensity of 150 μmol photons m^-2^s^-1^ and a relative humidity of 65%.

### Al stress assay

Four days old seedlings of Al resistant bread wheat genotype Barbela 7/72/92 and Al susceptible genotype Anahuac growing in hydroponic solution with pH 4.0 were shifted in fresh nutritive solution having 74 μM Al whereas control seedling were raised in only fresh hydroponic solution. For root re-growth measurement, after 24 h, roots were immersed in 0.1% eriochrome cyanine R (Sigma, Germany) dye solution for 10 minutes and prior to measurement; seedlings were allowed to grow in fresh nutritive solution for 48 h. For visual detection of Al accumulation in roots, hematoxylin assay was performed [59] and root samples were observed under microscope.

### DNA and RNA extraction

Total genomic DNA was isolated from young leaf samples using the DNeasy Plant Mini Kit (Qiagen, Germany) and total RNA was extracted using Trizol method followed by purification using PureLink™ RNA Mini Kit (Ambion, Invitrogen, USA). The first-strand cDNA was synthesized in a final volume of 20 μl reaction containing: 1 μg RNA, 2 μl 10× RT buffer, 0.8 μl of 25× dNTP mix (100 mM), 2 μl of 10× RT random primers and 1 μl of Multiscribe reverse transcriptase (Applied Biosystems, USA).

### Gene structure and cloning of full-length cDNA

The *Arabidopsis thaliana* AtSTOP1 (AT1G34370) protein sequence was retrieved from TAIR database (http://www.arabidopsis.org) and was used as the query sequence to search the GenBank wheat expressed sequence tag (EST) database. The homologous sequence of *STOP1* in wheat was obtained via tblastn in NCBI (http://www.ncbi.nlm.nih.gov). Homologous wheat EST clones showing highest similarity with *Arabidopsis STOP1* were retrieved. *TaSTOP1* (wheat EST) specific primers were designed to perform the sequential analysis in bread wheat genotype Barbela 7/72/92 [Additional file 3]. Total RNA from the seedlings of Barbela 7/72/92 and Anahuac was employed to obtain the full-length cDNA of *TaSTOP1* including 5’ UTR and 3’ UTR using Rapid Amplification of cDNA Ends (RACE) (SMARTer™ RACE cDNA Amplification Kit, clontech, USA). The genomic sequence of *TaSTOP1* was obtained in six different bread wheat genotypes, namely Barbela 7/72/92, Anahuac, Ruivo, Viloso Mole, Saloio and Chinese Spring [Additional file 6].

### Molecular analysis and construction of phylogenetic tree

The Pfam (http://www.sanger.ac.uk/Software/Pfam/search,shtml) software was used to identify potential domains and WOLF-PSORT (http://www.psort.org) was used to predict the intracellular localization of the TaSTOP1 protein. Fifty two STOP1 like protein sequences were collected from 32 different plant species including TaSTOP1 homoeologues from bread wheat genotype Barbela 7/72/92 using the Basic Local Alignment Search Tool (BLAST) programme from NCBI (http://blast.ncbi.nlm.nih.gov/Blast.cgi) [Additional file 7]. Phylogenetic tree was constructed by MEGA4 software using neighbor-joining method [60].

### Mapping of *TaSTOP1* gene in wheat genome

All the nullitetrasomic and ditelosomic lines of Chinese Spring wheat except for the homoeologous group 2, 4 and 6 chromosomes used in present investigation were available in our lab, whereas remaining stocks were kindly provide by Prof. Adam J. Lukaszewski at Department of Botany and Plant Sciences, University of California, USA. On the basis of *TaSTOP1* alignment, different pairs of specific primers for each *TaSTOP1* homoeologue were designed [Additional file 3]. *TaSTOP1* homoeologues (*TaSTOP1-A*, *TaSTOP1-B* and *TaSTOP1-D*) were amplified in the nullitetrasomic (N3AT3B, N3AT3D, N3BT3A, N3BT3D, N3DT3A and N3DT3B) and ditelosomic [Dt 3AL and 3AS (long and short arm), Dt 3BL and 3BS, Dt 3DL and 3DS] lines of Chinese Spring wheat. *TaSTOP1* homoeologues were assigned to the chromosomal arms based on the presence/absence of the PCR amplification products.

Physical localization of *TaSTOP1* was performed by *in situ* hybridization. Probe was prepared by PCR amplification of the 1.5 kb genomic region of *TaSTOP1* from Barbela 7/72/92. Chromosome spreads from the root tips of Barbela 7/72/92 seedlings at mitotic metaphase, probe labelling and hybridization mixture were carried as described by Prieto *et al.*[61]. Probe labelling was confirmed by dot-blot and detection of hybridization signals was carried out using the Tyramide Signal Amplification Kit (TSA™, PerkinElmer Life and Analytical Sciences, Inc., Waltham, MA, USA). To identify wheat chromosomes with positive signals, samples were re-hybridized using simultaneously the pAS1 repetitive sequence and GAA-satellite sequence as probes [62,63]. The GAA-satellite sequence identifies all the A and B wheat chromosomes [62] whereas the pAs1 identifies chromosomes from the D genome [63]. Individual slides were observed under a Nikon Eclipse 80i microscope (Nikon Instruments Europe BV, UK). Images were captured with a Nikon CCD camera using the appropriate Nikon 3.0 software and processed with Photoshop 4.0 software (Adobe Systems Inc., San Jose, California, USA).

### Transactivation assay

*TaSTOP1* was amplified by PCR from Barbela 7/72/92 using oligonucleotide primers containing restriction site for *Kpn* I [Additional file 3]. The *Kpn* I digested *TaSTOP1* fragment was cloned in frame with the *lexA* gene in the plasmid YCp91 [31]. Transformation of the ligated products was performed in the *Escherichia coli* strain *XL1-Blue recA1 endA1 gyrA96 thi-hsdR17 supE44 relA1 lac* [*F’proAB lacI*^*q*^*ZDM15 Tn10* (*Tet*^*r*^)] (Stratagene, USA) and the positive clones were sequenced. The recombinant plasmid YCp91-*TaSTOP1-A* was transformed in the yeast strain EGY48, which harbours the plasmid pSH18.84 encoding the *lacZ* gene under the control of a promoter containing LexA – responsive *cis* elements. Transformants were grown in selective media and β-galactosidase measurements were performed as previously described [64,65].

### Analysis of *TaSTOP1* expression level

For Al assay, seedlings of two bread wheat genotypes Barbela 7/72/92 and Anahuac showing contrasting response to Al toxicity, were raised in hydroponic nutrient solution with pH 4.0 during four days and further, were shifted to fresh nutrient solution with (74 μM Al in the form of AlCl_3_·6H_2_O; stress treatment) or without Al (control treatment). Both root and shoot tissues were collected separately after treatment at specific time points (0, 2, 6, 12 and 24 h) from control and Al stress imposed seedlings. For pH assay, four days old seedlings of both genotypes grown in hydroponic nutritive solution with pH 6.0 were transferred in fresh nutritive solutions having different levels of pH (pH 6.0, pH 5.0 and pH 4.0). Root samples of both genotypes from each treatment were collected after 0 h and 6 h exposure to different levels of pH.

Three biological replicates of each sample were prepared and duplicate quantitative assays were performed for each cDNA sample. *TaSTOP1* homoeologues gene expression pattern was determined using the SYBR Premix Ex Taq (Takara, Japan) and the ABI 7500 Real-Time FAST PCR System (Applied Biosystems, USA). The 2^-ΔΔC^_T_ method [66] was used to quantify the relative expression levels of *TaSTOP1* homoeologues in comparison to *18SRNA* endogenous control.

### Accession numbers

Sequence data from this article can be found in the GenBank database under the following accession numbers: Barbela 7/72/92 (TaSTOP1-A: GenBank number KF034793; TaSTOP1-B: GenBank number KF034794; TaSTOP1-D: GenBank number KF034795), Anahuac (TaSTOP1-A: GenBank number KF034796; TaSTOP1-B: GenBank number KF034797; TaSTOP1-D: GenBank number KF034798), Viloso Mole (TaSTOP1-A: GenBank number KF034801; TaSTOP1-B: GenBank number KF034802; TaSTOP1-D: GenBank number KF034803), Saloio (TaSTOP1-A: GenBank number KF034804; TaSTOP1-B: GenBank number KF034805; TaSTOP1-D: GenBank number KF034806), Chinese Spring (TaSTOP1-A: GenBank number KF034799; TaSTOP1-B: GenBank number KF034800) and Ruivo (TaSTOP1-D: GenBank number KF034807).

## Abbreviations

ALMT: Aluminium-activated malate transporter; MATE: Multidrug and toxic compound exudation; ALS: Al-sensitive; STAR: Sensitive to Al rhizotoxicity; Nrat1: Nramp aluminum transporter 1; STOP: sensitive to proton rhizotoxicity; ART: Al resistance transcription factor; QTL: Quantitative trait loci/locus; SNP: Single nucleotide polymorphism; ORF: Open reading frame; ZF: Zinc finger domains; FISH: Fluorescent *in situ* hybridization.

## Competing interests

The authors declared that they have no competing interests.

## Authors’ contributions

ALGO performed most of the experiments and drafted the manuscript; PP helped in *in-situ* hybridization assay; RAM helped in transactivation assay and CB guided for cloning, chromosomal localization and figure preparation, CRP, HGP and PML coordinated the experiments and helped in finalizing the manuscript. Finally, all authors read and approved the final manuscript.

## Supplementary Material

Additional file 1 **Multiple alignments of the homoeologues of *TaSTOP1 *in bread wheat genotype Barbela 7/72/92.** Click here for file

Additional file 2 **Phylogenetic tree and multiple alignments of *TaSTOP1 *homoeologues in different species of wheat including some bread wheat genotypes.** Click here for file

Additional file 3 Detail of primers used in present investigation. Click here for file

Additional file 4 **Alignment of homoeologues of *TaSTOP1 *5UTR region.** Click here for file

Additional file 5 Physiological characterization of Barbela 7/72/92 and Anahuac roots under Al stress. Click here for file

Additional file 6 Nucleotide and protein sequence of TaSTOP1 in different bread wheat genotypes. Click here for file

Additional file 7 Detail of STOP like proteins in different plant species used for phylogenetic analysis. Click here for file
