# Supplementary figures and images for: Molecular characterization of TaSTOP1 homoeologues and their response to aluminium and proton (H+) toxicity in bread wheat (Triticum aestivum L.)
Source: BMC Plant Biol. 2013 Sep 13;13:134. doi: 10.1186/1471-2229-13-134 (PMC3848728; doi:10.1186/1471-2229-13-134)

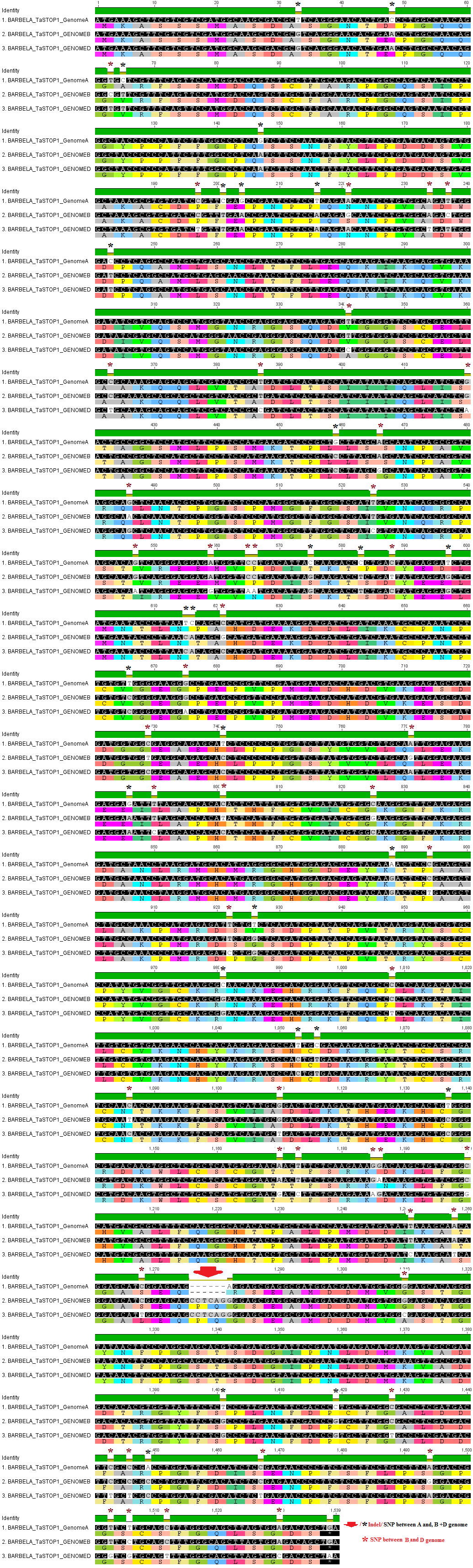

Supplement: Additional file 1 — Multiple alignments of the homoeologues of TaSTOP1 in bread wheat genotype Barbela 7/72/92. [file 1471-2229-13-134-S1.png]
