# Supplementary material for: Molecular characterization of TaSTOP1 homoeologues and their response to aluminium and proton (H+) toxicity in bread wheat (Triticum aestivum L.)
Source: BMC Plant Biol. 2013 Sep 13;13:134. doi: 10.1186/1471-2229-13-134 (PMC3848728; doi:10.1186/1471-2229-13-134)
Supplement: Additional file 2 — Phylogenetic tree and multiple alignments of TaSTOP1 homoeologues in different species of wheat including some bread wheat genotypes. [file 1471-2229-13-134-S2.doc]

**Additional File 2**A: Phylogenetic analysis of *TaSTOP1* homoeologues genes in different species of wheat including some bread wheat genotypes.

TaSTOP1A_Barbela MKASSSMASDASGNTDPGQQGARFSSMDQSCFARPGQSIPGYPPFFGPQSSNFYLPDDSV 60

TaSTOP1A_Anahuac MKASSSMASDASGNTDPGQQGARFSSMDQSCFARPGQSIPGYPPFFGPQSSNFYLPDDSV 60

TaSTOP1B_Barbela MKASSSMASDASGNTEPGQQGVRFSSMDQSCFARPGQSIPGYPPFFGPQSSNFYLPDDSV 60

TaSTOP1B_Anahuac MKASSSMASDASGNTEPGQQGVRFSSMDQSCFARPGQSIPGYPPFFGPQSSNFYLPDDSV 60

TaSTOP1D_Barbela MKASSSMASDASGNTEPGQQGVRFSSMDQSCFARPGQSIPGYPPFFGPQSSNFYLPDDSV 60

TaSTOP1D_Anahuac MKASSSMASDASGNTEPGRQGVRFSSMDQSCFARPGQSIPGYPPFFGPQSSNFYLPDDSV 60

***************:**:**.**************************************

TaSTOP1A_Barbela AKACDPFEPNPPQNNPVADWDPQAMLSNLTFLEQKIKQVKDIVQSMGNRGSQDVGGSCEL 120

TaSTOP1A_Anahuac AKACDPFEPNPPQNNPVADWDPQAMLSNLTFLEQKIKQVKDIVQSMGNRGSQDVGGSCEL 120

TaSTOP1B_Barbela AKACDPFEPNPPQSNPVADWDPQAMLSNLTFLEQKIKQVKDIVQSMGNRGSQDVGGSCEL 120

TaSTOP1B_Anahuac AKACDPFEPNPPQSNPVADWDPQAMLSNLTFLEQKIKQVKDIVQSMGNRGSQDVGGSCEL 120

TaSTOP1D_Barbela AKACDLFEPNPPQNNPVADWDPQAMLSNLTFLEQKIKQVKDIVQSMGNRGSQDAGGSCEL 120

TaSTOP1D_Anahuac AKACDLFEPNPPQNNPVADWDPQAMLSNLTFLEQKIKQVKDIVQSMGNRGSQDAGGSCEL 120

***** *******.***************************************.******

TaSTOP1A_Barbela AAKQQLVTADLTSIIIQLISTAGSMLPSMKTPLLSSNPAVRQLNTPGSPMGFGSIVNQRP 180

TaSTOP1A_Anahuac AAKQQLVTADLTSIIIQLISTAGSMLPSMKTPLLSSNPAVRQLNTPGSPMGFGSIVNQRP 180

TaSTOP1B_Barbela AAKQQLVTADLTSIIIQLISTAGSMLPSMKTPLLSSNPAVRQLNTPGSPMGFGSIVNQRP 180

TaSTOP1B_Anahuac AAKQQLVTADLTSIIIQLISTAGSMLPSMKTPLLSSNPAVRQLNTPGSPMGFGSIVNQRP 180

TaSTOP1D_Barbela AAKQQLVTADLTSIIIQLISTAGSMLPSMKTPLLSGNPAVRQLNTPGSPMGFGSIVNQRP 180

TaSTOP1D_Anahuac AAKQQLVTADLTSIIIQLISTAGSMLPSMKTPLLSSNPAVRQLNTPGSPMGFGSIVNQRP 180

***********************************.************************

TaSTOP1A_Barbela STVREEMVPDITKTPDYEDLMNTLNPAHDEKDDLIKCPNPCVGEGPEPVPMEDHDVKESD 240

TaSTOP1A_Anahuac STVREEMVPDITKTPDYEDLMNTLNPAHDEKDDLIKCPNPCVGEGPEPVPMEDHDVKESD 240

TaSTOP1B_Barbela STVREEMVPDISKTSDYEELMNTLNTAHDEKDDLIKCPNPCVGEGPEPVPMEDHDVKESD 240

TaSTOP1B_Anahuac STVREEMVPDISKTSDYEELMNTLNTAHDEKDDLIKCPNPCVGEGPEPVPMEDHDVKESD 240

TaSTOP1D_Barbela STIREEVVNDISKTSDYEELMNTLNTAHDEKDDLIKCPNPCVGEGPEPVPMEDHDVKESD 240

TaSTOP1D_Anahuac STIREEVVNDISKTSDYEELMNTLNTAHDEKDDLIKCPNPCVGEGPEPVPMEDHDVKESD 240

**:***:* **:**.***:******.**********************************

TaSTOP1A_Barbela DGGEAEHLPPGSYVVLQLEKEEILAPHTHFCVICGKGFKRDANLRMHMRGHGDEYKTPAA 300

TaSTOP1A_Anahuac DGGEAEHLPPGSYVVLQLEKEEILAPHTHFCVICGKGFKRDANLRMHMRGHGDEYKTPAA 300

TaSTOP1B_Barbela DGGEAEHLPPGSYVVLQLEKEEILAPHTHFCVICGKGFKRDANLRMHMRGHGDEYKTPAA 300

TaSTOP1B_Anahuac DGGEAEHLPPGSYVVLQLEKEEILAPHTHFCVICGKGFKRDANLRMHMRGHGDEYKTPAA 300

TaSTOP1D_Barbela DGGEAEHLPPGSYVVLQLEKEEILAPHTHFCVICGKGFKRDANLRMHMRGHGDEYKTPAA 300

TaSTOP1D_Anahuac DGGEAEHLPPGSYVVLQLEKEEILAPHTHFCVICGKGFKRDANLRMHMRGHGDEYKTPAA 300

************************************************************

TaSTOP1A_Barbela LAKPMRDSVSDPTPVTRYSCPYVGCKRNKEHRKFQPLKTILCVKNHYKRSHCDKRYTCSR 360

TaSTOP1A_Anahuac LAKPMRDSVSDPTPVTRYSCPYVGCKRNKEHRKFQPLKTILCVKNHYKRSHCDKRYTCSR 360

TaSTOP1B_Barbela LAKPMRDSGSDPTPVTRYSCPYVGCKRNKEHRKFQPLKTILCVKNHYKRSHCDKRYTCSR 360

TaSTOP1B_Anahuac LAKPMRDSGSDPTPVTRYSCPYVGCKRNKEHRKFQPLKTILCVKNHYKRSHCDKRYTCSR 360

TaSTOP1D_Barbela LAKPMRDPGSDPTPVTRYSCPYVGCKRNKEHRKFQPLKTILCVKNHYKRSHCDKRYTCSR 360

TaSTOP1D_Anahuac LAKPMRDSGSDPTPVTRYSCPYVGCKRNKEHRKFQPLKTILCVKNHYKRSHCDKRYTCSR 360

*******. ***************************************************

TaSTOP1A_Barbela CNTKKFSVIADLKTHEKHCGRDKWLCSCGTTFSRKDKLFGHVALFQGHTPALPMDDIKAT 420

TaSTOP1A_Anahuac CNTKKFSVIADLKTHEKHCGRDKWLCSCGTTFSRKDKLFGHVALFQGHTPALPMDDIKAT 420

TaSTOP1B_Barbela CNTKKFSVIADLKTHEKHCGRDKWLCSCGTTFSRKNKLFGHVALFQGHTPALPMDDIKAT 420

TaSTOP1B_Anahuac CNTKKFSVIADLKTHEKHCGRDKWLCSCGTTFSRKDKLFGHVALFQGHTPALPMDDIKAT 420

TaSTOP1D_Barbela CNTKKFSVIADLKTHEKHCGRDKWLCSCGTTFSRKDKLFGHVALFQGHTPALPMDDIKAS 420

TaSTOP1D_Anahuac CNTKKFSVIADLKTHEKHCGRDKWLCSCGTTFSRKDKLFGHVALFQGHTPALPMDDIKAS 420

***********************************:***********************:

TaSTOP1A_Barbela GASEQ--RSEAMDDMVGSTGYNFPGSTSDGIPNLDMKVADDTRGYFSPLNFDPCFGALDD 478

TaSTOP1A_Anahuac GASEQ--RSEAMDDMVGSTGYNFPGSTSDGIPNLDMKVADDTRGYFSPLNFDPCFGALDD 478

TaSTOP1B_Barbela GASEQPQGSEAMDDMVGSTGYNFPGSTSDGIPNLDMKVADDTRGYFSPLNFDPCFGALDD 480

TaSTOP1B_Anahuac GASEQPQGSEAMDDMVGSTGYNFPGSTSDGIPNLDMKVADDTRGYFSPLNFDPCFGALDD 480

TaSTOP1D_Barbela GALEQPQGSEAMDDMVASTGYNFPGSTSDGIPNLDMKVADDTRGYFSPLNFDPCFGALDD 480

TaSTOP1D_Anahuac GALEQPQGSEAMDDMVASTGYNFPGSTSDGIPNLDMKVADDTRGYFSPLNFDPCFGALDD 480

** ** ********.*******************************************

TaSTOP1A_Barbela FARPGFDISENPFSFLPSGPGSCSFGQLSGDS 510

TaSTOP1A_Anahuac FARPGFDISENPFSFLPSGPGSCSFGQLSGDS 510

TaSTOP1B_Barbela FARPGFDISENPFSFLPSGPGSCSFGQLSGDS 512

TaSTOP1B_Anahuac FARPGFDISENPFSFLPSGPGSCSFGQLSGDS 512

TaSTOP1D_Barbela FARPGFDISENPFSFLPSGPGSCSFGQLSGDS 512

TaSTOP1D_Anahuac FARPGFDISENPFSFLPSGPGSCSFGQLSGDS 512

********************************

**Additional File 2**B: Multiple alignments of proteins of TaSTOP1 homoeologues genes in two different bread wheat genotypes. Positions that are conserved in *TaSTOP1* homoeologues genes of both genotypes are indicated by asterisks (*).

Barbela 7/72/92_TaSTOP1B CCCCAACCCCTCCACCTCCCACGCGCGTCGGCCGGCCGGCCACCGCCGTCCCTGTTCTTC

VilosoMole_TaSTOP1B ------------------------------------------------------------

Anahuac_TaSTOP1B CCCCAACCCCTCTACCTCCCACGCGCGTCGGCCGGCCGGCCACCGCCGTCCCTGTTCTTC

Saloio_TaSTOP1B ------------------------------------------------------------

Chinesespring_TaSTOP1B ------------------------------------------------------------

Barbela 7/72/92_TaSTOP1B CTCACCGGGTCCCCGCCGTTTCCACTCCGTCCGGCAATCATTGGCCATTGAGGGTTTGAT

VilosoMole_TaSTOP1B ------------------------------------------------------------

Anahuac_TaSTOP1B CTCACCGGGTCCCCGCCGTTTCCACTCCGTCCGGCAATCATTGGCCATTGAGGGTTTGAT

Saloio_TaSTOP1B ------------------------------------------------------------

Chinesespring_TaSTOP1B ------------------------------------------------------------

Barbela 7/72/92_TaSTOP1B GCTATTCCTGTCTTATTGTCAATTTCTCATTTGGGGAGGATCGTGTGAGATAGAAGAGAG

VilosoMole_TaSTOP1B ------------------------------------------------------------

Anahuac_TaSTOP1B GCTATTCCTGTCTTATTGTCAATTTCTCATTTGGGGAGGATCGTGTGAGATAGAAGAGAG

Saloio_TaSTOP1B ------------------------------------------------------------

Chinesespring_TaSTOP1B ------------------------------------------------------------

Barbela 7/72/92_TaSTOP1B GGCGGACTGGATTGTAATCTGAGCTCTGGTGGATCTAGACTGAAAGTTGCATGAGAAAAA

VilosoMole_TaSTOP1B ------------------------------------------------------------

Anahuac_TaSTOP1B GGCGGACTGGATTGTAATCTGAGCTCTGGTGGATCTAGACTGAAAGTTGCATGAGAAAAA

Saloio_TaSTOP1B ------------------------------------------------------------

Chinesespring_TaSTOP1B ------------------------------------------------------------

Barbela 7/72/92_TaSTOP1B GTTCAGAAATTTCCATGAAAGCTTCGTCGTCGATGGCAAGCGACGCGTCAGGGAACACTG

VilosoMole_TaSTOP1B --------------ATGAAAGCTTCGTCGTCGATGGCAAGCGACGCGTCAGGGAACACTG

Anahuac_TaSTOP1B GTTCAGAAATTTCCATGAAAGCTTCGTCGTCGATGGCAAGCGACGCGTCAGGGAACACTG

Saloio_TaSTOP1B --------------ATGAAAGCTTCGTCGTCGATGGCAAGCGACGCGTCAGGGAACACTG

Chinesespring_TaSTOP1B --------------ATGAAAGCTTCGTCGTCGATGGCAAGCGACGCGTCAGGGAACACTG

**********************************************

Barbela 7/72/92_TaSTOP1B AACCTGGCCAACAGGGCGTTCGTTTCAGTTCCATGGACCAGTCTTGCTTTGCAAGACCTG

VilosoMole_TaSTOP1B AACCTGGCCAACAGGGCGTTCGTTTCAGTTCCATGGACCAGTCTTGCTTTGCAAGACCTG

Anahuac_TaSTOP1B AACCTGGCCAACAGGGCGTTCGTTTCAGTTCCATGGACCAGTCTTGCTTCGCAAGACCTG

Saloio_TaSTOP1B AACCTGGCCAACAGGGCGTTCGTTTCAGTTCCATGGACCAGTCTTGCTTTGCAAGACCTG

Chinesespring_TaSTOP1B AACCTGGCCAACAGGGCGTTCGTTTCAGTTCCATGGACCAGTCTTGCTTTGCAAGACCTG

************************************************* **********

Barbela 7/72/92_TaSTOP1B GCCAGTCAATCCCTGGCTACCCCCCATTCTTTGGCCCTCAATCTTCCAACTTTTACCTTC

VilosoMole_TaSTOP1B GCCAGTCAATCCCTGGCTACCCCCCATTCTTTGGCCCTCAATCTTCCAACTTTTACCTTC

Anahuac_TaSTOP1B GCCAGTCAATCCCTGGCTACCCCCCATTCTTTGGCCCTCAATCTTCCAACTTTTACCTTC

Saloio_TaSTOP1B GCCAGTCAATCCCTGGCTACCCCCCATTCTTTGGCCCTCAATCTTCCAACTTTTACCTTC

Chinesespring_TaSTOP1B GCCAGTCAATCCCTGGCTACCCCCCATTCTTTGGCCCTCAATCTTCCAACTTTTACCTTC

************************************************************

Barbela 7/72/92_TaSTOP1B CTGATGACAGTGTGGCTAAAGCGTGTGATCCGTTTGAACCGAATCCTCCACAGAGCAATC

VilosoMole_TaSTOP1B CTGATGACAGTGTGGCTAAAGCGTGTGATCCGTTTGAACCGAATCCTCCACAGAGCAATC

Anahuac_TaSTOP1B CTGATGACAGTGTGGCTAAAGCGTGTGATCCGTTTGAACCGAATCCTCCACAGAGCAATC

Saloio_TaSTOP1B CTGATGACAGTGTGGCTAAAGCGTGTGATCCGTTTGAACCGAATCCTCCACAGAGCAATC

Chinesespring_TaSTOP1B CTGATGACAGTGTGGCTAAAGCGTGTGATCCGTTTGAACCGAATCCTCCACAGAGCAATC

************************************************************

Barbela 7/72/92_TaSTOP1B CTGTGGCAGACTGGGATCCTCAGGCCATGCTGAGCAACCTAACCTTCCTTGAGCAGAAGA

VilosoMole_TaSTOP1B CTGTGGCAGACTGGGATCCTCAGGCCATGCTGAGCAACCTAACCTTCCTTGAGCAGAAGA

Anahuac_TaSTOP1B CTGTGGCAGACTGGGATCCTCAGGCCATGCTGAGCAACCTAACCTTCCTTGAGCAGAAGA

Saloio_TaSTOP1B CTGTGGCAGACTGGGATCCTCAGGCCATGCTGAGCAACCTAACCTTCCTTGAGCAGAAGA

Chinesespring_TaSTOP1B CTGTGGCAGACTGGGATCCTCAGGCCATGCTGAGCAACCTAACCTTCCTTGAGCAGAAGA

************************************************************

Barbela 7/72/92_TaSTOP1B TCAAGCAGGTGAAAGATATCGTGCAGTCCATGGGTAACCGAGGGAGCCAAGATGTTGGTG

VilosoMole_TaSTOP1B TCAAGCAGGTGAAAGATATCGTGCAGTCCATGGGTAACCGAGGGAGCCAAGATGTTGGTG

Anahuac_TaSTOP1B TCAAGCAGGTGAAAGATATCGTGCAGTCCATGGGTAACCGAGGGAGCCAAGATGTTGGTG

Saloio_TaSTOP1B TCAAGCAGGTGAAAGATATCGTGCAGTCCATGGGTAACCGAGGGAGCCAAGATGTTGGTG

Chinesespring_TaSTOP1B TCAAGCAGGTGAAAGATATCGTGCAGTCCATGGGTAACCGAGGGAGCCAAGATGTTGGTG

************************************************************

Barbela 7/72/92_TaSTOP1B GTTCCTGCGAGCTTGCCGCAAAGCAGCAGCTCGTCACCGCTGATCTCACTTCCATCATAA

VilosoMole_TaSTOP1B GTTCCTGCGAGCTTGCCGCAAAGCAGCAGCTCGTTACCGCTGATCTCACTTCCATCATAA

Anahuac_TaSTOP1B GTTCCTGCGAGCTTGCCGCAAAGCAGCAGCTCGTCACCGCTGATCTCACTTCCATCATAA

Saloio_TaSTOP1B GTTCCTGCGAGCTTGCCGCAAAGCAGCAGCTCGTCACCGCTGATCTCACTTCCATCATAA

Chinesespring_TaSTOP1B GTTCCTGCGAGCTTGCCGCAAAGCAGCAGCTCGTCACCGCTGATCTCACTTCCATCATAA

********************************** *************************

Barbela 7/72/92_TaSTOP1B TTCAGCTCATCTCGACTGCCGGCTCCATGCTTCCTTCCATGAAGACCCCGCTCCTTAGCA

VilosoMole_TaSTOP1B TTCAGCTCATCTCGACTGCCGGCTCCATGCTTCCTTCCATGAAGACCCCGCTCCTTAGCA

Anahuac_TaSTOP1B TTCAGCTCATCTCGACTGCCGGCTCCATGCTTCCTTCCATGAAGACCCCGCTCCTTAGCA

Saloio_TaSTOP1B TTCAGCTCATCTCGACTGCCGGCTCCATGCTTCCTTCCATGAAGACCCCGCTCCTTAGCA

Chinesespring_TaSTOP1B TTCAGCTCATCTCGACTGCCGGCTCCATGCTTCCTTCCATGAAGACCCCGCTCCTTAGCA

************************************************************

Barbela 7/72/92_TaSTOP1B GCAATCCAGCGGTCAGGCAACTCAACACGCCTGGTTCTCCCATGGGCTTTGGCTCGATTG

VilosoMole_TaSTOP1B GCAATCCAGCGGTCAGGCAACTCAACACGCCTGGTTCTCCCATGGGCTTTGGCTCGATTG

Anahuac_TaSTOP1B GCAATCCAGCGGTCAGGCAACTCAACACGCCTGGTTCTCCCATGGGCTTTGGCTCGATTG

Saloio_TaSTOP1B GCAATCCAGCGGTCAGGCAACTCAACACGCCTGGTTCTCCCATGGGCTTTGGCTCGATTG

Chinesespring_TaSTOP1B GCAATCCAGCGGTCAGGCAACTCAACACGCCTGGTTCTCCCATGGGCTTTGGCTCGATTG

************************************************************

Barbela 7/72/92_TaSTOP1B TGAATCAGCGGCCAAGCACAGTCAGGGAGGAGATGGTTCCTGACATTAGCAAGACCTCTG

VilosoMole_TaSTOP1B TGAATCAGCGGCCAAGCACAGTCAGGGAGGAGATGGTTCCTGACATTAGCAAGACCTCTG

Anahuac_TaSTOP1B TGAATCAGCGGCCAAGCACAGTCAGGGAGGAGATGGTTCCTGACATTAGCAAGACCTCTG

Saloio_TaSTOP1B TGAATCAGCGGCCAAGCACAGTCAGGGAGGAGATGGTTCCTGACATTAGCAAGACCTCTG

Chinesespring_TaSTOP1B TGAATCAGCGGCCAAGCACAGTCAGGGAGGAGATGGTTCCTGACATTAGCAAGACCTCTG

************************************************************

Barbela 7/72/92_TaSTOP1B ATTATGAGGAGCTGATGAATACCCTTAACACAGCGCATGATGAAAAGGATGATCTGATCA

VilosoMole_TaSTOP1B ATTATGAGGAGCTGATGAATACCCTTAACACAGCGCATGATGAAAAGGATGATCTGATCA

Anahuac_TaSTOP1B ATTATGAGGAGCTGATGAATACCCTTAACACAGCGCATGATGAAAAGGATGATCTGATCA

Saloio_TaSTOP1B ATTATGAGGAGCTGATGAATACCCTTAACACAGCGCATGATGAAAAGGATGATCTGATCA

Chinesespring_TaSTOP1B ATTATGAGGAGCTGATGAATACCCTTAACACAGCGCATGATGAAAAGGATGATCTGATCA

************************************************************

Barbela 7/72/92_TaSTOP1B AATGCCCAAATCCTTGTGTCGGGGAAGGGCCTGAGCCGGTTCCGATGGAAGACCATGACG

VilosoMole_TaSTOP1B AATGCCCAAATCCTTGTGTCGGGGAAGGGCCTGAGCCGGTTCCGATGGAAGACCATGATG

Anahuac_TaSTOP1B AATGCCCAAATCCTTGTGTCGGGGAAGGGCCTGAGCCGGTTCCGATGGAAGACCATGACG

Saloio_TaSTOP1B AATGCCCAAATCCTTGTGTCGGGGAAGGGCCTGAGCCGGTTCCGATGGAAGACCATGACG

Chinesespring_TaSTOP1B ACTGCCCAAATCCTTGTGTCGGGGAAGGGCCTGAGCCGGTTCCGATGGAAGACCATGACG

* ******************************************************** *

Barbela 7/72/92_TaSTOP1B TGAAGGAGAGCGATGATGGTGGTGAGGCAGAGCACCTCCCCCCTGGTTCTTATGTGGTCT

VilosoMole_TaSTOP1B TGAAGGAGAGCGATGATGGTGGTGAGGCAGAGCACCTCCCCCCTGGTTCTTATGTGGTCT

Anahuac_TaSTOP1B TGAAGGAGAGCGATGATGGTGGTGAGGCAGAGCACCTCCCCCCTGGTTCTTATGTGGTCT

Saloio_TaSTOP1B TGAAGGAGAGCGATGATGGTGGTGAGGCAGAGCACCTCCCCCCTGGTTCTTATGTGGTCT

Chinesespring_TaSTOP1B TGAAGGAGAGCGATGATGGTGGTGAGGCAGAGCACCTCCCCCCTGGTTCTTATGTGGTCT

************************************************************

Barbela 7/72/92_TaSTOP1B TGCAGTTGGAGAAGGAGGAAATTTTAGCACCACATACTCATTTCTGTGTGATATGTGGGA

VilosoMole_TaSTOP1B TGCAGTTGGAGAAGGAGGAAATTTTAGCACCACATACTCATTTCTGTGTGATATGTGGGA

Anahuac_TaSTOP1B TGCAGTTGGAGAAGGAGGAAATTTTAGCACCACATACTCATTTCTGTGTGATATGTGGGA

Saloio_TaSTOP1B TGCAGTTGGAGAAGGAGGAAATTTTAGCACCACATACTCATTTCTGTGTGATATGTGGGA

Chinesespring_TaSTOP1B TGCAGTTGGAGAAGGAGGAAATTTTAGCACCACATACTCATTTCTGTGTGATATGTGGGA

************************************************************

Barbela 7/72/92_TaSTOP1B AGGGTTTCAAGAGGGATGCTAACCTAAGGATGCACATGAGGGGCCATGGAGACGAGTACA

VilosoMole_TaSTOP1B AGGGTTTCAAGAGGGATGCTAACCTAAGGATGCACATGAGGGGCCATGGAGACGAGTACA

Anahuac_TaSTOP1B AGGGTTTCAAGAGGGATGCTAACCTAAGGATGCACATGAGGGGCCATGGAGACGAGTACA

Saloio_TaSTOP1B AGGGTTTCAAGAGGGATGCTAACCTAAGGATGCACATGAGGGGCCATGGAGACGAGTACA

Chinesespring_TaSTOP1B AGGGTTTCAAGAGGGATGCTAACCTAAGGATGCACATGAGGGGCCATGGAGACGAGTACA

************************************************************

Barbela 7/72/92_TaSTOP1B AGACTCCCGCAGCTCTTGCCAAACCCATGAGAGATTCTGGCTCAGATCCTACACCAGTTA

VilosoMole_TaSTOP1B AGACTCCCGCAGCTCTTGCCAAACCCATGAGAGATTCTGGCTCAGATCCTACACCAGTTA

Anahuac_TaSTOP1B AGACTCCCGCAGCTCTTGCCAAACCCATGAGAGATTCTGGCTCAGATCCTACACCAGTTA

Saloio_TaSTOP1B AGACTCCCGCAGCTCTTGCCAAACCCATGAGAGATTCTGGCTCAGATCCTACACCAGTTA

Chinesespring_TaSTOP1B AGACTCCCGCAGCTCTTGCCAAACCCATGAGAGATTCTGGCTCAGATCCTACACCAGTTA

************************************************************

Barbela 7/72/92_TaSTOP1B CAAGGTACTCGTGCCCATATGTCGGTTGCAAGCGGAACAAAGAGCACAGGAAGTTCCAGC

VilosoMole_TaSTOP1B CAAGGTACTCGTGCCCATATGTCGGTTGCAAGCGGAACAAAGAGCACAGGAAGTTCCAGC

Anahuac_TaSTOP1B CAAGGTACTCGTGCCCATATGTCGGTTGCAAGCGGAACAAAGAGCACAGGAAGTTCCAGC

Saloio_TaSTOP1B CAAGGTACTCGTGCCCATATGTCGGTTGCAAGCGGAACAAAGAGCACAGGAAGTTCCAGC

Chinesespring_TaSTOP1B CAAGGTACTCGTGCCCATATGTCGGTTGCAAGCGGAACAAAGAGCACAGGAAGTTCCAGC

************************************************************

Barbela 7/72/92_TaSTOP1B CCCTCAAGACAATCTTGTGTGTGAAGAACCACTACAAGAGAAGCCACTGTGACAAGAGGT

VilosoMole_TaSTOP1B CCCTCAAGACAATCTTGTGTGTGAAGAACCACTACAAGAGAAGCCACTGTGACAAGAGGT

Anahuac_TaSTOP1B CCCTCAAGACAATCTTGTGTGTGAAGAACCACTACAAGAGAAGCCACTGTGACAAGAGGT

Saloio_TaSTOP1B CCCTCAAGACAATCTTGTGTGTGAAGAACCACTACAAGAGAAGCCACTGTGACAAGAGGT

Chinesespring_TaSTOP1B CCCTCAAGACAATCTTGTGTGTGAAGAACCACTACAAGAGAAGCCACTGTGACAAGAGGT

************************************************************

Barbela 7/72/92_TaSTOP1B ATACCTGCAGCCGATGCAATACCAAGAAGTTCTCAGTCATTGCGGACTTGAAGACTCATG

VilosoMole_TaSTOP1B ATACCTGCAGCCGATGCAATACCAAGAAGTTCTCAGTCATTGCGGACTTGAAGACTCATG

Anahuac_TaSTOP1B ATACCTGCAGCCGATGCAATACCAAGAAGTTCTCAGTCATTGCGGACTTGAAGACTCATG

Saloio_TaSTOP1B ATACCTGCAGCCGATGCAATACCAAGAAGTTCTCAGTCATTGCGGACTTGAAGACTCATG

Chinesespring_TaSTOP1B ATACCTGCAGCCGATGCAATACCAAGAAGTTCTCAGTCATTGCGGACTTGAAGACTCATG

************************************************************

Barbela 7/72/92_TaSTOP1B AGAAGCACTGCGGGCGTGACAAGTGGCTCTGCTCATGTGGAACAACTTTCTCAAGAAAGA

VilosoMole_TaSTOP1B AGAAGCACTGCGGGCGTGACAAGTGGCTCTGCTCATGTGGAACAACTTTCTCAAGAAAGA

Anahuac_TaSTOP1B AGAAGCACTGCGGGCGTGACAAGTGGCTCTGCTCATGTGGAACAACTTTCTCAAGAAAGG

Saloio_TaSTOP1B AGAAGCACTGCGGGCGTGACAAGTGGCTCTGCTCATGTGGAACAACTTTCTCAAGAAAGG

Chinesespring_TaSTOP1B AGAAGCACTGCGGGCGTGACAAGTGGCTCTGCTCATGTGGAACAACTTTCTCAAGAGAGG

******************************************************** **

Barbela 7/72/92_TaSTOP1B ACAAGCTGTTCGGCCATGTCGCGCTTTTCCAAGGGCACACACCTGCTCTTCCAATGGATG

VilosoMole_TaSTOP1B ACAAGCTGTTCGGCCATGTCGCGCTTTTCCAAGGGCACACACCTGCTCTTCCAATGGATG

Anahuac_TaSTOP1B ACAAGCTGTTCGGCCATGTCGCGCTTTTCCAAGGGCACACACCTGCTCTTCCAATGGATG

Saloio_TaSTOP1B ACAAGCTGTTCGGCCATGTCGCGCTTTTCCAAGGGCACACACCTGCTCTTCCAATGGATG

Chinesespring_TaSTOP1B ACAAGCTGTTCGGCCATGTCGCGCTTTTCCAAGGGCACACACCTGCTCTAGCAATGGATG

************************************************* *********

Barbela 7/72/92_TaSTOP1B ATATTAAAGCAACAGGAGCATCGGAGCAGCCTCAGGGGAGCGAGGCGATGGACGACATGG

VilosoMole_TaSTOP1B ATATTAAAGCAACAGGAGCATCGGAGCAGCCTCAGGGGAGCGAGGCGATGGACGACATGG

Anahuac_TaSTOP1B ATATTAAAGCAACAGGAGCATCGGAGCAGCCTCAGGGGAGCGAGGCGATGGACGACATGG

Saloio_TaSTOP1B ATATTAAAGCAACAGGAGCATCGGAGCAGCCTCAGGGGAGCGAGGCGATGGGCGACATGG

Chinesespring_TaSTOP1B ATATTAAAGCAACAGGAGCATCGGAGCAGCCTCAGGGGAGCGAGGCGATGGACGACATGG

*************************************************** ********

Barbela 7/72/92_TaSTOP1B TGGGGAGCACAGGGTATAACTTCCCAGGCAGCACGTCTGATGGTATTCCGAATCTAGACA

VilosoMole_TaSTOP1B TGGGGAGCACAGGGTATAACTTCCCAGGCAGCACGTCTGATGGTATTCCGAATCTAGACA

Anahuac_TaSTOP1B TGGGGAGCACAGGGTATAACTTCCCAGGCAGCACGTCTGATGGTATTCCGAATCTAGACA

Saloio_TaSTOP1B TGGGGAGCACAGGGTATAACTTCCCAGGCAGCACGTCTGATGGTATTCCGAATCTAGACA

Chinesespring_TaSTOP1B TGGGGAGCACAGGGTATAACTTCCCAGGCAGCACGTCTGATGGTATTCCGAATCTAGACA

************************************************************

Barbela 7/72/92_TaSTOP1B TGAAAGTTGCCGATGACACACGTGGTTATTTCTCGCCCTTGAACTTCGACCCGTGCTTCG

VilosoMole_TaSTOP1B TGAAAGTTGCCGATGACACACGTGGTTATTTCTCGCCCTTGAACTTCGACCCGTGCTTCG

Anahuac_TaSTOP1B TGAAAGTTGCCGATGACACACGTGGTTATTTCTCGCCCTTGAACTTCGACCCGTGCTTCG

Saloio_TaSTOP1B TGAAAGTTGCCGATGACACACGTGGTTATTTCTCGCCCTTGAACTTCGACCCGTGCTTCG

Chinesespring_TaSTOP1B TGAAAGTTGCCGATGACACACGTGGTTATTTCTCGCCCTTGAACTTCGACCCGTGCTTCG

************************************************************

Barbela 7/72/92_TaSTOP1B GCGCCCTCGATGACTTCGCCCGCCCTGGATTCGACATCTCCGAGAACCCCTTCTCCTTCC

VilosoMole_TaSTOP1B GCGCCCTCGATGACTTCGCCCGCCCTGGATTCGACATCTCCGAGAACCCCTTCTCCTTCC

Anahuac_TaSTOP1B GCGCCCTCGATGACTTCGCCCGCCCTGGATTCGACATCTCCGAGAACCCCTTCTCCTTCC

Saloio_TaSTOP1B GCGCCCTTGATGACTTCGCCCGCCCTGGATTCGACATCTCCGAGAACCCCTTCTCCTTCC

Chinesespring_TaSTOP1B GCGCCCTCGATGACTTCGCCCCCCCTGGATTCGACATCTCCGAGAACCCCTTCTCCTTCC

******* ************* **************************************

Barbela 7/72/92_TaSTOP1B TGCCTTCGGGACCGGGTTCCTGCAGCTTTGGGCAGCTTAGTGGAGACAGCTGATGGAGAT

VilosoMole_TaSTOP1B TGCCTTCGGGACCGGGTTCCTGCAGCTTTGGGCAGCTTAGTGGAGACAGCTGATGGAGAT

Anahuac_TaSTOP1B TGCCTTCGGGACCGGGTTCCTGCAGCTTTGGGCAGCTTAGTGGAGACAGCTGATGGAGAT

Saloio_TaSTOP1B TGCCTTCGGGACCGGGTTCCTGCAGCTTTGGGCAGCTTAGTGGAGACAGCTGATGGAGAT

Chinesespring_TaSTOP1B TGCCTTCGGGACCGGGTTCCTGCAGCTTTGGGCAGCTTAGTGGAGACAGCTGATGGAGAT

************************************************************

Barbela 7/72/92_TaSTOP1B CGTCACCGTAGACAACCATGATGGTGTCGATCGTTATGTATGAATGTTATATGTATGATC

VilosoMole_TaSTOP1B CGTCACCGTAGACAACCATGATGGTGTCGATCGTTATGTATGAATGTTATATGTATGATC

Anahuac_TaSTOP1B CGTCACCGTAGACAACCATGATGGTGTCGATCGTTATGTATGAATGTTATATGTATGATC

Saloio_TaSTOP1B CGTCACCGTAGACAACCATGATGGTGTCGATCGTTATGTATGAATGTTATATGTATGATC

Chinesespring_TaSTOP1B CGTCACCGTAGACAACCATGATGGTGTCGATCGTTATGTATGAATGTTATATGTATGATC

************************************************************

Barbela 7/72/92_TaSTOP1B ATCTCAGTTCTTCCTCTATTCAGGGAG-CCATTTTGGTCCATGCCTCTTTCTTAAGTGTA

VilosoMole_TaSTOP1B ATCTCAGTTCTTCCTCTATTCAGGGAG-CCATTTTGG-----------------------

Anahuac_TaSTOP1B ATCTCAGTTCTTCCTCTATTCAGGGAG-CCATTTTGGTCCATGCCTCTTTCTTAAGTGTA

Saloio_TaSTOP1B ATCTCAGTTCTTCCTCTATTCAGGGAG-CCATTTTGG-----------------------

Chinesespring_TaSTOP1B ATCTCAGTTCTTCCTCTATTCAGGGAAATCACTAGTG-----------------------

************************** ** * *

Barbela 7/72/92_TaSTOP1B CTTTTAGTTTGTGGTCATCAATAATGATCTTACATATAGATTTGAAAG

VilosoMole_TaSTOP1B ------------------------------------------------

Anahuac_TaSTOP1B CTTTTAGTTTGTGGTCATCAATAATGATCTTACATATAGATTTGAAAG

Saloio_TaSTOP1B ------------------------------------------------

Chinesespring_TaSTOP1B ------------------------------------------------

**Additional File 2**C: Multiple alignments of nucleotide sequences of homoeologue TaSTOP1-B in selected bread wheat genotypes. Positions that are conserved in all genotypes are indicated by asterisks (*). Blue letters show the zinc finger domain (ZF4) whereas highlighted letters with colour explain SNPs among different genotypes.
