# Supplementary material for: Molecular characterization of TaSTOP1 homoeologues and their response to aluminium and proton (H+) toxicity in bread wheat (Triticum aestivum L.)
Source: BMC Plant Biol. 2013 Sep 13;13:134. doi: 10.1186/1471-2229-13-134 (PMC3848728; doi:10.1186/1471-2229-13-134)
Supplement: Additional file 3 — Detail of primers used in present investigation. [file 1471-2229-13-134-S3.rtf]

Additional File 3. Detail of primers used in present investigation
Assay	Primer name	Forward Sequence (5'-3')	Reverse Sequence (5'-3')	
EST amplification	TaSTOP_EST	TGAGGGTTTGATGCTTTTCC	CAAGGAAGGTTAGGTTGCTCA	
5'UTR RACE	TaSTOP1-5UTR	-	CAAGGAAGGTTAGGTTGCTCAGCATGG	
3'UTR RACE	TaSTOP1-3UTR	GACATCTCCGAGAACCCCTTCTCCTTC	-	
5' and 3' UTR RACE	UNIVERSAL PRIMER*	CTAATACGACTCACTATAGGGCAAGCAGTGGTATCAACGCAGAGT		
Chromosome Mapping	TaSTOP1_A	GCAGAGGAGCGAGGCGATGGACGAC	GCTGCAAGAACCCGGTCC TGAAG	
	TaSTOP1_B	CAAATGCCCAAATCCTTGTGTC	CCCCTGAGGCTGCTCCG	
	TaSTOP1_D	ATGATATCAAAGCATCAGGAGCATT	TCAACGGTGACGAGCTCCATT	
Chromosomal Arm mapping	TaSTOP1_ditA	GAATACCCTTAATCCAGCCATGAT	CCATCGCCTCGCTCCTCTGC	
	TaSTOP1_ditB	CAAATGCCCAAATCCTTGTGTC	CCCCTGAGGCTGCTCCG 	
	TaSTOP1_ditD	CCTTAACACAGCCCATGATG	CTGCTCCAATGCTCCTGATGCTTTG	
Transactivation assay	TaSTOP1_TA	AAAGCTTCGTCGTCGATGG	ggtaccTCAGCTGTCTCCACTAAG	
Expression studies (Realtime PCR)	qTaSTOP1_expA	GAAAGGACAAGCTGTTCGGC	CATCGCCTCGCTCCTCTG	
	qTaSTOP1_ expB	CTTCGGGACCGGGTTCC	CCCTGAATAGAGGAAGAACTGAGATGA	
	qTaSTOP1_expD	TCAGGAGCATTGGAGCAGCCTC	CCTGGGAAGTTATACCCTGTGCTCG	
	18SRNA	TCCACGAGGAATGCCTAGTAAGC	ACAAAGGGCAGGACGTAGTC	

*From SMARTer™ RACE cDNA Amplification Kit (Clonetech, USA)
