# Supplementary material for: Molecular characterization of TaSTOP1 homoeologues and their response to aluminium and proton (H+) toxicity in bread wheat (Triticum aestivum L.)
Source: BMC Plant Biol. 2013 Sep 13;13:134. doi: 10.1186/1471-2229-13-134 (PMC3848728; doi:10.1186/1471-2229-13-134)
Supplement: Additional file 4 — Alignment of homoeologues of TaSTOP1 5UTR region. [file 1471-2229-13-134-S4.rtf]

5UTR_TaSTOP1A_Barbela      CCCCAATCCCCTCCACCACCCACGCGCGTGGGCCGGCCGGCCACCGCCGC
5UTR_TaSTOP1A_Anahuac      CCCCAATCCCCTCCACCACCCACGCGCGTGGGCCGGCCGGCCACCGCCGC
5UTR_TaSTOP1D_Barbela      CCCCAATCCCCTCCACCTCCCACGCGCGT----CGGCCGGCCACCGCCGT
5UTR_TaSTOP1D_Anahuac      CCCCAATCCCCTCCACCTCCCACGCGCGT----CGGCCGGCCACCGCCGT
5UTR_TaSTOP1B_Barbela      -CCCCAACCCCTCCACCTCCCACGCGCGTCGGCCGGCCGGCCACCGCCGT
5UTR_TaSTOP1B_Anahuac      -CCCCAACCCCTCTACCTCCCACGCGCGTCGGCCGGCCGGCCACCGCCGT
                            ***.*:****** ***:***********    **************** 

5UTR_TaSTOP1A_Barbela      CCCTGCTCTTCCTCGCCGGGTCCCCGCCGTTTCCACTCCGTCCGGCAATC
5UTR_TaSTOP1A_Anahuac      CCCTGCTCTTCCTCGCCGGGTCCCCGCCGTTTCCACTCCGTCCGGCAATC
5UTR_TaSTOP1D_Barbela      CCCTGTTCTTCCTCGCCGGGTCCCCGCCGTTTCCACTCCGTCCGGCAATC
5UTR_TaSTOP1D_Anahuac      CCCTGTTCTTCCTCGCCGGGTCCCCGCCGTTTCCACTCCGTCCGGCAATC
5UTR_TaSTOP1B_Barbela      CCCTGTTCTTCCTCACCGGGTCCCCGCCGTTTCCACTCCGTCCGGCAATC
5UTR_TaSTOP1B_Anahuac      CCCTGTTCTTCCTCACCGGGTCCCCGCCGTTTCCACTCCGTCCGGCAATC
                           ***** ********.***********************************

5UTR_TaSTOP1A_Barbela      ATTGGCCATTGAGGGTTTGATGCTTTTCCTGTCCTATTATCAATTTCTCG
5UTR_TaSTOP1A_Anahuac      ATTGGCCATTGAGGGTTTGATGCTTTTCCTGTCCTATTATCAATTTCTCG
5UTR_TaSTOP1D_Barbela      ATTGGCCATTGAGGGTTTGATGCTTTTCCTGTCTTATTATCAATTTCTCA
5UTR_TaSTOP1D_Anahuac      ATTGGCCATTGAGGGTTTGATGCTTTTCCTGTCTTATTATCAATTTCTCA
5UTR_TaSTOP1B_Barbela      ATTGGCCATTGAGGGTTTGATGCTATTCCTGTCTTATTGTCAATTTCTCA
5UTR_TaSTOP1B_Anahuac      ATTGGCCATTGAGGGTTTGATGCTATTCCTGTCTTATTGTCAATTTCTCA
                           ************************:******** ****.**********.

5UTR_TaSTOP1A_Barbela      TTTGGGGAAGATCGTGCGAGATAGAAGAAAGGGCGGACTGGATTGTAATC
5UTR_TaSTOP1A_Anahuac      TTTGGGGAAGATCGTGCGAGATAGAAGAAAGGGCGGACTGGATTGTAATC
5UTR_TaSTOP1D_Barbela      TTTGGGGAAGATCGTGTGAGATAGAAGAGAGGGCGGACTGGATTGTAATC
5UTR_TaSTOP1D_Anahuac      TTTGGGGAAGATCGTGTGAGATAGAAGAGAGGGCGGACTGGATTGTAATC
5UTR_TaSTOP1B_Barbela      TTTGGGGAGGATCGTGTGAGATAGAAGAGAGGGCGGACTGGATTGTAATC
5UTR_TaSTOP1B_Anahuac      TTTGGGGAGGATCGTGTGAGATAGAAGAGAGGGCGGACTGGATTGTAATC
                           ********.******* ***********.*********************

5UTR_TaSTOP1A_Barbela      TGAGCTCTGGTGGATCTAGACTGGAAGTTGCATGAGAAAAAGTTCAGAAA
5UTR_TaSTOP1A_Anahuac      TGAGCTCTGGTGGATCTAGACTGGAAGTTGCATGAGAAAAAGTTCAGAAA
5UTR_TaSTOP1D_Barbela      TGAGGTCTAGTGGATCTAAACTGAAAGTTGCATGAGAAAAAGTTCAGAAA
5UTR_TaSTOP1D_Anahuac      TGAGGTCTAGTGGATCTAAACTGAAAGTTGCATGAGAAAAAGTTCAGAAA
5UTR_TaSTOP1B_Barbela      TGAGCTCTGGTGGATCTAGACTGAAAGTTGCATGAGAAAAAGTTCAGAAA
5UTR_TaSTOP1B_Anahuac      TGAGCTCTGGTGGATCTAGACTGAAAGTTGCATGAGAAAAAGTTCAGAAA
                           **** ***.*********.****.**************************

5UTR_TaSTOP1A_Barbela      TTTCCATGAAAGCTTCGTCGTCG
5UTR_TaSTOP1A_Anahuac      TTTCCATGAAAGCTTCGTCGTCG
5UTR_TaSTOP1D_Barbela      TTTCCATGAAAGCTTCGTCGTCG
5UTR_TaSTOP1D_Anahuac      TTTCCATGAAAGCTTCGTCGTCG
5UTR_TaSTOP1B_Barbela      TTTCCATGAAAGCTTCGTCGTCG
5UTR_TaSTOP1B_Anahuac      TTTCCATGAAAGCTTCGTCGTCG
                           ***********************

Additional File 4: Alignment of the TaSTOP1 homoeologues genes 5' UTR regions in two bread wheat genotypes (Barbela 7/72/92 and Anahuac). The coding sequence of each gene is presented in red colour letters. The presence of 3-AF1 binding site (light responsive element) and pyrimidine-rich stretch within the 5' untranslated regions of TaSTOP1 homoeologues genes is highlighted in yellow and blue colour, respectively. Positions that are conserved in TaSTOP1 homoeologues genes of both genotypes are indicated by asterisks (*).
