# Supplementary material for: Molecular characterization of TaSTOP1 homoeologues and their response to aluminium and proton (H+) toxicity in bread wheat (Triticum aestivum L.)
Source: BMC Plant Biol. 2013 Sep 13;13:134. doi: 10.1186/1471-2229-13-134 (PMC3848728; doi:10.1186/1471-2229-13-134)
Supplement: Additional file 5 — Physiological characterization of Barbela 7/72/92 and Anahuac roots under Al stress. [file 1471-2229-13-134-S5.doc]

| **Eriochrome cyanine R assay: Root re-growth measurement** |
| --- |
| 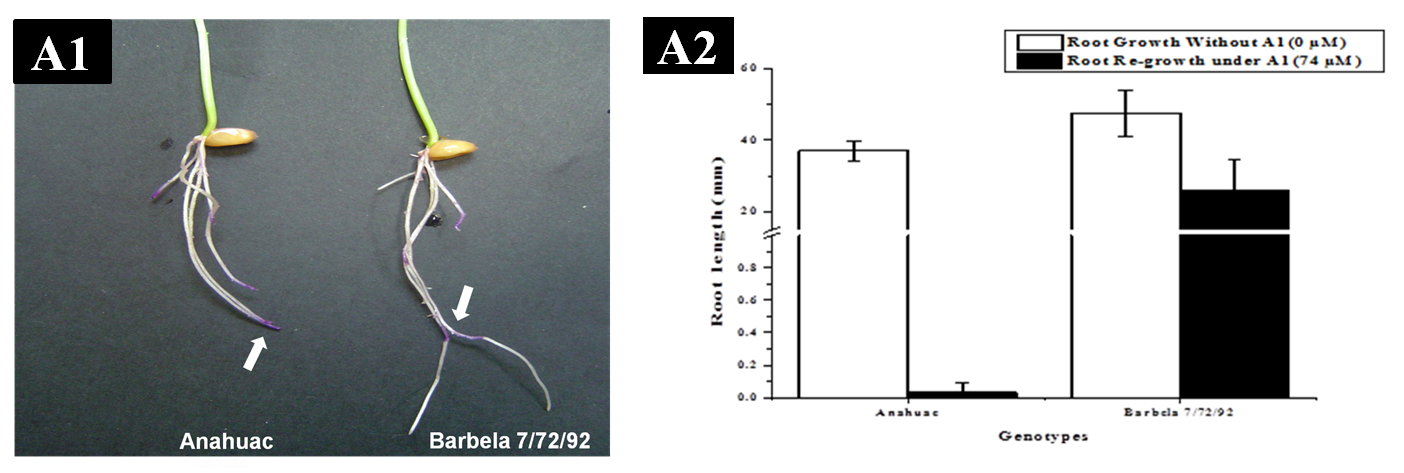 |
| **Hematoxylin assay: Visual detection of Al accumulation** |
| 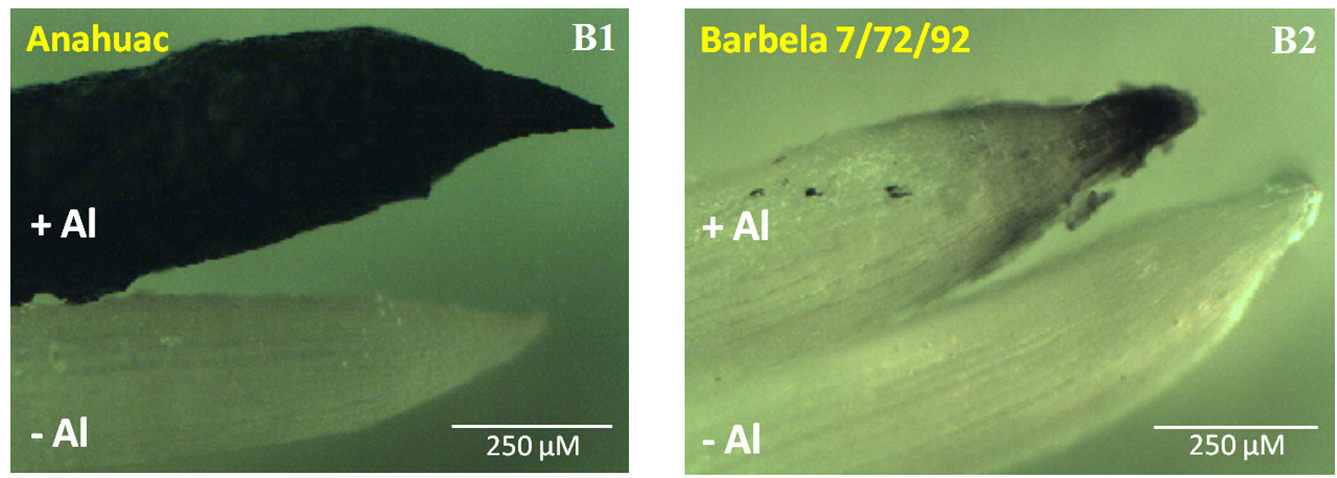 |
| Additional File 5: Physiological characterization of roots of two bread wheat genotypes Anahuac and Barbela 7/72/92 for Al resistance using eriochrome cyanine R (A) and Hematoxylin (B) assay. In picture A1, arrow indicates the eriochrome cyanine dye as a marker and the root after this arrow shows the re-growth whereas in figure A2 the root growth under low pH (pH 4.0) without Al (control) stress and root re-growth after exposure of Al (74 µM). In figure B, dark colour in roots represents the accumulation of Al [(B1, Anahuac) and (B2, Barbela 7/72/92).The upper and lower root samples in B1 and B2 were grown with and without (Control) Al. |
