# Supplementary material for: Molecular characterization of TaSTOP1 homoeologues and their response to aluminium and proton (H+) toxicity in bread wheat (Triticum aestivum L.)
Source: BMC Plant Biol. 2013 Sep 13;13:134. doi: 10.1186/1471-2229-13-134 (PMC3848728; doi:10.1186/1471-2229-13-134)
Supplement: Additional file 6 — Nucleotide and protein sequence of TaSTOP1 in different bread wheat genotypes. [file 1471-2229-13-134-S6.rtf]

Additional File: 6. Nucleotide sequence TaSTOP1 homoeologues gene in different bread wheat genotypes
Note: Highlighted region is ORF
>COMPLETE_SEQ_TaSTOP1_BARBELA 7/72/92 _Genome A
CCCCAATCCCCTCCACCACCCACGCGCGTGGGCCGGCCGGCCACCGCCGCCCCTGCTCTTCCTCGCCGGGTCCCCGCCGTTTCCACTCCGTCCGGCAATCATTGGCCATTGAGGGTTTGATGCTTTTCCTGTCCTATTATCAATTTCTCGTTTGGGGAAGATCGTGCGAGATAGAAGAAAGGGCGGACTGGATTGTAATCTGAGCTCTGGTGGATCTAGACTGGAAGTTGCATGAGAAAAAGTTCAGAAATTTCCATGAAAGCTTCGTCGTCGATGGCAAGCGACGCTTCAGGGAACACTGACCCTGGCCAACAGGGTGCTCGTTTCAGTTCCATGGACCAGTCTTGCTTTGCAAGACCTGGCCAGTCAATCCCTGGCTACCCCCCATTCTTTGGCCCTCAGTCTTCCAACTTTTACCTTCCTGATGACAGTGTGGCTAAAGCGTGTGATCCGTTCGAGCCGAATCCTCCTCAGAACAATCCTGTGGCAGATTGGGACCCTCAGGCCATGCTGAGCAACCTAACCTTCCTTGAGCAGAAGATCAAGCAGGTGAAAGATATCGTGCAGTCCATGGGTAACCGAGGGAGCCAAGATGTTGGTGGTTCCTGCGAGCTTGCCGCAAAGCAGCAGCTCGTCACCGCTGATCTCACTTCCATCATAATTCAGCTCATCTCGACTGCCGGCTCCATGCTTCCTTCCATGAAGACCCCGCTGCTTAGCAGCAATCCAGCGGTCAGGCAGCTCAACACGCCTGGTTCTCCCATGGGCTTTGGCTCGATTGTGAATCAGCGGCCAAGCACAGTCAGGGAGGAGATGGTTCCTGACATTACCAAGACCCCTGACTATGAGGATCTGATGAATACCCTTAATCCAGCCCATGATGAAAAGGATGATCTGATCAAATGCCCAAATCCTTGTGTTGGGGAAGGGCCTGAGCCGGTTCCGATGGAAGACCATGACGTGAAGGAGAGCGATGATGGTGGCGAGGCAGAGCATCTCCCCCCTGGTTCTTATGTGGTCTTGCAATTGGAGAAGGAGGAGATTTTAGCACCACACACTCATTTCTGTGTGATATGTGGCAAGGGTTTCAAGAGGGATGCTAACCTAAGGATGCACATGAGGGGCCATGGAGACGAGTACAAAACTCCCGCAGCTCTTGCCAAACCCATGAGAGATTCTGTCTCAGATCCTACACCAGTTACAAGGTACTCGTGCCCATATGTCGGTTGCAAGCGCAACAAAGAGCACAGGAAGTTCCAGCCCCTCAAGACAATCTTGTGTGTGAAGAACCACTACAAGAGAAGCCATTGCGACAAGAGGTATACCTGCAGCCGATGCAACACCAAGAAGTTCTCAGTCATTGCGGACTTGAAGACTCATGAGAAGCACTGTGGGCGTGACAAGTGGCTCTGCTCATGTGGAACAACTTTCTCAAGAAAGGACAAGCTGTTCGGCCATGTCGCGCTTTTCCAAGGGCACACACCTGCTCTTCCAATGGATGATATTAAAGCAACAGGAGCATCGGAGCAGAGGAGCGAGGCGATGGACGACATGGTGGGGAGCACAGGGTATAACTTCCCAGGCAGCACGTCTGATGGTATTCCGAATCTAGACATGAAAGTTGCCGATGACACACGTGGTTATTTCTCTCCCTTGAACTTCGACCCCTGCTTCGGCGCCCTCGATGACTTCGCCCGACCTGGATTCGACATCTCCGAGAACCCCTTCTCCTTCCTGCCTTCAGGACCGGGTTCTTGCAGCTTTGGGCAGCTTAGTGGAGACAGCTGATGGAGTTCGTCGCCGTAGACAACCATGATGGTGTCGATTGTTATGTATGAATGTTATATGTATGACCGTCTCAGTTCTTCCTCTATTCAGGGAGCCATTTTGGTCCATGCTTCTTTCTTAAGTGTACTTTTAGTTTGTGGTCATCAATAATGATCTTACATATAGATTTGAAAG
>COMPLETE_SEQ_TaSTOP1_BARBELA 7/72/92 _GENOME B
CCCCAACCCCTCCACCTCCCACGCGCGTCGGCCGGCCGGCCACCGCCGTCCCTGTTCTTCCTCACCGGGTCCCCGCCGTTTCCACTCCGTCCGGCAATCATTGGCCATTGAGGGTTTGATGCTATTCCTGTCTTATTGTCAATTTCTCATTTGGGGAGGATCGTGTGAGATAGAAGAGAGGGCGGACTGGATTGTAATCTGAGCTCTGGTGGATCTAGACTGAAAGTTGCATGAGAAAAAGTTCAGAAATTTCCATGAAAGCTTCGTCGTCGATGGCAAGCGACGCGTCAGGGAACACTGAACCTGGCCAACAGGGCGTTCGTTTCAGTTCCATGGACCAGTCTTGCTTTGCAAGACCTGGCCAGTCAATCCCTGGCTACCCCCCATTCTTTGGCCCTCAATCTTCCAACTTTTACCTTCCTGATGACAGTGTGGCTAAAGCGTGTGATCCGTTTGAACCGAATCCTCCACAGAGCAATCCTGTGGCAGACTGGGATCCTCAGGCCATGCTGAGCAACCTAACCTTCCTTGAGCAGAAGATCAAGCAGGTGAAAGATATCGTGCAGTCCATGGGTAACCGAGGGAGCCAAGATGTTGGTGGTTCCTGCGAGCTTGCCGCAAAGCAGCAGCTCGTCACCGCTGATCTCACTTCCATCATAATTCAGCTCATCTCGACTGCCGGCTCCATGCTTCCTTCCATGAAGACCCCGCTCCTTAGCAGCAATCCAGCGGTCAGGCAACTCAACACGCCTGGTTCTCCCATGGGCTTTGGCTCGATTGTGAATCAGCGGCCAAGCACAGTCAGGGAGGAGATGGTTCCTGACATTAGCAAGACCTCTGATTATGAGGAGCTGATGAATACCCTTAACACAGCGCATGATGAAAAGGATGATCTGATCAAATGCCCAAATCCTTGTGTCGGGGAAGGGCCTGAGCCGGTTCCGATGGAAGACCATGACGTGAAGGAGAGCGATGATGGTGGTGAGGCAGAGCACCTCCCCCCTGGTTCTTATGTGGTCTTGCAGTTGGAGAAGGAGGAAATTTTAGCACCACATACTCATTTCTGTGTGATATGTGGGAAGGGTTTCAAGAGGGATGCTAACCTAAGGATGCACATGAGGGGCCATGGAGACGAGTACAAGACTCCCGCAGCTCTTGCCAAACCCATGAGAGATTCTGGCTCAGATCCTACACCAGTTACAAGGTACTCGTGCCCATATGTCGGTTGCAAGCGGAACAAAGAGCACAGGAAGTTCCAGCCCCTCAAGACAATCTTGTGTGTGAAGAACCACTACAAGAGAAGCCACTGTGACAAGAGGTATACCTGCAGCCGATGCAATACCAAGAAGTTCTCAGTCATTGCGGACTTGAAGACTCATGAGAAGCACTGCGGGCGTGACAAGTGGCTCTGCTCATGTGGAACAACTTTCTCAAGAAAGAACAAGCTGTTCGGCCATGTCGCGCTTTTCCAAGGGCACACACCTGCTCTTCCAATGGATGATATTAAAGCAACAGGAGCATCGGAGCAGCCTCAGGGGAGCGAGGCGATGGACGACATGGTGGGGAGCACAGGGTATAACTTCCCAGGCAGCACGTCTGATGGTATTCCGAATCTAGACATGAAAGTTGCCGATGACACACGTGGTTATTTCTCGCCCTTGAACTTCGACCCGTGCTTCGGCGCCCTCGATGACTTCGCCCGCCCTGGATTCGACATCTCCGAGAACCCCTTCTCCTTCCTGCCTTCGGGACCGGGTTCCTGCAGCTTTGGGCAGCTTAGTGGAGACAGCTGATGGAGATCGTCACCGTAGACAACCATGATGGTGTCGATCGTTATGTATGAATGTTATATGTATGATCATCTCAGTTCTTCCTCTATTCAGGGAGCCATTTTGGTCCATGCCTCTTTCTTAAGTGTACTTTTAGTTTGTGGTCATCAATAATGATCTTACATATAGATTTGAAAG

>Complete_SEQ_ TaSTOP1_ BARBELA 7/72/92 _GENOME D
CCCCAATCCCCTCCACCTCCCACGCGCGTCGGCCGGCCACCGCCGTCCCTGTTCTTCCTCGCCGGGTCCCCGCCGTTTCCACTCCGTCCGGCAATCATTGGCCATTGAGGGTTTGATGCTTTTCCTGTCTTATTATCAATTTCTCATTTGGGGAAGATCGTGTGAGATAGAAGAGAGGGCGGACTGGATTGTAATCTGAGGTCTAGTGGATCTAAACTGAAAGTTGCATGAGAAAAAGTTCAGAAATTTCCATGAAAGCTTCGTCGTCGATGGCAAGCGACGCGTCAGGGAACACTGAACCTGGCCAACAGGGTGTTCGTTTCAGTTCCATGGACCAGTCTTGCTTTGCAAGACCTGGCCAGTCAATCCCTGGCTACCCCCCATTCTTTGGCCCTCAATCTTCCAACTTTTACCTTCCTGATGACAGTGTGGCTAAAGCGTGTGATCTGTTTGAACCGAATCCTCCACAGAACAATCCTGTGGCTGATTGGGATCCTCAGGCCATGCTGAGCAACCTAACCTTCCTTGAGCAGAAGATCAAGCAGGTGAAAGATATCGTGCAGTCCATGGGTAACCGAGGGAGCCAAGATGCTGGTGGTTCCTGCGAGCTTGCTGCAAAGCAGCAGCTCGTCACCGCCGATCTCACTTCCATCATAATTCAGCTCATCTCAACTGCCGGCTCCATGCTTCCTTCCATGAAGACCCCGCTCCTTAGCGGCAATCCAGCGGTCAGGCAGCTCAACACGCCTGGTTCTCCCATGGGCTTTGGCTCGATCGTGAATCAGCGGCCAAGCACAATCAGGGAGGAGGTGGTTAATGACATTAGCAAGACCTCTGACTATGAGGAGCTGATGAATACCCTTAACACAGCCCATGATGAAAAGGATGATCTGATCAAATGCCCAAATCCTTGTGTCGGGGAAGGACCTGAGCCGGTTCCGATGGAAGACCATGACGTGAAGGAGAGCGATGATGGTGGCGAGGCAGAGCACCTCCCCCCTGGTTCTTATGTGGTCTTGCAATTGGAGAAGGAGGAAATTCTAGCACCACACACTCATTTCTGTGTGATATGTGGCAAGGGTTTCAAGAGGGATGCTAACCTAAGGATGCACATGAGGGGCCATGGAGACGAGTACAAGACTCCTGCAGCTCTTGCCAAACCCATGAGAGATCCTGGCTCAGATCCTACACCAGTTACAAGGTACTCGTGCCCATATGTCGGTTGCAAGCGGAACAAAGAGCACAGGAAGTTCCAGCCTCTCAAGACAATCTTGTGTGTGAAGAACCACTACAAGAGAAGCCACTGTGACAAGAGGTATACCTGCAGCCGATGCAACACCAAGAAGTTCTCAGTCATTGCTGACTTGAAGACTCATGAGAAGCACTGCGGGCGTGACAAGTGGCTCTGCTCATGTGGAACGACCTTCTCAAGAAAAGACAAGCTGTTCGGGCATGTCGCGCTTTTCCAAGGGCACACACCTGCTCTTCCAATGGATGATATCAAAGCATCAGGAGCATTGGAGCAGCCTCAGGGGAGCGAGGCGATGGACGACATGGTGGCGAGCACAGGGTATAACTTCCCAGGCAGCACGTCTGATGGTATTCCGAATCTAGACATGAAAGTTGCCGATGACACACGTGGTTATTTCTCGCCCTTGAACTTCGACCCGTGCTTCGGTGCCCTCGATGACTTTGCTCGCCCTGGATTCGACATCTCTGAGAACCCCTTCTCCTTCCTGCCTTCAGGACCGGGGTCGTGCAGTTTTGGGCAGCTTAGTGGCGACAGCTAATGGAGCTCGTCACCGTTGACAACCATGATGGTGTCGATCGTTATGTATGAATGTTATATGTATGACCGTCTTAGTTCTTCCTCTATTCAGGGAGCCATTTTGGTCCATGCCTCTTTCTTAAGTGTACTTTTAGTTTGTGGTCATCAATAATGATCAAGTACTTACATATAGATTTGAAAG


>Complete_SEQ_Ta STOP1_ Anahuac _GENOME A
CCCCAATCCCCTCCACCACCCACGCGCGTGGGCCGGCCGGCCACCGCCGCCCCTGCTCTTCCTCGCCGGGTCCCCGCCGTTTCCACTCCGTCCGGCAATCATTGGCCATTGAGGGTTTGATGCTTTTCCTGTCCTATTATCAATTTCTCGTTTGGGGAAGATCGTGCGAGATAGAAGAAAGGGCGGACTGGATTGTAATCTGAGCTCTGGTGGATCTAGACTGGAAGTTGCATGAGAAAAAGTTCAGAAATTTCCATGAAAGCTTCGTCGTCGATGGCAAGCGACGCTTCAGGGAACACTGACCCTGGCCAACAGGGTGCTCGTTTCAGTTCCATGGACCAGTCTTGCTTTGCAAGACCTGGCCAGTCAATCCCTGGCTACCCCCCATTCTTTGGCCCTCAGTCTTCCAACTTTTACCTTCCTGATGACAGTGTGGCTAAAGCGTGTGATCCGTTCGAGCCGAATCCTCCTCAGAACAATCCTGTGGCAGATTGGGACCCTCAGGCCATGCTGAGCAACCTAACCTTCCTTGAGCAGAAGATCAAGCAGGTGAAAGATATCGTGCAGTCCATGGGTAACCGAGGGAGCCAAGATGTTGGTGGTTCCTGCGAGCTTGCCGCAAAGCAGCAGCTCGTCACCGCTGATCTCACTTCCATCATAATTCAGCTCATCTCGACTGCCGGCTCCATGCTTCCTTCCATGAAGACCCCGCTGCTTAGCAGCAATCCAGCGGTCAGGCAGCTCAACACGCCTGGTTCTCCCATGGGCTTTGGCTCGATTGTGAATCAGCGGCCAAGCACAGTCAGGGAGGAGATGGTTCCTGACATTACCAAGACCCCTGACTATGAGGATCTGATGAATACCCTTAATCCAGCCCATGATGAAAAGGATGATCTGATCAAATGCCCAAATCCTTGTGTTGGGGAAGGGCCTGAGCCGGTTCCGATGGAAGACCATGACGTGAAGGAGAGCGATGATGGTGGCGAGGCAGAGCATCTCCCCCCTGGTTCTTATGTGGTCTTGCAATTGGAGAAGGAGGAGATTTTAGCACCACACACTCATTTCTGTGTGATATGTGGCAAGGGTTTCAAGAGGGATGCTAACCTAAGGATGCACATGAGGGGCCATGGAGACGAGTACAAAACTCCCGCAGCTCTTGCCAAACCCATGAGAGATTCTGTCTCAGATCCTACACCAGTTACAAGGTACTCGTGCCCATATGTCGGTTGCAAGCGCAACAAAGAGCACAGGAAGTTCCAGCCCCTCAAGACAATCTTGTGTGTGAAGAACCACTACAAGAGAAGCCATTGCGACAAGAGGTATACCTGCAGCCGATGCAACACCAAGAAGTTCTCAGTCATTGCGGACTTGAAGACTCATGAGAAGCACTGTGGGCGTGACAAGTGGCTCTGCTCATGTGGAACAACTTTCTCAAGAAAGGACAAGCTGTTCGGCCATGTCGCGCTTTTCCAAGGGCACACACCTGCTCTTCCAATGGATGATATTAAAGCAACAGGAGCATCGGAGCAGAGGAGCGAGGCGATGGACGACATGGTGGGGAGCACAGGGTATAACTTCCCAGGCAGCACGTCTGATGGTATTCCGAATCTAGACATGAAAGTTGCCGATGACACACGTGGTTATTTCTCTCCCTTGAACTTCGACCCCTGCTTCGGCGCCCTCGATGACTTCGCCCGACCTGGATTCGACATCTCCGAGAACCCCTTCTCCTTCCTGCCTTCAGGACCGGGTTCTTGCAGCTTTGGGCAGCTTAGTGGAGACAGCTGATGGAGTTCGTCGCCGTAGACAACCATGATGGTGTCGATTGTTATGTATGAATGTTATATGTATGACCGTCTCAGTTCTTCCTCTATTCAGGGAGCCATTTTGGTCCATGCTTCTTTCTTAAGTGTACTTTTAGTTTGTGGTCATCAATAATGATCTTACATATAGATTTGAAAG
>Complete_SEQ_ TaSTOP1_ Anahuac _GENOME B
CCCCAACCCCTCTACCTCCCACGCGCGTCGGCCGGCCGGCCACCGCCGTCCCTGTTCTTCCTCACCGGGTCCCCGCCGTTTCCACTCCGTCCGGCAATCATTGGCCATTGAGGGTTTGATGCTATTCCTGTCTTATTGTCAATTTCTCATTTGGGGAGGATCGTGTGAGATAGAAGAGAGGGCGGACTGGATTGTAATCTGAGCTCTGGTGGATCTAGACTGAAAGTTGCATGAGAAAAAGTTCAGAAATTTCCATGAAAGCTTCGTCGTCGATGGCAAGCGACGCGTCAGGGAACACTGAACCTGGCCAACAGGGCGTTCGTTTCAGTTCCATGGACCAGTCTTGCTTCGCAAGACCTGGCCAGTCAATCCCTGGCTACCCCCCATTCTTTGGCCCTCAATCTTCCAACTTTTACCTTCCTGATGACAGTGTGGCTAAAGCGTGTGATCCGTTTGAACCGAATCCTCCACAGAGCAATCCTGTGGCAGACTGGGATCCTCAGGCCATGCTGAGCAACCTAACCTTCCTTGAGCAGAAGATCAAGCAGGTGAAAGATATCGTGCAGTCCATGGGTAACCGAGGGAGCCAAGATGTTGGTGGTTCCTGCGAGCTTGCCGCAAAGCAGCAGCTCGTCACCGCTGATCTCACTTCCATCATAATTCAGCTCATCTCGACTGCCGGCTCCATGCTTCCTTCCATGAAGACCCCGCTCCTTAGCAGCAATCCAGCGGTCAGGCAACTCAACACGCCTGGTTCTCCCATGGGCTTTGGCTCGATTGTGAATCAGCGGCCAAGCACAGTCAGGGAGGAGATGGTTCCTGACATTAGCAAGACCTCTGATTATGAGGAGCTGATGAATACCCTTAACACAGCGCATGATGAAAAGGATGATCTGATCAAATGCCCAAATCCTTGTGTCGGGGAAGGGCCTGAGCCGGTTCCGATGGAAGACCATGACGTGAAGGAGAGCGATGATGGTGGTGAGGCAGAGCACCTCCCCCCTGGTTCTTATGTGGTCTTGCAGTTGGAGAAGGAGGAAATTTTAGCACCACATACTCATTTCTGTGTGATATGTGGGAAGGGTTTCAAGAGGGATGCTAACCTAAGGATGCACATGAGGGGCCATGGAGACGAGTACAAGACTCCCGCAGCTCTTGCCAAACCCATGAGAGATTCTGGCTCAGATCCTACACCAGTTACAAGGTACTCGTGCCCATATGTCGGTTGCAAGCGGAACAAAGAGCACAGGAAGTTCCAGCCCCTCAAGACAATCTTGTGTGTGAAGAACCACTACAAGAGAAGCCACTGTGACAAGAGGTATACCTGCAGCCGATGCAATACCAAGAAGTTCTCAGTCATTGCGGACTTGAAGACTCATGAGAAGCACTGCGGGCGTGACAAGTGGCTCTGCTCATGTGGAACAACTTTCTCAAGAAAGGACAAGCTGTTCGGCCATGTCGCGCTTTTCCAAGGGCACACACCTGCTCTTCCAATGGATGATATTAAAGCAACAGGAGCATCGGAGCAGCCTCAGGGGAGCGAGGCGATGGACGACATGGTGGGGAGCACAGGGTATAACTTCCCAGGCAGCACGTCTGATGGTATTCCGAATCTAGACATGAAAGTTGCCGATGACACACGTGGTTATTTCTCGCCCTTGAACTTCGACCCGTGCTTCGGCGCCCTCGATGACTTCGCCCGCCCTGGATTCGACATCTCCGAGAACCCCTTCTCCTTCCTGCCTTCGGGACCGGGTTCCTGCAGCTTTGGGCAGCTTAGTGGAGACAGCTGATGGAGATCGTCACCGTAGACAACCATGATGGTGTCGATCGTTATGTATGAATGTTATATGTATGATCATCTCAGTTCTTCCTCTATTCAGGGAGCCATTTTGGTCCATGCCTCTTTCTTAAGTGTACTTTTAGTTTGTGGTCATCAATAATGATCTTACATATAGATTTGAAAG


>Complete_SEQ_Ta STOP1_ Anahuac _GENOME D
CCCCAATCCCCTCCACCTCCCACGCGCGTCGGCCGGCCACCGCCGTCCCTGTTCTTCCTCGCCGGGTCCCCGCCGTTTCCACTCCGTCCGGCAATCATTGGCCATTGAGGGTTTGATGCTTTTCCTGTCTTATTATCAATTTCTCATTTGGGGAAGATCGTGTGAGATAGAAGAGAGGGCGGACTGGATTGTAATCTGAGGTCTAGTGGATCTAAACTGAAAGTTGCATGAGAAAAAGTTCAGAAATTTCCATGAAAGCTTCGTCGTCGATGGCAAGCGACGCGTCAGGGAACACTGAACCTGGCCAACAGGGTGTTCGTTTCAGTTCCATGGACCAGTCTTGCTTTGCAAGACCTGGCCAGTCAATCCCTGGCTACCCCCCATTCTTTGGCCCTCAATCTTCCAACTTTTACCTTCCTGATGACAGTGTGGCTAAAGCGTGTGATCTGTTTGAACCGAATCCTCCACAGAACAATCCTGTGGCTGATTGGGATCCTCAGGCCATGCTGAGCAACCTAACCTTCCTTGAGCAGAAGATCAAGCAGGTGAAAGATATCGTGCAGTCCATGGGTAACCGAGGGAGCCAAGATGCTGGTGGTTCCTGCGAGCTTGCTGCAAAGCAGCAGCTCGTCACCGCCGATCTCACTTCCATCATAATTCAGCTCATCTCAACTGCCGGCTCCATGCTTCCTTCCATGAAGACCCCGCTCCTTAGCAGCAATCCAGCGGTCAGGCAGCTCAACACGCCTGGTTCTCCCATGGGCTTTGGCTCGATCGTGAATCAGCGGCCAAGCACAATCAGGGAGGAGGTGGTTAATGACATTAGCAAGACCTCTGACTATGAGGAGCTGATGAATACCCTTAACACAGCCCATGATGAAAAGGATGATCTGATCAAATGCCCAAATCCTTGTGTCGGGGAAGGACCTGAGCCGGTTCCGATGGAAGACCATGACGTGAAGGAGAGCGATGATGGTGGCGAGGCAGAGCACCTCCCCCCTGGTTCTTATGTGGTCTTGCAATTGGAGAAGGAGGAAATTCTAGCACCACACACTCATTTCTGTGTGATATGTGGCAAGGGTTTCAAGAGGGATGCTAACCTAAGGATGCACATGAGGGGCCATGGAGACGAGTACAAGACTCCCGCAGCTCTTGCCAAACCCATGAGAGATTCTGGCTCAGATCCTACACCAGTTACAAGGTACTCGTGCCCATATGTCGGTTGCAAGCGGAACAAAGAGCACAGGAAGTTCCAGCCTCTCAAGACAATCTTGTGTGTGAAGAACCACTACAAGAGAAGCCACTGTGACAAGAGGTATACCTGCAGCCGATGCAACACCAAGAAGTTCTCAGTCATTGCTGACTTGAAGACTCATGAGAAGCACTGCGGGCGTGACAAGTGGCTCTGCTCATGTGGAACGACCTTCTCAAGAAAAGACAAGCTGTTTGGGCATGTCGCGCTTTTCCAAGGGCACACACCTGCTCTTCCAATGGATGATATCAAAGCATCAGGAGCATTGGAGCAGCCTCAGGGGAGCGAGGCGATGGACGACATGGTGGCGAGCACAGGGTATAACTTCCCAGGCAGCACGTCTGATGGTATTCCGAATCTAGACATGAAAGTTGCCGATGACACACGTGGTTATTTCTCGCCCTTGAACTTCGACCCGTGCTTCGGTGCCCTCGATGACTTTGCTCGCCCTGGATTCGACATCTCCGAGAACCCCTTCTCCTTCCTGCCTTCAGGACCGGGGTCGTGCAGTTTTGGGCAGCTTAGTGGCGACAGCTAATGGAGCTCGTCACCGTTGACAACCATGATGGTGTCGATCGTTATGTATGAATGTTATATGTATGACCGTCTTAGTTCTTCCTCTATTCAGGGAGCCATTTTGGTCCATGCCTCTTTCTTAAGTGTACTTTTAGTTTGTGGTCATCAATAATGATCAAGTACTTACATATAGATTTGAAAG


>SEQ_TaSTOP1_ ChineseSpring _Genome A
ATGAAAGCTTCGTCGTCGATGGCAAGCGACGCTTCAGGGAACACTGACCCTGGCCAACAGGGTGCTCGTTTCAGTTCCATGGACCAGTCTTGCTTTGCAAGACCTGGCCAGTCAATCCCTGGCTACCCCCCATTCTTTGGTCCTCAGTCTTCCAACTTTTACCTTCCTGATGACAGTGTGGCTAAAGCGTGTGATCCGTTCGAGCCGAATCCTCCTCAGAACAATCCTGTGGCAGATTGGGACCCTCAGGCCATGCTGAGCAACCTAACCTTCCTTGAGCAGAAGATCAAGCAGGTGAAAGATATCGTGCAGTCCATGGGTAACCGAGGGAGCCAAGATGTTGGTGGTTCCTGCGAGCTTGCCGCAAAGCAGCAGCTCGTCACCGCTGATCTCACTTCCATCATAATTCAGCTCATCTCGACTGCCGGCTCCATGCTTCCTTCCATGAAGACCCCGCTGCTTAGCAGCAATCCAGCGGTCAGGCAGCTCAATACGCCTGGTTCTCCCATGGGCTTTGGCTCGATTGTGAATCAGCGGCCAAGCACAGTCAGGGAGGAGATGGTTCCTGACATTACCAAGACCCCTGACTATGAGGATCTGATGAATACCCTTAATCCAGCCCATGATGAAAAGGATGATCTGATCAAATGCCCAAATCCTTGTGTTGGGGAAGGGCCTGAGCCGGTTCCGATGGAAGACCATGACGTGAAGGAGAGCGATGATGGTGGCGAGGCAGAGCATCTCCCCCCTGGTTCTTATGTGGTCTTGCAATTGGAGAAGGAGGAGATTTTAGCACCACACACTCATTTCTGTGTGATATGTGGCAAGGGTTTCAAGAGGGATGCTAACCTAAGGATGCACATGAAGGGCCATGGAGACGAGTACAAAACTCCCGCAGCTCTTGCCAAACCCATGAGAGATTCTGTCTCAGATCCTACACCAGTTACAAGGTACTCGTGCCCATATGTCGGTTGCAAGCGCAACAAAGAGCACAGGAAGTTCCAGCCCCTCAAGACAATCTTGTGTGTGAAGAACCACTACAAGAGAAGCCATTGCGACAAGAGGTATACCTGCAGCCGATGCAACACCAAGAAGTTCTCAGTCATTGCGGACTTGAAGACTCATGAGAAGCACTGTGGGCGTGACAAGTGGCTCTGCTCATGTGGAACAACTTTCTCAAGAAAGGACAAGCTGTTCGGCCATGTCGCGCTTTTCCAAGGGCACACACCTGCTCTTCCAATGGATGATATTAAAGCAACAGGAGCATCGGAGCAGAGGAGCGAGGCGATGGACGACATGGTGGGGAGCACAGGGTATAACTTCCCAGGCAGCACGTCTGATGGTATTCCGAATCTAGACATGAAAGTTGCCGATGACACACGTGGTTATTTCTCTCCCTTGAACTTCGACCCCTGCTTCGGCGCCCTCGATGACTTCGCCCGACCTGGATTCGACATCTCCGAGAACCCCTTCTCCTTCCTGCCTTCAGGACCGGGTTCTTGCAGCTTTGGGCAGCTTAGTGGAGACAGCTGATGGAGTTCGTCGCCGTAGACAACCATGATGGTGTCGATTGTTATGTATGAATGTTATATGTATGACCGTCTCAGTTCTTCCTCTATTCAGGGAGCCATTTTGG

>SEQ_TaSTOP1 ChineseSpring _Genome B
ATGAAAGCTTCGTCGTCGATGGCAAGCGACGCGTCAGGGAACACTGAACCTGGCCAACAGGGCGTTCGTTTCAGTTCCATGGACCAGTCTTGCTTTGCAAGACCTGGCCAGTCAATCCCTGGCTACCCCCCATTCTTTGGCCCTCAATCTTCCAACTTTTACCTTCCTGATGACAGTGTGGCTAAAGCGTGTGATCCGTTTGAACCGAATCCTCCACAGAGCAATCCTGTGGCAGACTGGGATCCTCAGGCCATGCTGAGCAACCTAACCTTCCTTGAGCAGAAGATCAAGCAGGTGAAAGATATCGTGCAGTCCATGGGTAACCGAGGGAGCCAAGATGTTGGTGGTTCCTGCGAGCTTGCCGCAAAGCAGCAGCTCGTCACCGCTGATCTCACTTCCATCATAATTCAGCTCATCTCGACTGCCGGCTCCATGCTTCCTTCCATGAAGACCCCGCTCCTTAGCAGCAATCCAGCGGTCAGGCAACTCAACACGCCTGGTTCTCCCATGGGCTTTGGCTCGATTGTGAATCAGCGGCCAAGCACAGTCAGGGAGGAGATGGTTCCTGACATTAGCAAGACCTCTGATTATGAGGAGCTGATGAATACCCTTAACACAGCGCATGATGAAAAGGATGATCTGATCAACTGCCCAAATCCTTGTGTCGGGGAAGGGCCTGAGCCGGTTCCGATGGAAGACCATGACGTGAAGGAGAGCGATGATGGTGGTGAGGCAGAGCACCTCCCCCCTGGTTCTTATGTGGTCTTGCAGTTGGAGAAGGAGGAAATTTTAGCACCACATACTCATTTCTGTGTGATATGTGGGAAGGGTTTCAAGAGGGATGCTAACCTAAGGATGCACATGAGGGGCCATGGAGACGAGTACAAGACTCCCGCAGCTCTTGCCAAACCCATGAGAGATTCTGGCTCAGATCCTACACCAGTTACAAGGTACTCGTGCCCATATGTCGGTTGCAAGCGGAACAAAGAGCACAGGAAGTTCCAGCCCCTCAAGACAATCTTGTGTGTGAAGAACCACTACAAGAGAAGCCACTGTGACAAGAGGTATACCTGCAGCCGATGCAATACCAAGAAGTTCTCAGTCATTGCGGACTTGAAGACTCATGAGAAGCACTGCGGGCGTGACAAGTGGCTCTGCTCATGTGGAACAACTTTCTCAAGAGAGGACAAGCTGTTCGGCCATGTCGCGCTTTTCCAAGGGCACACACCTGCTCTAGCAATGGATGATATTAAAGCAACAGGAGCATCGGAGCAGCCTCAGGGGAGCGAGGCGATGGACGACATGGTGGGGAGCACAGGGTATAACTTCCCAGGCAGCACGTCTGATGGTATTCCGAATCTAGACATGAAAGTTGCCGATGACACACGTGGTTATTTCTCGCCCTTGAACTTCGACCCGTGCTTCGGCGCCCTCGATGACTTCGCCCCCCCTGGATTCGACATCTCCGAGAACCCCTTCTCCTTCCTGCCTTCGGGACCGGGTTCCTGCAGCTTTGGGCAGCTTAGTGGAGACAGCTGATGGAGATCGTCACCGTAGACAACCATGATGGTGTCGATCGTTATGTATGAATGTTATATGTATGATCATCTCAGTTCTTCCTCTATTCAGGGAAATCACTAGTG
>SEQ_TaSTOP1_ Viloso Mole _Genome A
ATGAAAGCTTCGTCGTCGATGGCAAGCGACGCTTCAGGGAACACTGACCCTGGCCAACAGGGTGCTCGTTTCAGTTCCATGGACCAGTCTTGCTTTGCAAGACCTGGCCAGTCAATCCCTGGCTACCCCCCATTCTTTGGCCCTCAGTCTTCCAACTTTTACCTTCCTGATGACAGTGTGGCTAAAGCGTGTGATCCGTTCGAGCCGAATCCTCCTCAGAACAATCCTGTGGCAGATTGGGACCCTCAGGCCATGCTGAGCAACCTAACCTTCCTTGAGCAGAAGATCAAGCAGGTGAAAGATATCGTGCAGTCCATGGGTAACCGAGGGAGCCAAGATGTTGGTGGTTCCTGCGAGCTTGCCGCAAAGCAGCAGCTCGTCACCGCTGATCTCACTTCCATCATAATTCAGCTCATCTCGACTGCCGGCTCCATGCTTCCTTCCATGAAGACCCCGCTGCTTAGCAGCAATCCAGCGGTCAGGCAGCTCAACACGCCTGGTTCTCCCATGGGCTTTGGCTCGATTGTGAATCAGCGGCCAAGCACAGTCAGGGAGGAGATGGTTCCTGACATTACCAAGACCCCTGACTATGAGGATCTGATGAATACCCTTAATCCAGCCCATGATGAAAAGGATGATCTGATCAAATGCCCAAATCCTTGTGTTGGGGAAGGGCCTGAGCCGGTTCCGATGGAAGACCATGACGTGAAGGAGAGCGATGATGGTGGCGAGGCAGAGCATCTCCCCCCTGGTTCTTATGTGGTCTTGCAATTGGAGAAGGAGGAGATTTTAGCACCACACACTCATTTCTGTGTGATATGTGGCAAGGGTTTCAAGAGGGATGCTAACCTAAGGATGCACATGAGGGGCCATGGAGACGAGTACAAAACTCCCGCAGCTCTTGCCAAACCCATGAGAGATTCTGTCTCAGATCCTACACCAGTTACAAGGTACTCGTGCCCATATGTCGGTTGCAAGCGCAACAAAGAGCACAGGAAGTTCCAGCCCCTCAAGACAATCTTGTGTGTGAAGAACCACTACAAGAGAAGCCATTGCGACAAGAGGTATACCTGCAGCCGATGCAACACCAAGAAGTTCTCAGTCATTGCGGACTTGAAGACTCATGAGAAGCACTGTGGGCGTGACAAGTGGCTCTGCTCATGTGGAACAACTTTCTCAAGAAAGGACAAGCTGTTCGGCCATGTCGCGCTTTTCCAAGGGCACACACCTGCTCTTCCAATGGATGATATTAAAGCAACAGGAGCATCGGAGCAGAGGAGCGAGGCGATGGACGACATGGTGGGGAGCACAGGGTATAACTTCCCAGGCAGCACGTCTGATGGTATTCCGAATCTAGACATGAAAGTTGCCGATGACACACGTGGTTATTTCTCTCCCTTGAACTTCGACCCCTGCTTCGGCGCCCTCGATGACTTCGCCCGACCTGGATTCGACATCTCCGAGAACCCCTTCTCCTTCCTGCCTTCAGGACCGGGTTCTTGCAGCTTTGGGCAGCTTAGTGGAGACAGCTGATGGAGTTCGTCGCCGTAGACAACCATGATGGTGTCGATTGTTATGTATGAATGTTATATGTATGACCGTCTCAGTTCTTCCTCTATTCAGGGAGCCATTTTGG
>SEQ_TaSTOP1_ Viloso Mole _Genome B
ATGAAAGCTTCGTCGTCGATGGCAAGCGACGCGTCAGGGAACACTGAACCTGGCCAACAGGGCGTTCGTTTCAGTTCCATGGACCAGTCTTGCTTTGCAAGACCTGGCCAGTCAATCCCTGGCTACCCCCCATTCTTTGGCCCTCAATCTTCCAACTTTTACCTTCCTGATGACAGTGTGGCTAAAGCGTGTGATCCGTTTGAACCGAATCCTCCACAGAGCAATCCTGTGGCAGACTGGGATCCTCAGGCCATGCTGAGCAACCTAACCTTCCTTGAGCAGAAGATCAAGCAGGTGAAAGATATCGTGCAGTCCATGGGTAACCGAGGGAGCCAAGATGTTGGTGGTTCCTGCGAGCTTGCCGCAAAGCAGCAGCTCGTTACCGCTGATCTCACTTCCATCATAATTCAGCTCATCTCGACTGCCGGCTCCATGCTTCCTTCCATGAAGACCCCGCTCCTTAGCAGCAATCCAGCGGTCAGGCAACTCAACACGCCTGGTTCTCCCATGGGCTTTGGCTCGATTGTGAATCAGCGGCCAAGCACAGTCAGGGAGGAGATGGTTCCTGACATTAGCAAGACCTCTGATTATGAGGAGCTGATGAATACCCTTAACACAGCGCATGATGAAAAGGATGATCTGATCAAATGCCCAAATCCTTGTGTCGGGGAAGGGCCTGAGCCGGTTCCGATGGAAGACCATGATGTGAAGGAGAGCGATGATGGTGGTGAGGCAGAGCACCTCCCCCCTGGTTCTTATGTGGTCTTGCAGTTGGAGAAGGAGGAAATTTTAGCACCACATACTCATTTCTGTGTGATATGTGGGAAGGGTTTCAAGAGGGATGCTAACCTAAGGATGCACATGAGGGGCCATGGAGACGAGTACAAGACTCCCGCAGCTCTTGCCAAACCCATGAGAGATTCTGGCTCAGATCCTACACCAGTTACAAGGTACTCGTGCCCATATGTCGGTTGCAAGCGGAACAAAGAGCACAGGAAGTTCCAGCCCCTCAAGACAATCTTGTGTGTGAAGAACCACTACAAGAGAAGCCACTGTGACAAGAGGTATACCTGCAGCCGATGCAATACCAAGAAGTTCTCAGTCATTGCGGACTTGAAGACTCATGAGAAGCACTGCGGGCGTGACAAGTGGCTCTGCTCATGTGGAACAACTTTCTCAAGAAAGAACAAGCTGTTCGGCCATGTCGCGCTTTTCCAAGGGCACACACCTGCTCTTCCAATGGATGATATTAAAGCAACAGGAGCATCGGAGCAGCCTCAGGGGAGCGAGGCGATGGACGACATGGTGGGGAGCACAGGGTATAACTTCCCAGGCAGCACGTCTGATGGTATTCCGAATCTAGACATGAAAGTTGCCGATGACACACGTGGTTATTTCTCGCCCTTGAACTTCGACCCGTGCTTCGGCGCCCTCGATGACTTCGCCCGCCCTGGATTCGACATCTCCGAGAACCCCTTCTCCTTCCTGCCTTCGGGACCGGGTTCCTGCAGCTTTGGGCAGCTTAGTGGAGACAGCTGATGGAGATCGTCACCGTAGACAACCATGATGGTGTCGATCGTTATGTATGAATGTTATATGTATGATCATCTCAGTTCTTCCTCTATTCAGGGAGCCATTTTGG
>SEQ_TaSTOP1_ Viloso Mole _Genome D
ATGAAAGCTTCGTCGTCGATGGCAAGCGACGCGTCAGGGAACACTGAACCTGGCCAACAGGGTGTTCGTTTCAGTTCCATGGACCAGTCTTGCTTTGCAAGACCTGGCCAGTCAATCCCTGGCTACCCCCCATTCTTTGGCCCTCAATCTTCCAACTTTTACCTTCCTGATGACAGTGTGGCTAAAGCGTGTGATCTGTTTGAACCGAATCCTCCACAGAACAATCCTGTGGCTGATTGGGATCCTCAGGCCATGCTGAGCAACCTAACCTTCCTTGAGCAGAAGATCAAGCAGGTGAAAGATATCGTGCAGTCCATGGGTAACCGAGGGAGCCAAGATGCTGGTGGTTCCTGCGAGCTTGCTGCAAAGCAGCAGCTCGTCACCGCCGATCTCACTTCCATCATAATTCAGCTCATCTCAACTGCCGGCTCCATGCTTCCTTCCATGAAGACCCCGCTCCTTAGCAGCAATCCAGCGGTCAGGCAGCTCAACACGCCTGGTTCTCCCATGGGCTTTGGCTCGATCGTGAATCAGCGGCCAAGCACAATCAGGGAGGAGGTGGTTAATGACATTAGCAAGACCTCTGACTATGAGGAGCTGATGAATACCCTTAACACAGCCCACGATGAAAAGGATGATCTGATCAAATGCCCAAATCCTTGTGTCGGGGAAGGACCTGAGCCGGTTCCGATGGAAGACCATGACGTGAAGGAGAGCGATGATGGTGGCGAGGCAGAGCACCTCCCCCCTGGTTCTTATGTGGTCTTGCAATTGGAGAAGGAGGAAATTCTAGCACCACACACTCATTTCTGTGTGATATGTGGCAAGGGTTTCAAGAGGGATGCTAACCTAAGGATGCACATGAGGGGCCATGGAGACGAGTACAAGACTCCCGCAGCTCTTGCCAAACCCATGAGAGATTCTGGCTCAGATCCTACACCAGTTACAAGGTACTCGTGCCCATATGTCGGTTGCAAGCGGAACAAAGAGCACAGGAAGTTCCAGCCTCTCAAGACAATCTTGTGTGTGAAGAACCACTACAAGAGAAGCCACTGTGACAAGAGGTATACCTGCAGCCGATGCAACACCAAGAAGTTCTCAGTCATTGCTGACTTGAAGACTCATGAGAAGCACTGCGGGCGTGACAAGTGGCTCTGCTCATGTGGAACGACCTTCTCAAGAAAAGACAAGCTGTTCGGGCATGTCGCGCTTTTCCAAGGGCACACACCTGCTCTTCCAATGGATGATATCAAAGCATCAGGAGCATTGGAGCAGCCTCAGGGGAGCGAGGCGATGGACGACATGGTGGCGAGCACAGGGTATAACTTCCCAGGCAGCACGTCTGATGGTATTCCGAATCTAGACATGAAAGTTGCCGATGACACACGTGGTTATTTCTCGCCCTTGAACTTCGACCCGTGCTTCGGTGCCCTCGATGACTTTGCTCGCCCTGGATTCGACATCTCTGAGAACCCCTTCTCCTTCCTGCCTTCGGGACCGGGGTCGTGCAGTTTTGGGCAGATTAGTGGCGACAGCTAATGGAGCTCGTCACCGTTGACAACCATGATGGTGTCGATCGTTATGTATGAATGTTATATGTATGACCGTCTTAGTTCTTCCTCTATTCAGGGAGCCATTTTGG
>SEQ_TaSTOP1_ Saloio _Genome A
ATGAAAGCTTCGTCGTCGATGGCAAGCGACGCTTCAGGGAACACTGACCCTGGCCAACAGGGTGCTCGTTTCAGTTCCATGGACCAGTCTTGCTTTGCAAGACCTGGCCAGTCAATCCCTGGCTACCCCCCATTCTTTGGCCCTCAGTCTTCCAACTTTTACCTTCCTGATGACAGTGTGGCTAAAGCGTGTGATCCGTTCGAGCCGAATCCTCCTCAGAACAATCCTGTGGCAGATTGGGACCCTCAGGCCATGCTGAGCAACCTAACCTTCCTTGAGCAGAAGATCAAGCAGGTGAAAGATATCGTGCAGTCCATGGGTAACCGAGGGAGCCAAGATGTTGGTGGTTCCTGCGAGCTTGCCGCAAAGCAGCAGCTCGTCACCGCTGATCTCACTTCCATCATAATTCAGCTCATCTCGACTGCCGGCTCCATGCTTCCTTCCATGGAGACCCCGCTGCTTAGCAGCAATCCAGCGGTCAGGCAGCTCAACACGCCTGGTTCTCCCATGGGCTTTGGCTCGATTGTGAATCAGCGGCCAAGCACAGTCAGGGAGGAGATGGTTCCTGACATTACCAAGACCCCTGACTATGAGGATCTGATGAATACCCTTAATCCAGCCCATGATGAAAAGGATGATCTGATCAAATGCCCAAATCCTTGTGTTGGGGAAGGGCCTGAGCCGGTTCCGATGGAAGACCATGACGTGAAGGAGAGCGATGATGGTGGCGAGGCAGAGCATCTCCCCCCTGGTTCTTATGTGGTCTTACAATTGGAGAAGGAGGAGATTTTAGCACCACACACTCATTTCTGTGTGATATGTGGCAAGGGTTTCAAGAGGGATGCTAACCTAAGGATGCACATGAGGGGCCATGGAGACGAGTACAAAACTCCCGCAGCTCTTGCCAAACCCATGAGAGATTCTGTCTCAGATCCTACACCAGTTACAAGGTACTCGTGCCCATATGTCGGTTGCAAGCGCAACAAAGAGCACAGGAAGTTCCAGCCCCTCAAGACAATCTTGTGTGTGAAGAACCACTACAAGAGAAGCCATTGCGACAAGAGGTATACCTGCAGCCGATGCAACACCAAGAAGTTCTCAGTCATTGCGGACTTGAAGACTCATGAGAAGCACTGTGGGCGTGACAAGTGGCTCTGCTCATGTGGAACAACTTTCTCAAGAAAGGACAAGCTGTTCGGCCATGTCGCGCTTTTCCAAGGGCACACACCTGCTCTTCCAATGGATGATATTAAAGCAACAGGAGCATCGGAGCAGAGGAGCGAGGCGATGGACGACATGGTGGGGAGCACAGGGTATAACTTCCCAGGCAGCACGTCTGATGGTATTCCGAATCTAGACATGAAAGTTGCCGATGACACACGTGGTTATTTCTCTCCCTTGAACTTCGACCCCTGCTTCGGCGCCCTCGATGACTTCGCCCGACCTGGATTCGACATCTCCGAGAACCCCTTCTCCTTCCTGCCTTCAGGACCGGGTTCTTGCAGCTTTGGGCAGCTTAGTGGAGACAGCTGATGGAGTTCGTCGCCGTAGACAACCATGATGGTGTCGATTGTTATGTATGAATGTTATATGTATGACCGTCTCAGTTCTTCCTCTATTCAGGGAGCCATTTTGG
>SEQ_TaSTOP1_ Saloio _Genome B
ATGAAAGCTTCGTCGTCGATGGCAAGCGACGCGTCAGGGAACACTGAACCTGGCCAACAGGGCGTTCGTTTCAGTTCCATGGACCAGTCTTGCTTTGCAAGACCTGGCCAGTCAATCCCTGGCTACCCCCCATTCTTTGGCCCTCAATCTTCCAACTTTTACCTTCCTGATGACAGTGTGGCTAAAGCGTGTGATCCGTTTGAACCGAATCCTCCACAGAGCAATCCTGTGGCAGACTGGGATCCTCAGGCCATGCTGAGCAACCTAACCTTCCTTGAGCAGAAGATCAAGCAGGTGAAAGATATCGTGCAGTCCATGGGTAACCGAGGGAGCCAAGATGTTGGTGGTTCCTGCGAGCTTGCCGCAAAGCAGCAGCTCGTCACCGCTGATCTCACTTCCATCATAATTCAGCTCATCTCGACTGCCGGCTCCATGCTTCCTTCCATGAAGACCCCGCTCCTTAGCAGCAATCCAGCGGTCAGGCAACTCAACACGCCTGGTTCTCCCATGGGCTTTGGCTCGATTGTGAATCAGCGGCCAAGCACAGTCAGGGAGGAGATGGTTCCTGACATTAGCAAGACCTCTGATTATGAGGAGCTGATGAATACCCTTAACACAGCGCATGATGAAAAGGATGATCTGATCAAATGCCCAAATCCTTGTGTCGGGGAAGGGCCTGAGCCGGTTCCGATGGAAGACCATGACGTGAAGGAGAGCGATGATGGTGGTGAGGCAGAGCACCTCCCCCCTGGTTCTTATGTGGTCTTGCAGTTGGAGAAGGAGGAAATTTTAGCACCACATACTCATTTCTGTGTGATATGTGGGAAGGGTTTCAAGAGGGATGCTAACCTAAGGATGCACATGAGGGGCCATGGAGACGAGTACAAGACTCCCGCAGCTCTTGCCAAACCCATGAGAGATTCTGGCTCAGATCCTACACCAGTTACAAGGTACTCGTGCCCATATGTCGGTTGCAAGCGGAACAAAGAGCACAGGAAGTTCCAGCCCCTCAAGACAATCTTGTGTGTGAAGAACCACTACAAGAGAAGCCACTGTGACAAGAGGTATACCTGCAGCCGATGCAATACCAAGAAGTTCTCAGTCATTGCGGACTTGAAGACTCATGAGAAGCACTGCGGGCGTGACAAGTGGCTCTGCTCATGTGGAACAACTTTCTCAAGAAAGGACAAGCTGTTCGGCCATGTCGCGCTTTTCCAAGGGCACACACCTGCTCTTCCAATGGATGATATTAAAGCAACAGGAGCATCGGAGCAGCCTCAGGGGAGCGAGGCGATGGGCGACATGGTGGGGAGCACAGGGTATAACTTCCCAGGCAGCACGTCTGATGGTATTCCGAATCTAGACATGAAAGTTGCCGATGACACACGTGGTTATTTCTCGCCCTTGAACTTCGACCCGTGCTTCGGCGCCCTTGATGACTTCGCCCGCCCTGGATTCGACATCTCCGAGAACCCCTTCTCCTTCCTGCCTTCGGGACCGGGTTCCTGCAGCTTTGGGCAGCTTAGTGGAGACAGCTGATGGAGATCGTCACCGTAGACAACCATGATGGTGTCGATCGTTATGTATGAATGTTATATGTATGATCATCTCAGTTCTTCCTCTATTCAGGGAGCCATTTTGG
>SEQ_TaSTOP1_ Saloio _Genome D
ATGAAAGCTTCGTCGTCGATGGCAAGCGACGCGTCAGGGAACACTGAACCTGGCCAACAGGGTGTTCGTTTCAGTTCCATGGACCAGTCTTGCTTTGCAAGACCTGGCCAGTCAATCCCTGGCTACCCCCCATTCTTTGGCCCTCAATCTTCCAACTTTTACCTTCCTGATGACAGTGTGGCTAAAGCGTGTGATCTGTTTGAACCGAATCCTCCACAGAACAATCCTGTGGCTGATTGGGATCCTCAGGCCATGCTGAGCAACCTAACCTTCCTTGAGCAGAAGATCAAGCAGGTGAAAGATATCGTGCAGTCCATGGGTAACCGAGGGAGCCAAGATGCTGGTGGTTCCTGCGAGCTTGCTGCAAAGCAGCAGCTCGTCACCGCCGATCTCACTTCCATCATAATTCAGCTCATCTCAACTGCCGGCTCCATGCTTCCTTCCATGAAGACCCCGCTCCTTAGCAGCAATCCAGCGGTCAGGCAGCTCAACACGCCTGGTTCTCCCATGGGCTTTGGCTCGATCGTGAATCAGCGGCCAAGCACAATCAGGGAGGAGGTGGTTAATGACATTAGCAAGACCTCTGACTATGAGGAGCTGATGAATACCCTTAACACAGCCCACGATGAAAAGGATGATCTGATCAAATGCCCAAATCCTTGTGTCGGGGAAGGACCTGAGCCGGTTCCGATGGAAGACCATGACGTGAAGGAGAGCGATGATGGTGGCGAGGCAGAGCACCTCCCCCCTGGTTCTTATGTGGTCTTGCAATTGGAGAAGGAGGAAATTCTAGCACCACACACTCATTTCTGTGTGATATGTGGCAAGGGTTTCAAGAGGGATGCTAACCTAAGGATGCACATGAGGGGCCATGGAGACGAGTACAAGACTCCCGCAGCTCTTGCCAAACCCATGAGAGATTCTGGCTCAGATCCTACACCAGTTACAAGGTACTCGTGCCCATATGTCGGTTGCAAGCGGAACAAAGAGCACAGGAAGTTCCAGCCTCTCAAGACAATCTTGTGTGTGAAGAACCACTACAAGAGAAGCCACTGTGACAAGAGGTATACCTGCAGCCGATGCAACACCAAGAAGTTCTCAGTCATTGCTGACTTGAAGACTCATGAGAAGCACTGCGGGCGTGACAAGTGGCTCTGCTCATGTGGAACGACCTTCTCAAGAAAAGACAAGCTGTTCGGGCATGTCGCGCTTTTCCAAGGGCACACACCTGCTCTTCCAATGGATGATATCAAAGCATCAGGAGCATTGGAGCAGCCTCAGGGGAGCGAGGCGATGGACGACATGGTGGCGAGCACAGGGTATAACTTCCCAGGCAGCACGTCTGATGGTATTCCGAATCTAGACATGAAAGTTGCCGATGACACACGTGGTTATTTCTCGCCCTTGAACTTCGACCCGTGCTTCGGTGCCCTCGATGACTTTGCTCGCCCTGGATTCGACATCTCTGAGAACCCCTTCTCCTTCCTGCCTTCAGGACCGGGGTCGTGCAGTTTTGGGCAGCTTAGTGGCGACAGCTAATGGAGCTCGTCACCGTTGACAACCATGATGGTGTCGATCGTTATGTATGAATGTTATATGTATGACCGTCTTAGTTCTTCCTCTATTCAGGGAGCCATTTTGG
>SEQ_TaSTOP1_ Ruivo _Genome D
ATGAAAGCTTCGTCGTCGATGGCAAGCGACGCGTCAGGGAACACTGAACCTGGCCAACAGGGTGTTCGTTTCAGTTCCATGGACCAGTCTTGCTTTGCAAGACCTGGCCAGTCAATCCCTGGCTACCCCCCATTCTTTGGCCCTCAATCTTCCAACTTTTACCTTCCTGATGACAGTGTGGCTAAAGCGTGTGATCTGTTTGAACCGAATCCTCCACAGAACAATCCTGTGGCTGATTGGGATCCTCAGGCCATGCTGAGCAACCTAACCTTCCTTGAGCAGAAGATCAAGCAGGTGAAAGATATCGTGCAGTCCATGGGTAACCGAGGGAGCCAAGATGCTGGTGGTTCCTGCGAGCTTGCTGCAAAGCAGCAGCTCGTCACCGCCGATCTCACTTCCATCATAATTCAGCTCATCTCAACTGCCGGCTCCATGCTTCCTTCCATGAAGACCCCGCTCCTTAGCAGCAATCCAGCGGTCAGGCAGCTCAACACGCCTGGTTCTCCCATGGGCTTTGGCTCGATCGTGAATCAGCGGCCAAGCACAATCAGGGAGGAGGTGGTTAATGACATTAGCAAGACCTCTGACTATGAGGAGCTGATGAATACCCTTAACACAGCCCACGATGAAAAGGATGATCTGATCAAATGCCCAAATCCTTGTGTCGGGGAAGGACCTGAGCCGGTTCCGATGGAAGACCATGACGTGAAGGAGAGCGATGATGGTGGCGAGGCAGAGCACCTCCCCCCTGGTTCTTATGTGGTCTTGCAATTGGAGAAGGAGGAAATTCTAGCACCACACACTCATTTCTGTGTGATATGTGGCAAGGGTTTCAAGAGGGATGCTAACCTAAGGATGCACATGAGGGGCCATGGAGACGAGTACAAGACTCCCGCAGCTCTTGCCAAACCCATGAGAGATTCTGGCTCAGATCCTACACCAGTTACAAGGTACTCGTGCCCATATGTCGGTTGCAAGCGGAACAAAGAGCACAGGAAGTTCCAGCCTCTCAAGACAATCTTGTGTGTGAAGAACCACTACAAGAGAAGCCACTGTGACAAGAGGTATACCTGCAGCCGATGCAACACCAAGAAGTTCTCAGTCATTGCTGACTTGAAGACTCATGAGAAGCACTGCGGGCGTGACAAGTGGCTCTGCTCATGTGGAACGACCTTCTCAAGAAAAGACAAGCTGTTCGGGCATGTCGCGCTTTTCCAAGGGCACACACCTGCTCTTCCAATGGATGATATCAAAGCATCAGGAGCATTGGAGCAGCCTCAGGGGAGCGAGGCGATGGACGACATGGTGGCGAGCACAGGGTATAACTTCCCAGGCAGCACGTCTGATGGTATTCCGAATCTAGACATGAAAGTTGCCGATGACACACGTGGTTATTTCTCGCCCTTGAACTTCGACCCGTGCTTCGGTGCCCTCGATGACTTTGCTCGCCCTGGATTCGACATCTCTGAGAACCCCTTCTCCTTCCTGCCTTCAGGACCGGGGTCGTGCAGTTTTGGGCAGCTTAGTGGCGACAGCTAATGGAGCTCGTCACCGTTGACAACCATGATGGTGTCGATCGTTATGTATGAATGTTATATGTATGACCGTCTTAGTTCTTCCTCTATTCAGGGAGCCATTTTGG


Protein sequences of TaSTOP1 homoeologues gene in different bread wheat genotypes
>TaSTOP1A_Barbela
MKASSSMASDASGNTDPGQQGARFSSMDQSCFARPGQSIPGYPPFFGPQSSNFYLPDDSVAKACDPFEPNPPQNNPVADWDPQAMLSNLTFLEQKIKQVKDIVQSMGNRGSQDVGGSCELAAKQQLVTADLTSIIIQLISTAGSMLPSMKTPLLSSNPAVRQLNTPGSPMGFGSIVNQRPSTVREEMVPDITKTPDYEDLMNTLNPAHDEKDDLIKCPNPCVGEGPEPVPMEDHDVKESDDGGEAEHLPPGSYVVLQLEKEEILAPHTHFCVICGKGFKRDANLRMHMRGHGDEYKTPAALAKPMRDSVSDPTPVTRYSCPYVGCKRNKEHRKFQPLKTILCVKNHYKRSHCDKRYTCSRCNTKKFSVIADLKTHEKHCGRDKWLCSCGTTFSRKDKLFGHVALFQGHTPALPMDDIKATGASEQRSEAMDDMVGSTGYNFPGSTSDGIPNLDMKVADDTRGYFSPLNFDPCFGALDDFARPGFDISENPFSFLPSGPGSCSFGQLSGDS
> TaSTOP1B_Barbela
MKASSSMASDASGNTEPGQQGVRFSSMDQSCFARPGQSIPGYPPFFGPQSSNFYLPDDSVAKACDPFEPNPPQSNPVADWDPQAMLSNLTFLEQKIKQVKDIVQSMGNRGSQDVGGSCELAAKQQLVTADLTSIIIQLISTAGSMLPSMKTPLLSSNPAVRQLNTPGSPMGFGSIVNQRPSTVREEMVPDISKTSDYEELMNTLNTAHDEKDDLIKCPNPCVGEGPEPVPMEDHDVKESDDGGEAEHLPPGSYVVLQLEKEEILAPHTHFCVICGKGFKRDANLRMHMRGHGDEYKTPAALAKPMRDSGSDPTPVTRYSCPYVGCKRNKEHRKFQPLKTILCVKNHYKRSHCDKRYTCSRCNTKKFSVIADLKTHEKHCGRDKWLCSCGTTFSRKNKLFGHVALFQGHTPALPMDDIKATGASEQPQGSEAMDDMVGSTGYNFPGSTSDGIPNLDMKVADDTRGYFSPLNFDPCFGALDDFARPGFDISENPFSFLPSGPGSCSFGQLSGDS
> TaSTOP1D_Barbela
MKASSSMASDASGNTEPGQQGVRFSSMDQSCFARPGQSIPGYPPFFGPQSSNFYLPDDSVAKACDLFEPNPPQNNPVADWDPQAMLSNLTFLEQKIKQVKDIVQSMGNRGSQDAGGSCELAAKQQLVTADLTSIIIQLISTAGSMLPSMKTPLLSGNPAVRQLNTPGSPMGFGSIVNQRPSTIREEVVNDISKTSDYEELMNTLNTAHDEKDDLIKCPNPCVGEGPEPVPMEDHDVKESDDGGEAEHLPPGSYVVLQLEKEEILAPHTHFCVICGKGFKRDANLRMHMRGHGDEYKTPAALAKPMRDPGSDPTPVTRYSCPYVGCKRNKEHRKFQPLKTILCVKNHYKRSHCDKRYTCSRCNTKKFSVIADLKTHEKHCGRDKWLCSCGTTFSRKDKLFGHVALFQGHTPALPMDDIKASGALEQPQGSEAMDDMVASTGYNFPGSTSDGIPNLDMKVADDTRGYFSPLNFDPCFGALDDFARPGFDISENPFSFLPSGPGSCSFGQLSGDS
> TaSTOP1A_Anahuac
MKASSSMASDASGNTDPGQQGARFSSMDQSCFARPGQSIPGYPPFFGPQSSNFYLPDDSVAKACDPFEPNPPQNNPVADWDPQAMLSNLTFLEQKIKQVKDIVQSMGNRGSQDVGGSCELAAKQQLVTADLTSIIIQLISTAGSMLPSMKTPLLSSNPAVRQLNTPGSPMGFGSIVNQRPSTVREEMVPDITKTPDYEDLMNTLNPAHDEKDDLIKCPNPCVGEGPEPVPMEDHDVKESDDGGEAEHLPPGSYVVLQLEKEEILAPHTHFCVICGKGFKRDANLRMHMRGHGDEYKTPAALAKPMRDSVSDPTPVTRYSCPYVGCKRNKEHRKFQPLKTILCVKNHYKRSHCDKRYTCSRCNTKKFSVIADLKTHEKHCGRDKWLCSCGTTFSRKDKLFGHVALFQGHTPALPMDDIKATGASEQRSEAMDDMVGSTGYNFPGSTSDGIPNLDMKVADDTRGYFSPLNFDPCFGALDDFARPGFDISENPFSFLPSGPGSCSFGQLSGDS
> TaSTOP1B_Anahuac
MKASSSMASDASGNTEPGQQGVRFSSMDQSCFARPGQSIPGYPPFFGPQSSNFYLPDDSVAKACDPFEPNPPQSNPVADWDPQAMLSNLTFLEQKIKQVKDIVQSMGNRGSQDVGGSCELAAKQQLVTADLTSIIIQLISTAGSMLPSMKTPLLSSNPAVRQLNTPGSPMGFGSIVNQRPSTVREEMVPDISKTSDYEELMNTLNTAHDEKDDLIKCPNPCVGEGPEPVPMEDHDVKESDDGGEAEHLPPGSYVVLQLEKEEILAPHTHFCVICGKGFKRDANLRMHMRGHGDEYKTPAALAKPMRDSGSDPTPVTRYSCPYVGCKRNKEHRKFQPLKTILCVKNHYKRSHCDKRYTCSRCNTKKFSVIADLKTHEKHCGRDKWLCSCGTTFSRKDKLFGHVALFQGHTPALPMDDIKATGASEQPQGSEAMDDMVGSTGYNFPGSTSDGIPNLDMKVADDTRGYFSPLNFDPCFGALDDFARPGFDISENPFSFLPSGPGSCSFGQLSGDS
> TaSTOP1D_Anahuac
MKASSSMASDASGNTEPGRQGVRFSSMDQSCFARPGQSIPGYPPFFGPQSSNFYLPDDSVAKACDLFEPNPPQNNPVADWDPQAMLSNLTFLEQKIKQVKDIVQSMGNRGSQDAGGSCELAAKQQLVTADLTSIIIQLISTAGSMLPSMKTPLLSSNPAVRQLNTPGSPMGFGSIVNQRPSTIREEVVNDISKTSDYEELMNTLNTAHDEKDDLIKCPNPCVGEGPEPVPMEDHDVKESDDGGEAEHLPPGSYVVLQLEKEEILAPHTHFCVICGKGFKRDANLRMHMRGHGDEYKTPAALAKPMRDSGSDPTPVTRYSCPYVGCKRNKEHRKFQPLKTILCVKNHYKRSHCDKRYTCSRCNTKKFSVIADLKTHEKHCGRDKWLCSCGTTFSRKDKLFGHVALFQGHTPALPMDDIKASGALEQPQGSEAMDDMVASTGYNFPGSTSDGIPNLDMKVADDTRGYFSPLNFDPCFGALDDFARPGFDISENPFSFLPSGPGSCSFGQLSGDS
> TaSTOP1A_CS
MKASSSMASDASGNTDPGQQGARFSSMDQSCFARPGQSIPGYPPFFGPQSSNFYLPDDSVAKACDPFEPNPPQNNPVADWDPQAMLSNLTFLEQKIKQVKDIVQSMGNRGSQDVGGSCELAAKQQLVTADLTSIIIQLISTAGSMLPSMKTPLLSSNPAVRQLNTPGSPMGFGSIVNQRPSTVREEMVPDITKTPDYEDLMNTLNPAHDEKDDLIKCPNPCVGEGPEPVPMEDHDVKESDDGGEAEHLPPGSYVVLQLEKEEILAPHTHFCVICGKGFKRDANLRMHMKGHGDEYKTPAALAKPMRDSVSDPTPVTRYSCPYVGCKRNKEHRKFQPLKTILCVKNHYKRSHCDKRYTCSRCNTKKFSVIADLKTHEKHCGRDKWLCSCGTTFSRKDKLFGHVALFQGHTPALPMDDIKATGASEQRSEAMDDMVGSTGYNFPGSTSDGIPNLDMKVADDTRGYFSPLNFDPCFGALDDFARPGFDISENPFSFLPSGPGSCSFGQLSGDS
> TaSTOP1B_CS
MKASSSMASDASGNTEPGQQGVRFSSMDQSCFARPGQSIPGYPPFFGPQSSNFYLPDDSVAKACDPFEPNPPQSNPVADWDPQAMLSNLTFLEQKIKQVKDIVQSMGNRGSQDVGGSCELAAKQQLVTADLTSIIIQLISTAGSMLPSMKTPLLSSNPAVRQLNTPGSPMGFGSIVNQRPSTVREEMVPDISKTSDYEELMNTLNTAHDEKDDLINCPNPCVGEGPEPVPMEDHDVKESDDGGEAEHLPPGSYVVLQLEKEEILAPHTHFCVICGKGFKRDANLRMHMRGHGDEYKTPAALAKPMRDSGSDPTPVTRYSCPYVGCKRNKEHRKFQPLKTILCVKNHYKRSHCDKRYTCSRCNTKKFSVIADLKTHEKHCGRDKWLCSCGTTFSREDKLFGHVALFQGHTPALAMDDIKATGASEQPQGSEAMDDMVGSTGYNFPGSTSDGIPNLDMKVADDTRGYFSPLNFDPCFGALDDFAPPGFDISENPFSFLPSGPGSCSFGQLSGDS
> TaSTOP1A_VilosoMole
MKASSSMASDASGNTDPGQQGARFSSMDQSCFARPGQSIPGYPPFFGPQSSNFYLPDDSVAKACDPFEPNPPQNNPVADWDPQAMLSNLTFLEQKIKQVKDIVQSMGNRGSQDVGGSCELAAKQQLVTADLTSIIIQLISTAGSMLPSMKTPLLSSNPAVRQLNTPGSPMGFGSIVNQRPSTVREEMVPDITKTPDYEDLMNTLNPAHDEKDDLIKCPNPCVGEGPEPVPMEDHDVKESDDGGEAEHLPPGSYVVLQLEKEEILAPHTHFCVICGKGFKRDANLRMHMRGHGDEYKTPAALAKPMRDSVSDPTPVTRYSCPYVGCKRNKEHRKFQPLKTILCVKNHYKRSHCDKRYTCSRCNTKKFSVIADLKTHEKHCGRDKWLCSCGTTFSRKDKLFGHVALFQGHTPALPMDDIKATGASEQRSEAMDDMVGSTGYNFPGSTSDGIPNLDMKVADDTRGYFSPLNFDPCFGALDDFARPGFDISENPFSFLPSGPGSCSFGQLSGDS
> TaSTOP1B_VilosoMole
MKASSSMASDASGNTEPGQQGVRFSSMDQSCFARPGQSIPGYPPFFGPQSSNFYLPDDSVAKACDPFEPNPPQSNPVADWDPQAMLSNLTFLEQKIKQVKDIVQSMGNRGSQDVGGSCELAAKQQLVTADLTSIIIQLISTAGSMLPSMKTPLLSSNPAVRQLNTPGSPMGFGSIVNQRPSTVREEMVPDISKTSDYEELMNTLNTAHDEKDDLIKCPNPCVGEGPEPVPMEDHDVKESDDGGEAEHLPPGSYVVLQLEKEEILAPHTHFCVICGKGFKRDANLRMHMRGHGDEYKTPAALAKPMRDSGSDPTPVTRYSCPYVGCKRNKEHRKFQPLKTILCVKNHYKRSHCDKRYTCSRCNTKKFSVIADLKTHEKHCGRDKWLCSCGTTFSRKNKLFGHVALFQGHTPALPMDDIKATGASEQPQGSEAMDDMVGSTGYNFPGSTSDGIPNLDMKVADDTRGYFSPLNFDPCFGALDDFARPGFDISENPFSFLPSGPGSCSFGQLSGDS
> TaSTOP1D_VilosoMole
MKASSSMASDASGNTEPGQQGVRFSSMDQSCFARPGQSIPGYPPFFGPQSSNFYLPDDSVAKACDLFEPNPPQNNPVADWDPQAMLSNLTFLEQKIKQVKDIVQSMGNRGSQDAGGSCELAAKQQLVTADLTSIIIQLISTAGSMLPSMKTPLLSSNPAVRQLNTPGSPMGFGSIVNQRPSTIREEVVNDISKTSDYEELMNTLNTAHDEKDDLIKCPNPCVGEGPEPVPMEDHDVKESDDGGEAEHLPPGSYVVLQLEKEEILAPHTHFCVICGKGFKRDANLRMHMRGHGDEYKTPAALAKPMRDSGSDPTPVTRYSCPYVGCKRNKEHRKFQPLKTILCVKNHYKRSHCDKRYTCSRCNTKKFSVIADLKTHEKHCGRDKWLCSCGTTFSRKDKLFGHVALFQGHTPALPMDDIKASGALEQPQGSEAMDDMVASTGYNFPGSTSDGIPNLDMKVADDTRGYFSPLNFDPCFGALDDFARPGFDISENPFSFLPSGPGSCSFGQISGDS
> TaSTOP1A_Saloio
MKASSSMASDASGNTDPGQQGARFSSMDQSCFARPGQSIPGYPPFFGPQSSNFYLPDDSVAKACDPFEPNPPQNNPVADWDPQAMLSNLTFLEQKIKQVKDIVQSMGNRGSQDVGGSCELAAKQQLVTADLTSIIIQLISTAGSMLPSMETPLLSSNPAVRQLNTPGSPMGFGSIVNQRPSTVREEMVPDITKTPDYEDLMNTLNPAHDEKDDLIKCPNPCVGEGPEPVPMEDHDVKESDDGGEAEHLPPGSYVVLQLEKEEILAPHTHFCVICGKGFKRDANLRMHMRGHGDEYKTPAALAKPMRDSVSDPTPVTRYSCPYVGCKRNKEHRKFQPLKTILCVKNHYKRSHCDKRYTCSRCNTKKFSVIADLKTHEKHCGRDKWLCSCGTTFSRKDKLFGHVALFQGHTPALPMDDIKATGASEQRSEAMDDMVGSTGYNFPGSTSDGIPNLDMKVADDTRGYFSPLNFDPCFGALDDFARPGFDISENPFSFLPSGPGSCSFGQLSGDS
> TaSTOP1B_Saloio
MKASSSMASDASGNTEPGQQGVRFSSMDQSCFARPGQSIPGYPPFFGPQSSNFYLPDDSVAKACDPFEPNPPQSNPVADWDPQAMLSNLTFLEQKIKQVKDIVQSMGNRGSQDVGGSCELAAKQQLVTADLTSIIIQLISTAGSMLPSMKTPLLSSNPAVRQLNTPGSPMGFGSIVNQRPSTVREEMVPDISKTSDYEELMNTLNTAHDEKDDLIKCPNPCVGEGPEPVPMEDHDVKESDDGGEAEHLPPGSYVVLQLEKEEILAPHTHFCVICGKGFKRDANLRMHMRGHGDEYKTPAALAKPMRDSGSDPTPVTRYSCPYVGCKRNKEHRKFQPLKTILCVKNHYKRSHCDKRYTCSRCNTKKFSVIADLKTHEKHCGRDKWLCSCGTTFSRKDKLFGHVALFQGHTPALPMDDIKATGASEQPQGSEAMGDMVGSTGYNFPGSTSDGIPNLDMKVADDTRGYFSPLNFDPCFGALDDFARPGFDISENPFSFLPSGPGSCSFGQLSGDS
> TaSTOP1D_Saloio
MKASSSMASDASGNTEPGQQGVRFSSMDQSCFARPGQSIPGYPPFFGPQSSNFYLPDDSVAKACDLFEPNPPQNNPVADWDPQAMLSNLTFLEQKIKQVKDIVQSMGNRGSQDAGGSCELAAKQQLVTADLTSIIIQLISTAGSMLPSMKTPLLSSNPAVRQLNTPGSPMGFGSIVNQRPSTIREEVVNDISKTSDYEELMNTLNTAHDEKDDLIKCPNPCVGEGPEPVPMEDHDVKESDDGGEAEHLPPGSYVVLQLEKEEILAPHTHFCVICGKGFKRDANLRMHMRGHGDEYKTPAALAKPMRDSGSDPTPVTRYSCPYVGCKRNKEHRKFQPLKTILCVKNHYKRSHCDKRYTCSRCNTKKFSVIADLKTHEKHCGRDKWLCSCGTTFSRKDKLFGHVALFQGHTPALPMDDIKASGALEQPQGSEAMDDMVASTGYNFPGSTSDGIPNLDMKVADDTRGYFSPLNFDPCFGALDDFARPGFDISENPFSFLPSGPGSCSFGQLSGDS
>TaSTOP1D_Ruivo
MKASSSMASDASGNTEPGQQGVRFSSMDQSCFARPGQSIPGYPPFFGPQSSNFYLPDDSVAKACDLFEPNPPQNNPVADWDPQAMLSNLTFLEQKIKQVKDIVQSMGNRGSQDAGGSCELAAKQQLVTADLTSIIIQLISTAGSMLPSMKTPLLSSNPAVRQLNTPGSPMGFGSIVNQRPSTIREEVVNDISKTSDYEELMNTLNTAHDEKDDLIKCPNPCVGEGPEPVPMEDHDVKESDDGGEAEHLPPGSYVVLQLEKEEILAPHTHFCVICGKGFKRDANLRMHMRGHGDEYKTPAALAKPMRDSGSDPTPVTRYSCPYVGCKRNKEHRKFQPLKTILCVKNHYKRSHCDKRYTCSRCNTKKFSVIADLKTHEKHCGRDKWLCSCGTTFSRKDKLFGHVALFQGHTPALPMDDIKASGALEQPQGSEAMDDMVASTGYNFPGSTSDGIPNLDMKVADDTRGYFSPLNFDPCFGALDDFARPGFDISENPFSFLPSGPGSCSFGQLSGDS
