# Supplementary material for: Molecular characterization of TaSTOP1 homoeologues and their response to aluminium and proton (H+) toxicity in bread wheat (Triticum aestivum L.)
Source: BMC Plant Biol. 2013 Sep 13;13:134. doi: 10.1186/1471-2229-13-134 (PMC3848728; doi:10.1186/1471-2229-13-134)
Supplement: Additional file 7 — Detail of STOP like proteins in different plant species used for phylogenetic analysis. [file 1471-2229-13-134-S7.rtf]

Additional File: 7. Detail of STOP like proteins in different species used for phylogenetic analysis.

S. No.	Scientific Name	Sequence name and Protein ID	Amino acid length	
1	Manihot esculenta	M.esculenta_cassava4.1_005470m	525	
2	Ricinus communis	R.communis_29728.m000841	357	
		R.communis_29589.m001269	416	
3	Linum usitatissimum	L.usitatissimum_Lus10009435	469	
4	Populus trichocarpa	P.trichocarpa_ POPTR_0013s11900	509	
		P.trichocarpa_ POPTR_0019s11520	506	
5	Phaseolus vulgaris	P.vulgaris_Phvulv091023250m	512	
6	Glycine max	G.max_Glyma10g35940	507	
		G. max_ XP_003539439.1	410	
7	Cucumis sativus	C.sativus_Cucsa.079940	508	
		C. sativus _XP_004173281.1	381	
8	Prunus persica	P.persica_ppa004510m	505	
		P. persica_ EMJ07676.1	388	
9	Malus domestica	M.domestica_MDP0000453148	527	
10	Arabidopsis thaliana	A.thaliana_AT1G34370	499	
		A.thaliana_AT5G22890	373	
11	Capsella rubella	C.rubella_Carubv10008876m	514	
		C. rubella_ EOA22034.1	369	
12	Brassica rapa	B.rapa_Bra036750	491	
13	Thellungiella halophila	T.halophila_Thhalv10007411m	506	
14	Carica papaya	C.papaya_evm.model.supercontig_66.56	466	
15	Citrus sinensis	C.sinensis_orange1.1g009831m.g	524	
16	Eucalyptus grandis	E.grandis_Eucgr.F02627	528	
17	Vitis vinifera	V.vinifera_GSVIVT01034505001	527	
		V. vinifera_ XP_002272574.1	423	
18	Mimulus guttatus	M.guttatus_mgv1a007605m	402	
19	Aquilegia coerulea	A.coerulea_Aquca_017_00778	535	
20	Sorghum bicolor	S.bicolor_Sb03g041170	519	
		S. bicolor_ XP_002444574.1	429	
21	Zea mays	Z.mays_GRMZM2G068710	467	
		Z.mays_GRMZM2G075956	519	
22	Setaria italica	S.italica_Si001061m	517	
		S.italic_Si010000m.g	477	
23	Oryza sativa	O.sativa_Os01g65080	522	
		O.  sativa_Os08g0562300	385	
24	Brachypodium distachyon	B.distachyon_Bradi2g56320	525	
		B.distachyon_ XP_003571264.1	387	
25	Triticum aestivum
Cv Barbela 7/72/92	>T.aestivum_genome_A	510	
		>T.aestivum_genome_B	512	
		>T.aestivum_genome_D	512	
26	Solanum  lycopersicum	Solanum  lycopersicum AK320912.1	513	
		S.  lycopersicum_ XP_004250985.1	384	
27	Hordeum vulgare  	Hordeum vulgare  AK252406.1	512	
28	Musa acuminata	M.acuminata_Achr5P01170_001	393	
		M.acuminata_Achr10P15970_001	398	
		M.acuminata_Achr6P18840_001	441	
29	Triticum urartu	T. urartu_EMS66742.1	506	
		T. urartu_EMS47263.1	330	
30	Aegilops tauschii	Ae. tauschii_EMT26835.1	506	
31	Arabidopsis lyrata	A.lyriata XP_002874102.1  	368	
32	Fragaria vesca subsp. Vesca	F. vesca_ XP_004294539.1	402	
		F. vesca_ XP_004290434.1	402	

>M.esculenta_cassava4.1_005470m
MDHKDRLCQETWANSSSSGNELPKNISTQKPSYANFNFNAQQHQQKWEDTSIFGVRTNSTFQEFNPQSETQSLLPSNNNDHIKIPDRESSRINETLQTNKVPDWDPKAMLNNLSFLEQKIHQLQDLAHLIIGRRSQVLGRPDELLTQQQQLITADLTSIIVQLISTAGSLLPSVKHTLSVAMPPAGQLGGVLFPSGAGMNSSLQPQYSSASKVSDQSNQMDIAVNCGTEQNYSIEEHEMRDEEDAEEGENLPPGSYEILQLEKEEILAPHTHFCTICGKGFKRDANLRMHMRGHGDEYKTPAALAKPNKEASSEPVLIKRYSCPFAGCKRNKDHKKFQPLKTILCVKNHYKRTHCDKSYTCSRCNTKKFSVIADLKTHEKHCGKDKWLCSCGTTFSRKDKLFGHIALFQGHTPAIPPDETKATPGPSDKGDENEVANSVGSMNYSFGSGGANGSGAQDIMDVKGGVDDPASYFSPLNFDTCNFGGFHEFPRPPFDDSESSFSFLISGLCNYSQKTGGESSSNNIH
>R.communis_29728.m000841
MDRKERLCTETWANSSSSGSGLPEHLSTEKPSFANFSSQQDQQKWEDTAIYGIRTDPPFQEFNLPSETQTLLPSNPDDQIKIMDEEDSHINEALQINKIPDWDPKAMLSNLSFLEQKIHQLQDLVHLIVGRRGQILERPDDLVAQQQQLITADLTSIIVQLISTAGSLLPSVKHTLFAATPGGQLGHLGEILFPSGMGMNSIVQPQHSSGNKLSHHSNQMDISGNCGTEQNYSVDEHELKDEEDVEEGENLPPGSYEILQLEKEEILAPHTHFCTICGKGFKRDANLRMHMRGHGDEYKTPAALAKPNKESSSEPVLKRYSCPFAGCKRNKDHKNYNLGIGYLSPPLNNLKINKMAI
>R.communis_29589.m001269
MIAGLSSRNFSNHHQQQHHHVLQVYPAASDDIVSTSLEASSSSEIGHANSLLYSLSVVKDKVNQVQSLVSILTSPDHHHQSYQQQNPEPNSIANLGTIIQEIIVTASSMMFTCQQMAIGSSNSINNNASELHQPAPPQPKVAAPQENVLLPNSQDHQRGQSFFASENFDWYDNNNYSNNSCNGTENNQTASGMHNVISINTNKVERKELSENFSDVLQEGNNKSIKSYDIIELDASYLLAKYTHYCQVCGKGFKRDANLRMHMRAHGDEYKTSAALSNPMKNINTSAIGDSSEDSVMKLPRKYSCPQEGCRWNQKHAKFQPLKSMICVKNHYKRSHCPKMYVCKRCNRKQFSVLSDLRTHEKHCGDLKWLCCCGTTFSRKDKLMGHVALFVGHTPAITSFNNPAKVESHQMQLDDR
>L.usitatissimum_Lus10009435
MDPKEVLCGEPWAKPSSSNDPDPKISLQNVPSLMDYGFRIDQPYQDRTENNNNKAPDWDPRAMLNNLSFLEQKIHQLQGVVHLIIGRKDQEVPPLGNRPDELVTQQQQQLVTADLTSIIVQLISTAGSLLPSVKHTLAATNPIGQFEQFGLIQPKPPTGTAPVPDQTDLMDLATNYSTEEHEMKDEEDTEDGENLPPGSYEILQLEKEEILAPHTHFCTICGKGFKRDANLRMHMRGHGDEYKTPAALAKPPKEATSSEPTGAEPAMIKRYSCPFAGCKRNKDHKKFQPLKTILCVKNHYKRTHCDKSYICSRCHTKKFSVIADLKTHEKHCGRDKWLCSCGTTFSRKDKLFGHIALFQGHTPAIPPDETKALFEKGEDSNNIEAAAAANNKVGIGALDFNCLGSNSNIMDFKSVVAADDTAAATACFSPLSFDSFQEFNRQPFDDSDSSFSFLLSGSCNYSQKSCGDS
>P.trichocarpa_POPTR_0013s11900
MEPKDRPSLDAWANPSSSSNGLPRKMALENPSFTNFNLQQQEQKWDDTSILDYGIRIEPPFREFNQASESEYLLSSNCNNQPKVLDQEDGQNNEALQTSKLQDWDPRSMLNNLSFLEQKIHHLQDLVHLIVGRKGQALGGQDQLVTQQQQLITADLTSIIVQLISTAGSLLPSVKHTFSTGTPNGQLGQLGGILFPPLAGMNCVPQPQHGSGSKVSDQCNQMDVTGNCGTEPNHSIEEHEMKEEEDADEGENLPPGSYDILQLEKEEILAPHTHFCTICGKGFKRDANLRMHMRGHGDEYKTPAALAKPNKEPSSEPVIIKRYSCPFAGCKRNKDHKKFQPLKTILCVKNHYKRTHCDKSYICSRCNTKKFSVMADLKTHEKHCGKDKWLCSCGTTFSRKDKLFGHIALFQGHTPAIPLEETKGPYVSCDKVDGNEASNKVGNTNFSLGSNAGSGSGAQNVIEVKEDADDPASYFSPLSFDTCNFGGFHEFPRPPFDDSESSFSFLEAP
>P.trichocarpa_POPTR_0019s11520
MDLTDRLSVDVWADTSSSGNELPRRMSSENPSFTDFNLQQQQKQQQKWEDNIISPPFGGFNQASESGYLLLSNRNNQTKILDHQEDGKSIETLETNKVQDWDPRAMLSNLSFLEQKIHHLQDLVHLFVGRKGQGLGGQDQLVTQQQQFLITADLTSIIVQLISTAGSLLPSVKHTLSTDTPNGHLGQLGGLLFPPVAGMNCSPKPQHGSGRRVSDQSDKMDGTGNCGTDQNHFIEEHETKEEEDADEGDYLPPGSYDILQLEKEEILAPHTHFCTICGKGFKRDANLRMHMRGHGDEYKTPAALAKPNKESSSDPVVIKRYSCPFSGCKRNKDHKKFQPLKSILCVKNHYKRTHCDKSYTCSRCNTKKFSVTADLKTHEKHCGKDRWLCSCGTTFSRKDKLFGHIALFQGHTPAIPLEETKGPAVSSDKVDGNEASNKVGNINFSFGSHAATGSGAKNVMEVNDDADDPSSCFSPLNFDTCNFGGFHEFPRPPFDDSESSFSFLLS
>P.vulgaris_Phvulv091023250m
MDLEGGLCANNCTRSSSLTSPGNGLQTNLSSDPPSFYGLEIESPFNTFNPPSPPIQSVLPGQSNTDIEIPDQENCPLSDSSNTTKLQDWNPSAMLNNLCFLEEKIHQLRDLVHLIVNKKCQPFGQSHELVTQEQQLVTADLTSIIVQLISTAGNLLPSVRHTLTNTNPLVGQMDQLHGINLPFGSEPSSGIRPQNSSGNKLFGKSTPNDRPNPCEMEQNYNMEEHEPKDEEDVDEGESLPPGSYEILQLEKEEILAPHTHFCTICGKGFKRDANLRMHMRGHGDEYKTPAALAKPHKETGSEPKLIKRYSCPYAGCKRNKDHKKFLPLKTILCVKNHYKRTHCDKSYTCSRCNMKKFSVMADLKTHEKHCGKDKWLCSCGTTFSRKDKLFGHIALFQGHTPAIPLDDIKRVAEPPDPQNRECSNKVGSVNFCFGSKLSSENGVQNMMDMKGNIDDPMNYFSSLNFEGCNFGAFNEFPQTPFEDSEGSFSFLMSGSFNYAPKFGGGESSTDNL
>G.max_Glyma10g35940
MDPKGSLCAKNCTRTSSLTSPGNGLQTNLSSDPPSFYGVRIESPFKEFIQPSPPTQSVLPGESNADIDIPDQENCPLSNSSHTSKLQDWNPSAMLNNLSFLEEKIHQLQDLVHVIVNKKCQPFGQPHELVTQEQQLITADLTSIIVQLISTAGSLLPSVRHTLTNTNPLVGQLDQLHGINLPFGSEPSSGIRPQNNSGNKLFDQSTQNDLPNKLEMEQNYNMEEHEPKDEEDADEGENLPPGSYEILQLEKEEILAPHTHFCTICGKGFKRDANLRMHMRGHGDKYKTPAALAKPHKETGSEPKLIKRYSCPYAGCKRNKDHKKFQPLKTILCVKNHYKRTHCDKSYTCSRCNTKKFSVMADLKTHEKHCGKDKWLCSCGTTFSRKDKLFGHIALFQGHTPAIPLDDTKGVAEPPDIQNRESNNKVESINFCFGSNPSTENVVQNIMDMKGNIDDPMNYFSSLNFEGCNFGAFNEFSQPPFEDSEASFSFPMSGSYNYAPKFDSDNL
> G.max_ XP_003539439.1
MISRATSSFQSVHHQGLQMFPDAEDFVSPSVESGSGTSHSSSLLFNLSILKDKLSEVQTLVGVILSPNQSLPESTSMAISSMNSTIQEIVVTATSMMFTCQQMALTAPPGTNNNTNKLHHHQNKSLPHSSNINFATNGSCVLGNNIGTDNISDHHGLFSSTESETLDWFSESYNNNDDNSNSNIISKVAISENNIITRGGGGGGVLSPRDEPNEEGLSPKMNSDDIIIELDAANLLAKYTHYCQVCGKGFKRDANLRMHMRAHGDEYKTNAALSNPIKNQRDLECLMSVKPKRYSCPQEGCRWNQRHAKFQPLKSMICAKNHYKRSHCPKMYVCKRCNQKQFSVLSDLRTHEKHCGDLKWLCSCGTSFSRKDKLMGHVALFVGHQPAINNNGLSYSGKLIRAAPHIANAD 
>C.sativus_Cucsa.079940
MEPEDSLCSSEVWTKSSSFPNGNVQQPPQKWPDTSILDYAMDQPFQEFQEQTESKASLTCNPNQQIEILDKDSNQMNAALLASKIQDWDPRAMLNNLSFLEQKIHQLQELVHLIVGRRGQVFGRPDELVVQQQQLITADLTSIIVQLISTAGSLLPSVKHNLSAAVPPVGQLEPFDKVIFASGPGTNGGVQSQHGDGTKLPELPTQVDGSSKCGKEQNMTVEEHESKDEEDADEHENLPPGSYEILQLEKEEILAPHTHFCAICGKGFKRDANLRMHMRGHGDEYKTAAALAKPNKELGSETMLIKRYSCPFTGCKRNKDHKKFQPLKTILCVKNHYKRTHCDKSFTCSKCNSKKFSVIADLKTHEKHCGKDKWLCSCGTTFSRKDKLFGHIALFQGHTPAIPLDESKGATVSCDRGERYETTNKLGSINFSFGSIASGGSSVETIADAKGGVDDPLSYFSPLNFDSCNFGGFHEFPRPPFENTDNAFSFLIPGSGNYTQKIWCRIKL
> C.sativus_ XP_004173281.1
MISNSQMLSLPSQHPLVSSSNDHHETLDNLLSSYSQSSNTLLYNLSVLKEKLHQIQSVVNIAVYNLQNSTESLPSAPPASSQSPVATAAVNSLIQELIMAASSMLFTCQQMDNLAVNASLHNNNVGNNLQGQHHQHQHQQQGPVDDNNGRSNTLFNMANDHHHQRQDWYNTTTSNNYNKDNNNSRTIMTTKTTQDHRNPINIVELDASDLLAKYTHYCQICGKGFKRDANLRMHMRAHGDEYKASGALSNPEKSHRKDLSNISKMGIKYSCPQEGCRWNQKHVKFQPLKSLICVKNHFKRTHCPKMYVCKLCSRKKFSVLSDLRTHEKHCGDVKWLCSCGTTFSRKDKLMGHVALFVGHTPAMGSSTKFLGKQEHEYVHSF
>P.persica_ppa004510m
MDHKEKLCADAWENPSSGNDLTGKISSDCPPFAIFKPQLHQQKWEDPSILDYGGIRIEPSFQEFNQPSETQTSLPCNSNNDTKMADREDGRMCETPQANKSQDWDPRTMLNNLSYLEQKIHQLQDLVHLIVGRRGQVLGRPDELVAQQQQLITADLTSIIAQLISTAGSLLPSVKHTLSTTTPSIGQFGQLGGSFVPSGVGTDASVKLQNSNGSKLADQSNQTDLISNYGTEQNYSVEEHESKDEEDADEGENLPPGSYEILQLEKEEILAPHTHFCAICGKGFKRDANLRMHMRGHGDEYKTPAALAKPNKESSSEPTLIKRYSCPYAGCKRNKDHKKFQPLKTILCVKNHYKRTHCDKSYTCSRCNTKKFSVIADLKTHEKHCGKDKWLCSCGTTFSRKDKLFGHIALFQGHTPAIPLDETKGTQGPADHGEGIQNLMDVKESINDPTSYFSPLNFETCNFDGFHEFPRPPFEDSESSFSFLMPGSCNYTHKTGGEPNSNNLE
>P.persica_EMJ07676.1
MTEDPQVSSFSTSSGPENHSTSLLYSLSVLKEKVRQAKSLFSILISPDHHHHQSQPPESTSMAIASMNNVIQEIIVTASSMMFTCQQMALVSPPPPPPPPGNNNPTSTSNTIDHEEQLLHHQGLNKPPPPRFSQPNFGSGLDNKIETPFYPTNDDQALDWFSDSYIDCTTRDTKTRHVQGKQQPISPKSFDIIELDAADLLAKYTHYCQVCGKGFKRDANLRMHMRAHGDEYKTTVALSNPMKKNNSSTNNNNNHGAGGTDGNTDCSTNLPRKYSCPQVGCRWNQKHAKFQPLKSMICVKNHYKRSHCPKMYVCKRCNRKQFSVLSDLRTHEKHCGDLRWQCSCGTTFSRKDKLMGHVALFTGHTPVVISSLASRLGKADQRSHGSQM 
>M.domestica_MDP0000453148
MDPKERQWDTWENPSTGNDVTNTISSDHPSFTNFNSQQHQREWERPSVLDYEMRMEPSFLKFHQPSDSQMSYTCNSKNDTKIPDQEGGKMHEVQQPNKIQDWDARMTLNNLTFLEQKIHQLQDLVHVIVGRRGQVLGRPDELVAQQQQLITADLTSIIAQLISTAGSLLPSVKHTLSTTLPSTGQFGQLGGSFIPSAAGNDAGVKMQINSGSKLADQANQTDLISNYGTEHIEEHETKDEEDADEGENLPPGSYEILQLEKEEILAPHTHFCAICGKGFKRDANLRMHMRGHGDEYKTAAALAKPNKESSSEPTLIKRYSCPYAGCKRNKDHKKFQPLKTILCVKNHYKRTHCDKSYTCSRCNTKKFSVIADLKTHEKHCGIDKWLCSCGTTFSRKDKLFGHITLFQGHTPAIPLDETKGTLGPADHGEGSEASNRVGSISFSVSSTAPGGGGAAQSLMDVKESIDDPTSYFSPLNFETCNFDGFQEFPRPPFEDTESSFSFLMPGSCNYTHKTGGGESNFNNLHRQ
>A.thaliana_AT1G34370
METEDDLCNTNWGSSSSKSREPGSSDCGNSTFAGFTSQQKWEDASILDYEMGVEPGLQESIQANVDFLQGVRAQAWDPRTMLSNLSFMEQKIHQLQDLVHLLVGRGGQLQGRQDELAAQQQQLITTDLTSIIIQLISTAGSLLPSVKHNMSTAPGPFTGQPGSAVFPYVREANNVASQSQNNNNCGAREFDLPKPVLVDEREGHVVEEHEMKDEDDVEEGENLPPGSYEILQLEKEEILAPHTHFCTICGKGFKRDANLRMHMRGHGDEYKTAAALAKPNKESVPGSEPMLIKRYSCPFLGCKRNKEHKKFQPLKTILCVKNHYKRTHCDKSFTCSRCHTKKFSVIADLKTHEKHCGKNKWLCSCGTTFSRKDKLFGHIALFQGHTPAIPLEETKPSASTSTQRGSSEGGNNNQGMVGFNLGSASNANQETTQPGMTDGRICFEESFSPMNFDTCNFGGFHEFPRLMFDDSESSFQMLIANACGFSPRNVGESVSDTSL
>A.thaliana_AT5G22890
MHIHMMNRDEHIAKKVEGSISSFSGETSTSSKQIYVNPVTTTGTKSMEDDDVSLSLLYNLSTLHEKVHQIQSLVSFYMVSTNNINQSSGSTSLAVANIGSLVQEIITAASSMLYTCQQLQIGSNNNNNDIDNDQTVDAMVLEFSRQETDPGHDFVQESTNLFGVQERGQISFPDQNLDWYNTETINPKKDKHRSKPSSGSYDILELDVADLLAKYTHYCQICGKGFKRDANLRMHMRAHGDEYKTREALISPTSQDKKGGYSLKKHYYSCPQHGCRWNQRHEKFQPLKSVICAKNHYKRSHCPKMYMCRRCSVKHFSVLSDLRTHEKHCGDIKWVCSCGTKFSRKDKLMSHVSLFLGHVPAHGSSKPPTITLK
>C.rubella_Carubv10008876m
METEDDLCQQTNWGSSSSKLQEQGSSDGGGNQAFSGFTSEQKWEEASILDYEMDGVEPDLQVFDEMKASGEENVQANVDFLQGVRAQAWDPSTMLSNLSFMEQKIHELQELVHLLVGRGEQVQGCQDELAAQQQQQLITTDLTSIIIQLISTAGSLLPSVKHNMSTAPGPYTGQPGSAMFPCAREANNVASQSQNNNNCGAQEFDLPKAVVVDERESHVVEEHEMKDEDDAEEGENLPPGSYEILQLEKEEILAPHTHFCTICGKGFKRDANLRMHMRGHGDEYKTAAALAKPNKESIPGSEPMLIKRYSCPFLGCKRNKEHRKFQPLKTILCVKNHYKRTHCDKSFTCSRCHTKKFSVIADLKTHEKHCGKNKWLCSCGTTFSRKDKLFGHIALFQGHTPAIPIEETKPSASSSTQRENSEGGNNNQGMVGFNLGSPSNANQETAQFGMMDGNISFEDSFSPMNFETCSFGGFHDFPRLMFDDSDSSFQTLIANTCGFSSPRNVGESVLDTSI
> C.rubella_ EOA22034.1 
MMNQDDHLTSKGEGSMSSFSGTTATSSKQNYVNNTVTTAGVKSLEDDDASLSLLYNLSTLQDKVHQTQSLVSFYMISTNNINQSSGSTSLAVANIGSLVQEIITAASSMLFTCQQLNIGSNNNNDNDQTADHAMVLEFSRQETEPGHNFVQESTNLFGVQERGQISFPDQNLEWFGTQTLNPKKEYHRSKQRSGNYEILELDVADLLAKYTHYCQICGKGFKRDANLRMHMRAHGDEYKTREALISPISQDKKGDYSLKKHYYSCPQQGCRWNQRHEKFQPLKSVICAKNHYKRSHCPKMYMCRRCSVKHFSVLSDLRTHEKHCGDIKWVCSCGTRFSRKDKLMSHVSLFLGHVPAHGSSAPTTTIKRS
>B.rapa_Bra036750
MEPEDDLCQNNWGGSSSSSSSKRREQVCFTSQHKWEDASILDYEMGMEEEPAFQENSNNNNGGQVNVDFLQGVRAQAWDPRTMLSNLSFMEEKIHELQDLVHLMVARNGQLQGRQEQLVAQQQQLITTDLTSIIIQLISTAGSLLPSVKHHNMSTAPGPFTGSALFPYPREANNLASQTLNNNNNNTCEFDLPKPIVVEEEHEMKDEDDVEEGENLLPGSYEILQLEKEEILAPHTHFCTICGKGFKRDANLRMHMRGHGDEYKTPAALAKPNKEAVPGSEPMLIKRYSCPFPGCKRNKDHKKFQPLKTILCVKNHYKRTHCDKSFTRSRCHTKKFSVIADLKTHEKHCGKNKWLCSCGTTFSRKDKLFGHIALFQGHTPAIPLEETKPSAQKGSSACENSNNNNTGMVGFNLGSATNAIEEVAQPGFMDGKIRFEDSFSPLSFDTCNFGGFHEFPRPMFDDSESSFQMLISSACGFSPRNGGESVSNTSL
>T.halophila_Thhalv10007411m
METEDDMCQNNWGSLSSSSKLEDGNQAFAGFTSQQKWEDAEILDYEMGIEKNLQAFDQMKPSSGQDNDNNNKNNNSTQANVDFLQGVKAQAWDPRTMLSNLSFMEQKIHELQDLVHLIVGRNGLVAQQQQQLITTDLTSIIIQLISTAGSLLPSVKHNMSSTAPGPYTGSAVFPCPREANNVASQSQNSNHQRGATQDFDLPKPIVVDERDSHVVEEHEMKDEDDAEEGVENLPPGSYEILQLEKEEILAPHTHFCTICGKGFKRDANLRMHMRGHGDEYKTPAALAKPNKEAVPGTEPMLIKRYSCPFPGCKRNKDHKKFQALKTILCVKNHYKRTHCDKSFTCSRCHTKKFSVIADLKTHEKHCGKNKWLCSCGTTFSRKDKLFGHIALFQGHTPAIPLEETKPSAQRGSSEGENNNTGMVGFNLGSATNADQETEQPGFMEEKISFEESFSPLSFDTCNFGGFHEFPRPMFDDSESSFQMLISSACGFSPRNGGGGSVSNTIL
>C.papaya_evm.model.supercontig_66.56
MDTKERLCPDNWGNSSSSGNELYKKVSSDHRSFTGFGLQQNHQKWEDPSFLEYAMRIEPPFQEFNQPSQTQFSLCNQNDQMRILVQENGQENEMLESNKIQDWDPRATLNNLSFLEQKIHQLQDLVHLIVGRRGQTLGHPDQLITQQQHLITADLTSIIVQLISTAGSLLPSARHSLSAANPSGAQIGQLGGVLFPHARGLHGEMQTQNNCGSKVSDSSTKIDPPTNYQVDENHIVEEHELKEEEDVDEGESLPPGSYEILQLEKDEILAPHTHFCTICGKGFKRDANLRMHMRGHGDEYKTAAALAKPVKESSSEPILIKRYSCPFPGCKRNKDHKKFQPLKTILCVKNHYKRTHCDKSFLCSRCNTKKFSVIADLKTHEKHCGKDKWLCSCGTTFSRKDKLFGHIALFQGHTPAIPLDETKTSAGPSGRGENNEARYKVGSINFNFASNTPVLAVSTIMDVKGV
>C.sinensis_orange1.1g009831m.g
MDLKEGLRAETWAKSSLAENEMSKRISSNNPCFTDFNSQQYQQKWEDPSILDYDIRINPAFQEFNQPPQNQSSLPCDPTNQIKNPFQMNENLQTNKMQDWDPKAMLNNLSFLEQKIHELQDLVHLIVGRRSQILGRPDELVAQQQQLITADLTSIIVQLISTAGSLLPSMKHTLSAATPSMGQLGQFGGVPFPPGAGLSDSIQVQNSCAKKVSNQSNPIDLVGNSGTQQNHAVEEHDLKDEDDADDGEQLPPGSYEILQLEKEEILAPHTHFCTICGKGFKRDANLRMHMRGHGDEYKTPAALAKPHKESSSEPMLIKRYSCPYAGCKRNKDHKKFQPLKTILCVKNHYKRTHCDKSYVCSRCNTKKFSVIADLKTHEKHCGKDKWLCSCGTTFSRKDKLFGHIALFQGHTPAIPLDETIKGLAGPSDRREGNEATSKIGSTNFNFGSSVPNGTGVSDMDAKGNVDDPTNYFSPLNFDTCNFDGFHEFPRPPFDDSENAFSFLIPGSCNYIQKTGGETSLNTLK
>E.grandis_Eucgr.F02627
MDLKMDLEERLRAETWGKPSSVNDLPQRVPPDRQTQFPNFASHKNLQKREDQDPSISNYGMRIEPSFSEFNRPSECQPPLPSNPISQDRGVQMNDILQLGKTQEWDPKAMLNNLSFLEQKIHQLQELVHAIVGRRGPVLGRPDELVAQQQQLITADLTSIIIQLISTAGSLLPSVKNSLSSASTPPIRQLGQLGGILNNSGSGIGLDSNLVLPSQGGSKVPDQSNQVDPMDQSAIDNLEDHESKDDEDGDEGENLPPGSFEILQLEKEEILAPHTHFCTICGKGFKRDANLRMHMRGHGDEYKTPAALAKPHKEAGSEMMLIKRYSCPYAGCKRNKDHKKFQPLKTILCVKNHYKRTHCDKSYTCSRCNTKKFSVIADLKTHEKHCGKDKWLCSCGTTFSRKDKLFGHITLFQGHTPAIPFDENKGGLSLQGEHNEDTNKVGNVSFSFGSSTPSSGGVQNIMEDVKGNVDDPSSFFSPLSFEASNFGGFNEFTRSAFDDSEGAFSFLLQASCNYTQKNGGQSSSNNLE
>V.vinifera_GSVIVT01034505001
MDLKDRLSVDTWTQSSSSGNELPKDQQSFTNFSLQQQQHQHKWEDPSVLDYSVRIEPAFQEFNQTSATSSSLPCNPNSQIKNLNREDCQMNEMLEPNRIQDWDPRVMLSNLSFLEQKIHQLQDLVHLIVGRRSQVLGRTNELVAQQQQLVTADLTSIIVQLITTAGTLLPSVKNTLSTASPSVGQLGQLGGVLFPSGTGMNGGGVAQSSGGSKVSDQSNQIDLTGACVIEQNNATEEHELKDEDDADEGENLPPGSYEILQLEKEEILAPHTHFCMICGKGFKRDANLRMHMRGHGDEYKTPAALAKPNKESSSEPVLIKRYSCPFAGCKRNKDHKKFQPLKTILCVKNHYKRTHCDKSYTCSRCNTKKFSVIADLKTHEKHCGKDKWLCSCGTTFSRKDKLFGHIALFQGHTPAIPLDETKGSVGPSDRGEGNGAANKVGSVGFNFSSNASSGSGVQDMMMDVKRGADEPTGFFSPLTFDPCSLVGFHEFPRPPFEDSESSFSFLVPGSCSYTRKTGGESSSNDLE
> V.vinifera_ XP_002272574.1
MIPVGTSNCFVNGSQGLQMYGMTAEGSVSSSLGGSSVLEAHSCSLVYSFSILKDKVHQVQSLISIFVPPNQGQPESMAMAVAGMGNLIQEVIAAASSMMYSCQQMGYGAAPGNSGTNHGLPQQGVELSDGRVCGDTGVVQMGEERGQGFYSSDQSLDWYGDNHNNSNTNDHSRTIIVSNNDKVESRELPQGSTQMNEGLGGVLPKTFDIVELDAEDLLAKYTHYCQICGKGFKRDANLRMHMRAHGDEYKSNAALSNPTKNIGREMENKDDLIKLPRKYSCPQEGCRWNRKHAKFQPLKSMICVKNHYKRSHCPKMYICKRCNQKQFSVLSDLRTHEKHCGDLKWLCSCGTTFSRKDKLMGHVALFVGHTPAINSMSKPPSVSLSKSWTDLLSPILAHELAQTWPMTGSKAARLELGQPGPFT
>M.guttatus_mgv1a007605m
MSDFQDEQIHGSHESNNMQDFDTKAMLNNLSFLEQKIHQLQEMVHLIVRQRGQADAILVQQQQLITADLTSVIVQLISTAGSLLPSLKTTLSSENPIGQFVANHHSAKVSDNNNNNNVVSHKIEDHSDHEMMKSDEDADEGENLPPGSYEILQLEKEEILAPHTHFCTICGKGFKRDANLRMHMRGHGDEYKTPAALAKPNRESISDHPILVKRYSCPYIGCKRNKDHKKFQPLKTILCVKNHYKRTHCDKNYTCSRCNTKKFSVIADLKTHEKHCGRDKWLCSCGTTFSRKDKLFGHIALFQGHTPAIPVEESKGFSGPPDRGQCSGVDQPEVNFKFDTPSTSACRNVIMDDDPTSYFSPLDYEFPRSTFEDSESSFSFMLSESCDYLPKNGIYNGLNDME
>A.coerulea_Aquca_017_00778
MNPAVNLCSDTWAECSVRNKSSSEKFHSQQHHRKREDASSPDFTPPLSKFCKPSEPTSSHSGNLDNQRIIHDIVDGQAKDKSATAKTNEWNPNSILSNLSFVEQKIHQLQDLVHLMIGQDKRIVNLSDELVAQQQQLVTADLTSIIVQLISTAGSLLPSVKSSHLVNPILGQLQSISGVGCATNLSVNNASGDDNNIKGETKLQCHLDQIEQVASWEREHKNAGKPIVSVSNVDEQIPKDEEDAGDADNLPPGSYEVLQLEKEEILAPHTHFCLICGKGFKRDANLRMHMRGHGDEYKTPAALAKPNKEPGTEPMLAKRYSCPYTGCKRNKDHKKFQPLKTFLCVKNHYKRTHCDKSYTCSRCNNKKFSVLADLKTHEKHCGRDKWLCSCGTTFSRKDKLFGHVALFQGHTPALPVEETKGACGTLNGEDSIEATNLENTGFNFSSGFSNESGVQNVIDMKGGIDDPAAFFSTLDFDTCSFGGYEFPRPPFDISESSLSCLLSGSSDYVQNTGTESGSDAFRIEKWKESSLAEDF
>S.bicolor_Sb03g041170
MEGRMTSLEAAMKASSSMASSMARNADPDHQTLRSNSVEQFYFPRPGQSLPGIPPFFGPPSSSLYPPNDNEAKFGSQFESNPSQNTDWDPQAIVSNLTFLEQKIKQVKDIVQSMNNRENQVAGGSSELAAKQQLITADLTSIIIQLISTAGSLLPSMKNPLLSSNPAVRQLGNTLGSPMGFGMNANQRPSVDSKTDIPDTGKTSDYDELMNSLNTAQDERDELIKCPNLCGGEGSEPTPMEDHDVKESDDGGEGENLPPGSYVVLQLEKEEILAPHTHFCLICGKGFKRDANLRMHMRGHGDEYKTPAALAKPTKDSGADHAPVTRYSCPFVGCKRNKEHKKFQPLKTILCVKNHYKRSHCDKSYTCSRCNTKKFSVIADLKTHEKHCGRDKWLCSCGTTFSRKDKLFGHVALFQGHTPALPMEDVKVSEASELPQDSEPTNEMARSMVYSFPSSSSDGISNLDMKMADDVRGYFSPLNFDPCFGALDDFTRPGFDISENPFSFLPSGSCSYGQQNGDS
>S.bicolor_ XP_002444574.1
MIGRGGNPYYLQNQQQQLFHHGHALDATMDGGGFMAEPPTPASSGCSAADAQCHALLYYNLSVLRDKVQQLQPLVGLAVAHDGPGPVAAAPGAGAVIQEIIAAASSMMYAFQQLCGHGGAVPASASATAAQAQQGGTSSGVVVADAAATCGAGDNHQHQQAAVIDHVMVMQQQWQQEHRYDGGYGGRIHHDSKTTTPVAAAAAMPSSSHPRPTTAAVMMAEEDEDVGVGVAGGTIIELEATELLAKYTHYCQVCGKGFKRDANLRMHMRAHGDEYKSSAALANPAKAAAAAGGDAAAASTSSSRSLYSCPQEGCRWNRKHAKFQPLKSVICAKNHYKRSHCPKMYVCNRCNRKHFSVLSDLRTHEKHCGDHRWLCSCGTSFSRKDKLVGHLALFTGHQPAVPLDRQANGGRRSSSSMSTSTQLDQTTNI 
>Z.mays_GRMZM2G068710
MDNHIHSQPAANLYYQFGMGTEQQYPPPFASPPSTNIPHMDWNPATMLDNLTFIEEKIQQVKDVIRTMVDNAGQVQVQCHHQAGELLAQKQKQQVVNADLTCLIVQLISTAGSLLPSLKNSSFLSHPHPAGHVDMANHVGPSSSLVPNAMTVSEDYEELFKGWTDGGIEVDDDGVLVEEQEITKDGDSLSYELLQLEEDEILAPHTHFCTICGKGFKRDANLRMHMRGHGDEYKSPAALAKPPRDPGAEQEPAKRRYSCPFAGCKRNKLHKSFQPLKTILCVKNHYRRSHCEKSHTCGRCHAKKFSIVADLRTHEKHCGRRDRWVCSCGTSFSRKDKLFAHVALFQGHTPALSSPLEEEPPKACSDQHQTGRAGSRREPAKVPAGSVGGGSGSSFVWGASSSGENSALDIKGFVDGCSGDFLSTANFGSFNLSFGPADGFTGEPSSGGSFSMLPPEHFQSAQKKGQN
>Z.mays_GRMZM2G075956
MEGRMTSLEATMKVSSSMASSISRNADPDQQTLRPNSVEQFYFPRPGQSLPGIPPFFGPPSSSLYLPNDNEAKFGNQFESNPSQNTDWDPQAIVSNLTFLEQKIKQVKDIVQSMSNRENQLAGGSSELAAKQHLVTADLTSIIIQLISTAGSLLPSMKNPLLSSNPAVRQLGNTLGSPMGLGMNANQRPSVDSKTDIPDTGKTSDYDELMNSLNPAQDERDEMIKCPNPCGGEGSEPTPMEDHDVKESDDGGEGENLPPGSYVVLQLEKEEILAPHTHFCLICGKGFKRDANLRMHMRGHGDEYKTPAALAKPTKDSGADHAPVTRYSCPFVGCKRNKEHKKFQPLKTILCVKNHYKRSHCDKSYTCSRCNTKKFSVIADLKTHEKHCGRDKWLCSCGTTFSRKDKLFGHVALFQGHTPALPMEDVKVSEASEQPQDSEPMNEMARSNMYSFPCSSSDGISNLDMKMADDVRGYFSPLNFDPCFGALDDFTRPGFDISENPFSFLPSGSCSYGQQNGDS
>S.italic_Si001061m
MEGGMSSLETAMKASSSIASGTARNAEPDQHPVCSNSLEQFYFPRPGQSLPGIPPFFGPPSSSLYLPNDNEAKVGNQFEPNPSQSTDWDPQAIVSNLTFLEQKIKQVKDVVQAMSNRENHVAGGSCELAAKQQLITADLTSIIIQLITTAGSLLPSMKNPLSSNLAVRQLGNTLGSPMGFGMNTNQRPSVDSKTNIPDSGKVSDYEELINSLNTTQDERDEMIKCPNPCVGEGSEPTPMEDHDVKESDDGEHEGENLPPGSYVVLQLEKEEILAPHTHFCLICGKGFKRDANLRMHMRGHGDEYKTAAALAKPTKDSGSEHAPVTRYSCPFVGCKRNKEHKKFQPLKTILCVKNHYKRSHCDKSYTCSRCNTKKFSVIADLKTHEKHCGRDKWLCSCGTTFSRKDKLFGHVALFQGHTPALPMDDVKISEASEQQQGSEPMNEISRSVGCFPCSSSDGISNLDMKMADDARGYYSPMSFDPCFGTLDDFTRPGFDISEDPFSFLPSGCSYVQQNGDN
>S.italic_Si010000m.g
MDHSQNHIHGQSAANFCYQFGSDNPFLGMGVQQPFASFTSPFGATPSTNIPHMDWNPATMLDNLTFIEEKIRQVKDVIRTMVDDGGQRPGELAQQQQVVNADLTCLIVQLISTAGSLLPSLKNSTFLSHPQAGHMGIVNHVGSSSSFVPNVTTISEENKEEMCGPEDYEELFKGFTDGALEGGIEIGNVLVEEQDAKDGDEGGDVGMDGESLPPGSYELLQLEKDEILAPHTHFCSICGKGFKRDANLRMHMRGHGDEYKSPAALAKPPRDASAEHELVRRYSCPFVGCKRNKLHKNFQPLKTILCVKNHYKRSHCEKSYTCSRCHTKKFSVMADLKTHEKHCGRDKWLCSCGTSFTRKDKLFAHVALFQGHTPALPTEEPKTSSDQISRVGSHQEPAKLPSSMGSSFVWGTSSGNENALDIKGVDGCSDDFLSTANFGSFNFSFGPADGFTGEPSGSSFSMLPSEHFQSAQKKGKN
>O.sativa_Os01g65080
MDSGLGRSSETSLKALPSMASNATRNTDPDQQGVRFSSMDQPPCFARPGQSFPAFPPLFGVQSSSLYLPDDIEAKIGNQFESNPSPNNPTMDWDPQAMLSNLSFLEQKIKQVKDIVQSMSNRESQVAGGSSEAQAKQQLVTADLTCIIIQLISTAGSLLPSMKNPISSNPALRHLSNTLCAPMILGTNCNLRPSANDEATIPDISKTHDYEELMNSLNTTQAESDEMMNCQNPCGGEGSEPIPMEDHDVKESDDGGERENLPPGSYVVLQLEKEEILAPHTHFCLICGKGFKRDANLRMHMRGHGDEYKTAAALAKPSKDSSLESAPVTRYSCPYVGCKRNKEHKKFQPLKTILCVKNHYKRSHCDKSYTCSRCNTKKFSVIADLKTHEKHCGRDKWLCSCGTTFSRKDKLFGHVALFQGHTPALPMDDIKVTGASEQPQGSEAMNTMVGSAGYNFPGSSSDDIPNLDMKMADDPRYFSPLSFDPCFGGLDDFTRPGFDISENPFSFLPSGSCSFGQQNGDS
> O.sativa_Os08g0562300
MIPGGGGGGGGGGISPYLVQSQHGHGGGVDGMEMEEGGGFMGEQPQCHPLLYNLSVLKDRVQQLHPLVGLAVAHNAHAHGPLDVSAADAIIQEIVAAASSMMYAFQLLCDLGTAPTTAPSQETAAASAVVVKNNDHAADAGQMEDDHLMQQQWQQNGSRQHDYSSHAHAPPVFHSETAAPAGATSATDTIIELDAAELLAKYTHYCQVCGKGFKRDANLRMHMRAHGDEYKSKAALSNPTKLLAKGGDETMAAAARKYSCPQEGCRWNRRHAKFQPLKSVICAKNHYKRSHCPKMYVCNRCGRKHFSVLSDLRTHEKHCGDHRWLCSCGTSFSRKDKLIGHVSLFAGHQPVMPLDAPRAGKRQRSSSASVAGNIDDTTGIGMGAA
>B.distachyon_Bradi2g56320v
MESGMRRSSETSVKALSSMASEASRNTDPGQQGLRFNSTDQSYFARAGQSIPVFPPFFGPQSSSFCLPDGSGAKVGGQFEPNPSLNNPISDWDPQAMLSNLSFLEQKIKQVKDVVQSMSNRGSQVVGGSGELAAKQQLVTADLTCIIIQLISTAGSMLPSMKSPLLSSNPAVRQLSNTLGSPMGFGSIANQRPSINKEQTIPDITKTSDYEELMNTINTTHDGKDDLIKCPNPCVGEGSDPIPFEDHDVKESDDGGEGENLPPGSYVVLQLEKEEILAPHTHFCVICGKGFKRDANLRMHMRGHGDEYKTPAALAKPMKDSSSDHTPVTRYSCPFVGCKRNKEHRKFQPLKTILCVKNHYKRSHCDKSYTCSRCNTKKFSVIADLKTHEKHCGRDKWLCSCGTTFSRKDKLFGHVALFQGHTPALPMDDIKGTCVSDQPEGSEVMDDMVGSTGYNFPGSASDGIPNLDMKVADDVRGYFSPLSFDPCFGALDDFTRPGFDISENPFSFLPSGPGSCSFGQPSGDS
>B.distachyon_ XP_003571264.1
MFSAGDDSGASPYFQQLFDHGDLGGADGIGAFAGGGDAQCQALLYNLSVLREKVRLLHPLVGLAVHGRGGVAVAADAGAVVQEIVAAASSMMYAFQHLCAVSDAAMQAQDSVAAAAGRAASNNAAGMAAAAAGCSDQQLQALEEDHEAATMQQWAHGGFYDDDGTSGSKPSAATAQQEAPAPAPGTKTRIIELDAAELLAKYTHYCKVCGKGFKRDANLRMHMRAHGDQYKSKAALSAVVSSSGASSSPAAMAASKYSCPQEGCRWNVRHARFTPLKSVICAKNHYRRSHCPKMYACSRCGRKQFSVLSDLRTHEKHCGDRRWLCSCGTTFSRKDKLAGHVSLFAGHHPVVVGEGARQCKIDRSSLANSDQLLGNCTTSAGDGFSIT
>T.aestivum_genome_A
MKASSSMASDASGNTDPGQQGARFSSMDQSCFARPGQSIPGYPPFFGPQSSNFYLPDDSVAKACDPFEPNPPQNNPVADWDPQAMLSNLTFLEQKIKQVKDIVQSMGNRGSQDVGGSCELAAKQQLVTADLTSIIIQLISTAGSMLPSMKTPLLSSNPAVRQLNTPGSPMGFGSIVNQRPSTVREEMVPDITKTPDYEDLMNTLNPAHDEKDDLIKCPNPCVGEGPEPVPMEDHDVKESDDGGEAEHLPPGSYVVLQLEKEEILAPHTHFCVICGKGFKRDANLRMHMRGHGDEYKTPAALAKPMRDSVSDPTPVTRYSCPYVGCKRNKEHRKFQPLKTILCVKNHYKRSHCDKRYTCSRCNTKKFSVIADLKTHEKHCGRDKWLCSCGTTFSRKDKLFGHVALFQGHTPALPMDDIKATGASEQRSEAMDDMVGSTGYNFPGSTSDGIPNLDMKVADDTRGYFSPLNFDPCFGALDDFARPGFDISENPFSFLPSGPGSCSFGQLSGDS
>T.aestivum_genome_B
MKASSSMASDASGNTEPGQQGVRFSSMDQSCFARPGQSIPGYPPFFGPQSSNFYLPDDSVAKACDPFEPNPPQSNPVADWDPQAMLSNLTFLEQKIKQVKDIVQSMGNRGSQDVGGSCELAAKQQLVTADLTSIIIQLISTAGSMLPSMKTPLLSSNPAVRQLNTPGSPMGFGSIVNQRPSTVREEMVPDISKTSDYEELMNTLNTAHDEKDDLIKCPNPCVGEGPEPVPMEDHDVKESDDGGEAEHLPPGSYVVLQLEKEEILAPHTHFCVICGKGFKRDANLRMHMRGHGDEYKTPAALAKPMRDSGSDPTPVTRYSCPYVGCKRNKEHRKFQPLKTILCVKNHYKRSHCDKRYTCSRCNTKKFSVIADLKTHEKHCGRDKWLCSCGTTFSRKNKLFGHVALFQGHTPALPMDDIKATGASEQPQGSEAMDDMVGSTGYNFPGSTSDGIPNLDMKVADDTRGYFSPLNFDPCFGALDDFARPGFDISENPFSFLPSGPGSCSFGQLSGDS
>T.aestivum_genome_D
MKASSSMASDASGNTEPGQQGVRFSSMDQSCFARPGQSIPGYPPFFGPQSSNFYLPDDSVAKACDLFEPNPPQNNPVADWDPQAMLSNLTFLEQKIKQVKDIVQSMGNRGSQDAGGSCELAAKQQLVTADLTSIIIQLISTAGSMLPSMKTPLLSGNPAVRQLNTPGSPMGFGSIVNQRPSTIREEVVNDISKTSDYEELMNTLNTAHDEKDDLIKCPNPCVGEGPEPVPMEDHDVKESDDGGEAEHLPPGSYVVLQLEKEEILAPHTHFCVICGKGFKRDANLRMHMRGHGDEYKTPAALAKPMRDPGSDPTPVTRYSCPYVGCKRNKEHRKFQPLKTILCVKNHYKRSHCDKRYTCSRCNTKKFSVIADLKTHEKHCGRDKWLCSCGTTFSRKDKLFGHVALFQGHTPALPMDDIKASGALEQPQGSEAMDDMVASTGYNFPGSTSDGIPNLDMKVADDTRGYFSPLNFDPCFGALDDFARPGFDISENPFSFLPSGPGSCSFGQLSGDS
>S.lycopersicum_AK320912.1
MDPDDSLSEDPWIKSSSSGNELLKIMPSDNHSFTNLNLHAQKWEGSSYLDQQIRIEQQFSGFAKPKHTSEMDQQGNQRNENHDTTRIHDWDPRALLNNLSFLEQKIHQLQELVHLIVGRRGQDEVQGNDLIVQQQQLITADLTSIIVQLISTAGSLLPTMKHTLSSAIPAASQLGQVGGVTVPSTAGTSAGGLTCNDGVAKLEDQSNHIDQLRDCGIEHNHAADEHELKDEDEAEEEENLPPGSYEILQLEKEEILAPHTHFCTICGKGFKRDANLRMHMRGHGDEYKTPAALAKPHKEPSSEPTLIKRYSCPYVGCKRNKEHKKFQPLKTILCVKNHYKRTHCEKAYTCSRCNIKKFSVIADLKTHEKHCGKDKWLCSCGTTFSRKDKLFGHLSLFQGHTPAVSPDETKGSAGTSDRGQTGEVTMKARQENYKVNASHGNEFQNPGVVKECPYNPSSYFSPLNFDTTNLNGFQEFPRPPFDESDSSFSFLLSGSCEYPPHKAAKFMSSSEME
> S.lycopersicum_ XP_004250985.1
MIQGTSSSANNPQNIPLYSSNHHPNNNIQEDIFSSSSSSYQQNSFLFNLSLLKEKVHQVQSLATMFITPDNQTIIHPPPESISMIIANMGTLIQEIITTSSSLMFSCQKIVLDSTSLSQNSSRYREPSQNDVGHGQGQGQVDHLLQDYDWYVDNYNSNCNTHEDNKNHVTSSSTIIASSTISHDNYYSKEFGKKELLLSTSKGKVVNEENNYDIVELDASDLLAKYTHYCQICGKGFKRDANLRMHMRAHGDEYKSSAALSNPMKRINDLTSDGSLKSSSNTIKYSCPQEGCRWNKKHAKFQPLKSMVCVKNHYKRSHCPKMYVCKRCNKKSFSVLSDLRTHEKHCGDLKWQCSCGTTFSRKDKLMGHVSLFVGHTPLIKQSAR
>H.vulgare_AK252406.1
MKASSSMAGDASGNTDPGQQGVRFSSMDQSCFARPGQSIPGYPPFFGPQSSNFYLPDDSVGKACDPFEPNPPVNEPIADWDPQAMLSNLTFLEQKIKQVKDIVQSMGNRGSQFGGASCELAAKQQLVTADLTSIIIQLISTAGSMLPSMKTPLLSSNPAVRQINTPGSTMGFGSTANQRPSATREEMIPDISKTSDYEELMNTLNTAHDEKDDQIKCPNPCGVEGSEPVPMEDHDVKESDDGGEAEHLPPGSYVVLQLEKEEILAPHTHFCVICGKGFKRDANLRMHMRGHGDEYKTPAALAKPMRDSGSDPTPVTRYSCPYVGCKRNKEHRKFQPLKTILCVKNHYKRSHCDKRYTCSRCNTKKFSVIADLKTHEKHCGRDKWLCSCGTTFSRKDKLFGHVALFQGHTPALPMDDIKATGASEQPQGSEAMDDMVGSTGYNFPGSTSDGVPNLDMKVADDTRGYFSPLNFDPCFGALDDFARPGFDISENPFSFLPSGPGSCSFGQLSGDN
>M.acuminata_Achr5P01170_001
MLSNLSFLEQKIHQVQDIVRSIISQEGRANELAAQQQLVTADLTYIIIQLISTAGTLLPNIKNALLSATPPVAQTDHDIKDYDDGMDGENLPPGSYEVLQLEKEEILAPHTHFCLICGKGFKRDANLRMHMRGHGDEYKTAAALAKPSKEASSEPAIIRRYSCPFVGCKRNKEHKKFQPLKTILCVKNHYKRSHCDKSYTCSRCKTKKFSVIADLKTHEKHCGRDKWICSCGTTFSRKDKLFGHVALFQGHTPAIPMDDAKVSGMSDHVQVGETTNGMVANMGYNFSGNASDDAQSLDIKDVDDGQGYFSPMNFDACNFGGLDEFPRSAYDVSESPFSFLSTGTCTNIFLFACGGHYTSFPKQKKKTIYDLLKGSSLFRSLPTLFIETCAFIN
>M.acuminata_Achr10P15970_001
MSSESRNLNQNQRATEFDARETEQSLPKFSSHLDNSFHQSNQAQRVAMNWDPRAVLNSLDILGQKIHQIQDIVRSTLSDERQLSIQPNEFAAQQQLINTDLTCTIIELISTAGTLLPLIKNALASGINSSGQIDNDVKDNEDGVSGENLPLGSYEVLQLEKEEILAPHTHFCAICGKGFKRDANLRMHMRGHGDEYKSPSALAKPTKEGSSEPVPIKRYSCPFIGCKRNKEHKNFQPLKTILCVKNHYKRSHCDKSYTCRRCNSKKFSVMADLKTHEKHCGSNKWLCSCGTTFSRKDKLFGHIALFQGHTPALSMDEVKSQGMSVQGQSDEVMAKEEDIDYLISGNVIEDTNFSGLNGTDNDLGYFSSMNFDSFTFGGIDGLQQPSFDTSESLFSFHK
>M.acuminata_Achr6P18840_001
MLSNLSFLEQKIHQVQDIVRSIISHEDQFLDQANELAAQQQLVTADLTYIIIQLISTAGTLLPDIKNALLSTNPAVGRPGSNQKQHEILPVEVAKASQYDSLIKDLNSCGGKEDELIKCSNNSGVEASEPIPIEDHDTKDNDDGMDGENLPPGSYEVLQLEEEEILAPHTHFCLICGKGFKRDANLRMHMRGHGDEYKSPAALAKPSKEASIEPQPLRRYSCPFVGCKRNKEHKKFQPLKTILCVKNHYKRSHCDKSYTCSRCRTKKFSIIADLKTHEKHCGRDKWICSCGTTFSRKDKLFGHVALFQGHAPAIPMDDAKVLVTSDKAQVSEASSGMPGNMGYNFSGNTSLDAQSPDIKGVDDGHGCFSPMTLDACNIWGLAEFPHSAYEVSESTFSFLATGSCSSIQRNGENGFDISENPFSFLPSGPGSCSFGQLSGDN
>T.urartu_EMS66742.1
MASDASGNTDPGQQGARFSSMDQSCFARPGQSIPGYPPFFGPQSSNFYLPDDSVAKACDPFEPNPPQNNPVADWDPQAMLSNLTFLEQKIKQVKDIVQSMGNRGSQDVGGSCELAAKQQLVTADLTSIIIQLISTAGSMLPSMKTPLLSSNPAVRQLNTPGSPMGFGSIVNQRPSTVREEMVPDITKTPDYEDLMNTLNPAHDEKDDLIKCPNPCVGEGPEPVPMEDHDVKESDDGGEAEHLPPGSYVVLQLEKEEILAPHTHFCVICGKGFKRDANLRMHMRGHGDEYKTPAALAKPMRDSVSDPTPVTRYSCPYVGCKRNKEHRKFQPLKTILCVKNHYKRSHCDKRYTCSRCNTKKFSVIADLKTHEKHCGRDKWLCSCGTTFSRKDKLFGHVALFQGHTPALPMDDIKATGALEQPQGSEAMDDMVGSTGYNFPGSTSDGIPNLDMKVADDTRGYFSPLNFDPCFGALDDFARPGFDISENPFSFLPSGPGSCSFGQLSGDS
>T.urartu_EMS47263.1
MFPDAGASPYFLQNQQQLFHGMNGIGVDAAFPGDGGGLAVPDSTHRSTLLYNLSVLKDKVQQLEPLVGHGHVVDPVVPGASAVVQEIIAAATSMLYALQHPYVLGALTTSGNHAATPEGPTAVDTAIIELDAAELLAKYTHYCQVCGKGFKRDANLRMHMRAHGDEYKSKAALANPTTRLFATSGEDAAAGRPRNSKYSCPQDGCRWNRRHAKFQPLKSVICAKNHYKRSHCPKMYVCNRCNRKHFSVLSDLRTHEKHCGDHRWICSCGTTFSRKDKLAGHVSLFAGHQPVAPLAPGSGRHGKRSSLLSPSSDDLAGNCTNTGFSITPTT
>Ae.Tauschii_EMT26835.1
MASDASGNTEPGQQGVRFSSMDQSCFARPGQSIPGYPPFFGPQSSNFYLPDDSVAKACDLFEPNPPQNNPVADWDPQAMLSNLTFLEQKIKQVKDIVQSMGNRGSQDAGGSCELAAKQQLVTADLTSIIIQLISTAGSMLPSMKTPLLSSNPAVRQLNTPGSPMGFGSIVNQRPSTIREEVVNDISKTSDYEELMNTLNTAHDEKDDLIKCPNPCVGEGPEPVPMEDHDVKESDDGGEAEHLPPGSYVVLQLEKEEILAPHTHFCVICGKGFKRDANLRMHMRGHGDEYKTPAALAKPMRDSGSDPTPVTRYSCPYVGCKRNKEHRKFQPLKTILCVKNHYKRSHCDKRYTCSRCNTKKFSVIADLKTHEKHCGRDKWLCSCGTTFSRKDKLFGHVALFQGHTPALPMDDIKASGALEQPQGSEAMDDMVASTGYNFPGSTSDGIPNLDMKVADDTRGYFSPLNFDPCFGALDDFARPGFDISENPFSFLPSGPGSCSFGQLSGDS
>A.lyriata_XP_002874102.1  
MNRDEHITNKGEGSMSSFSGTTSTSSKQISLNPVTAAGAKSLEEDDVSLSLLYNLSTLQDKVHQIQSLVSFYMISSNNINQYSGSTSLAVANIGSLVQEIITAASSMLYTCQQLHIGSNNSNDIDNDHTVDAMVLEFSRQETDPGHDFVQESTNLFGVQERGHVSFPNQNHDWYDTETLNPKKDKHRSKPKPGNYEILELDVADLLAKYTHYCQICGKGFKRDANLRMHMRAHGDEYKTREALISPTSQEKKGEYTLKKHYYSCPHQGCRWNQRHEKFQPLKSVICAKNHYKRSHCPKMYMCRRCSVKHFSVLSDLRTHEKHCGDIKWVCSCGTKFSRKDKLMSHVSLFLGHVPAHGSSQPPTTITLK
>F.Vesca_XP_004294539.1
MNINQGSTSNCFESMYMTDRDAVSSFSLEPNSSSSGAVLSQDQNHSTSLLYSLSVLKEKVHQAKSLVGVLIAPADHHQSQQQQLPESTTMGAMASMSNVIQEIIVTASSMMFTCQQMSLVAPGNDTRSEPNFTISTAGRSNTRDAGFYPTHDQTLDWFSTESYNDCLPRDVISRSHRQVETKELPPRVQRNNALQVENPPPENSSYDIIELDAADLLAKYTHYCQVCGKGFKRDANLRMHMRAHGDEYKTSIALSNPLKNNNMNSAMNVNGSSNAESSMKLPRKYSCPQEGCRWNQKHAKFQPLKSMICVKNHYKRSHCPKMYVCKRCNRKQFSVLSDLRTHEKHCGDLRWQCSCGTTFSRKDKLMGHVALFVGHTPVIINSNFPIRHGRAESQAAAGGLMQ
>F.Vesca_XP_004290434.1
MDHKDKVCADTWAKDLRNKTCSDRPKFANFSAHQHQNKWEDPSILDYGIRIEPSFQKLSQPSEDQTSLPHNSNNEKTIADGEDVQMNEIFHASKIQDWDPSTMLTNLSFLEQKIHQLQDLVHLIVGRRGQVLGRPDELVAQQQQLITADLTSIIAQLISTAGSLLPSVKHTLSSASASAVQFGQLGGSFVSSGAGTEASVKLQMNCGSKLPEQPNQTDPISNYGTELNYHIEEHESKDEEDADEGENLPPGTYEILQLEKEEILAPHTHFCAICGKGFKRDANLRMHMRGHGDEYKTPAALAKPNKDSSSEPTLIKRYSCPYAGCKRNKDHKKFQPLKTILCVKNHYKRTHCDKSYTCSRCNTKKFSVIADLKTHEKHCGKDKWLCSCGTTFSRKDKLFGHIALFQGHTPAIPLDETKTAVGASEHGEGSEAPNRVGSINFNFGSTLPSAGGLVQNIMDVKESVDDPTCYFSPLNFDTCNFDGFHEFPRPPFEDSESSFSFLMPGSCNYTHKTGGESNSKQVE
